# A New Chicken Genome Assembly Provides Insight into Avian Genome Structure

**DOI:** 10.1534/g3.116.035923

**Published:** 2016-11-14

**Authors:** Wesley C. Warren, LaDeana W. Hillier, Chad Tomlinson, Patrick Minx, Milinn Kremitzki, Tina Graves, Chris Markovic, Nathan Bouk, Kim D. Pruitt, Francoise Thibaud-Nissen, Valerie Schneider, Tamer A. Mansour, C. Titus Brown, Aleksey Zimin, Rachel Hawken, Mitch Abrahamsen, Alexis B. Pyrkosz, Mireille Morisson, Valerie Fillon, Alain Vignal, William Chow, Kerstin Howe, Janet E. Fulton, Marcia M. Miller, Peter Lovell, Claudio V. Mello, Morgan Wirthlin, Andrew S. Mason, Richard Kuo, David W. Burt, Jerry B. Dodgson, Hans H. Cheng

**Affiliations:** * McDonnell Genome Institute, Washington University School of Medicine, St. Louis, Missouri 63108; † National Center for Biotechnology Information, National Library of Medicine, National Institutes of Health, Bethesda, Maryland 20894; ‡ University of California-Davis, California 95616; § Institute for Physical Sciences and Technology, University of Maryland, College Park, Maryland 20742; ** Cobb-Vantress Inc., Siloam Springs, Arkansas 72761-1030; †† United States Department of Agriculture-Agricultural Research Service, Avian Disease and Oncology, East Lansing, Michigan 48823; ‡‡ Génétique Physiologie et Systèmes d’Elevage, Université de Toulouse, Institut National de la Recherche Agronomique, Auzeville Castanet Tolosan, France; §§ Wellcome Trust Sanger Institute, Cambridgeshire CB10 1SA, United Kingdom; *** Hy-Line International, Dallas, Iowa 50063; ††† Beckman Research Institute, Duarte, California 91010-3000; ‡‡‡ Department of Behavioral Neuroscience, Oregon Health and Science University, Portland, Oregon 97239-3098; §§§ The Roslin Institute and Royal (Dick) School of Veterinary Studies, University of Edinburgh, Midlothian EH25 9RG, United Kingdom; **** Department of Microbiology and Molecular Genetics, Michigan State University, East Lansing, Michigan 48824

**Keywords:** *Gallus gallus*, genome assembly, MHC

## Abstract

The importance of the *Gallus gallus* (chicken) as a model organism and agricultural animal merits a continuation of sequence assembly improvement efforts. We present a new version of the chicken genome assembly (Gallus_gallus-5.0; GCA_000002315.3), built from combined long single molecule sequencing technology, finished BACs, and improved physical maps. In overall assembled bases, we see a gain of 183 Mb, including 16.4 Mb in placed chromosomes with a corresponding gain in the percentage of intact repeat elements characterized. Of the 1.21 Gb genome, we include three previously missing autosomes, GGA30, 31, and 33, and improve sequence contig length 10-fold over the previous Gallus_gallus-4.0. Despite the significant base representation improvements made, 138 Mb of sequence is not yet located to chromosomes. When annotated for gene content, Gallus_gallus-5.0 shows an increase of 4679 annotated genes (2768 noncoding and 1911 protein-coding) over those in Gallus_gallus-4.0. We also revisited the question of what genes are missing in the avian lineage, as assessed by the highest quality avian genome assembly to date, and found that a large fraction of the original set of missing genes are still absent in sequenced bird species. Finally, our new data support a detailed map of MHC-*B*, encompassing two segments: one with a highly stable gene copy number and another in which the gene copy number is highly variable. The chicken model has been a critical resource for many other fields of study, and this new reference assembly will substantially further these efforts.

The initial genome sequence of a single, partially inbred, Red Jungle Fowl (the primary wild progenitor of domestic chickens) female provided a substantial advance for avian genetics, enabling a range of new “omics” analyses and technologies to be applied (International Chicken Genome Sequencing Consortium 2004). Over the years, further improvements were made to the avian genome reference assembly (Schmid *et al.* 2015). A second build (Gallus_gallus 2.1; GCA_000002315.1) of the original generated in 2004 improved assembly base presentation, order, and orientation using new single nucleotide polymorphism (SNP) mapping data and targeted sequencing of BACs and fosmids. The total sequence in Gallus_gallus 2.1 measured 1.09 Gb, ∼95% of which was anchored to autosomes 1–28 and 32, along with the GGAZ and GGAW sex chromosomes.

The chicken karyotype includes 38 autosomes, many of which are relatively small and uniform in size, often termed microchromosomes. Although the demarcation of microchromosomes has varied between authors, it is clear that several properties (*e.g.*, %GC, gene, and repeat density) differ between macrochromosomes and microchromosomes (International Chicken Genome Sequencing Consortium 2004), and these differences likely contribute to the fact that some microchromosomes are not assembled or are only partially or incorrectly assembled (International Chicken Genome Sequencing Consortium 2004; Gordon *et al.* 2007). Furthermore, the first versions (International Chicken Genome Sequencing Consortium 2004) of GGAZ and GGAW were sequenced only to ∼3.3× due to their hemizygous state in the female bird used. However, a BAC-focused effort increased the size of GGAZ from 33.6 (International Chicken Genome Sequencing Consortium 2004) to 81.8 Mb (Gallus_gallus_4.0) (Bellott *et al.* 2010), while increasing GGAW from 858 kb (Gallus_gallus-2.1) to 2.2 Mb (Gallus_gallus-4.0).

A third build of the genome (Gallus_gallus-4.0; GCA_000002315.2) included the use of next-generation sequencing technology (∼12× of 454 Titanium sequences) in combination with the earlier Sanger sequenced reads. An increase of N50 contig and scaffold size to 252 kb (460%) and 12.4 Mb, respectively, was observed. In addition, the new assembly mostly removed ∼10 Mb of artefactual duplications noted in earlier comparative assessments (Rubin *et al.* 2010), likely due to the fact that the sequenced bird was only partially inbred and thus retained regions of allelic diversity that were mistakenly assembled as independent loci. The total amount of sequence mapped to the chromosomes increased by 15 Mb, after accounting for duplication errors.

Despite the substantial improvements represented in Gallus_gallus-4.0, several assembly issues persist that limit use of this critical resource. The Gallus_gallus-4.0 assembly has 8246 estimated spanned gaps on the ordered and oriented chromosome sequences, lacks chromosome sequence assignment altogether for 3.1% (32 Mb), and still retains 0.4% of likely artificially duplicated sequences in the form of nearly identical (99.9%) tandem duplications (Zhang and Backstrom 2014). In addition, nine microchromosomes (29–31, 33–38) that are thought to be gene-rich are either not assembled or assigned, which is due mostly to a lack of genetic linkage group markers to anchor unplaced sequences. Therefore, geneticists are unable to map variations of potential importance which hinders scans of natural or artificial selection (Rubin *et al.* 2010; Qanbari *et al.* 2015; Reyer *et al.* 2015)

To address the need for a more complete chicken genome assembly, we have updated the chicken reference genome assembly by sequencing the chicken genome to deep coverage (∼51×) with long single molecule technology, assembling these reads into chromosomes, and report a careful examination of the features of this improved reference.

## Materials and Methods

### Sequencing

Total DNA was obtained from the same bird (#256) of the UCD001 line, a Red Jungle Fowl laboratory line, used to create all prior versions of the chicken reference genome assembly (International Chicken Genome Sequencing Consortium 2004). All sequences were generated on a PacBio RSII instrument first using P4/C2, then transitioning to P5/C3 sequencing chemistry (Bioproject PRJNA10808). Total estimated coverage by sequencing chemistry was ∼34% and 66% for P4/C2 and P5/C3, respectively. Sequencing coverage was targeted to be 50× total, using a 1.25 Gb estimated genome size for the Galliformes family (Venturini *et al.* 1986).

### Assembly

Sequences were assembled using the PBcR-MHAP algorithm version 8.2 (Berlin *et al.* 2014). PBcR-MHAP assemblies were derived from a total of 18.7 Gb corrected sequence data, which is estimated to represent ∼15.3× coverage, based upon the size of the Gallus_gallus-5.0 assembled reference. To scaffold the *de novo* assembled contigs, we first completed an iterative series of joins using paired-end sequences with 3 and 40 kb (fosmids) insert length, followed by joins made with BAC (CHORI-261) end sequences (BES) utilizing the SSPACE tool version 3.0 (Boetzer and Pirovano 2014). SSPACE also merges contigs and therefore the contig number is expected to be slightly less afterward. At this stage, to ensure the best genome representation even at the expense of scaffold contiguity, we merged Gallus_gallus-4.0 into Gallus_gallus-5.0 with the GAA merger tool (Yao *et al.* 2012). The final scaffolded assembly was used as input to align against Gallus_gallus-4.0 using Nucmer, an aligner module part of the MUMmer version 3.0 (Kurtz *et al.* 2004), to create chromosome files and define the unplaced sequences (see Supplemental Material, Table S1 for main stages of assembly). At this stage, the assembly was error-corrected for single base errors and insertions or deletions (indels) using 36× sequence coverage of paired-end Illumina sequences (100 bp length; accession no. SRR3954707) generated from the same DNA source as the Gallus_gallus-4.0 reference, using ICORN2 version 0.95 (Tsai *et al.* 2010). Finished clones from the CHORI-261 (*n* = 168) library (Schneider *et al.* 2013) were then aligned to only assembled autosomes with NG Aligner, an NCBI-developed BLAST-based alignment algorithm (http://www.ncbi.nlm.nih.gov/IEB/ToolBox/CPP_DOC/doxyhtml/ngalign__tool_8cpp_source.html). Alignments with <98% identity and <95% coverage were discarded. As a result of filtering, all remaining clone sequences were uniquely aligned and replaced the underlying local autosomal assembly. In contrast, GGAW was generated by aligning and integrating whole genome sequence (WGS) sequences from the GGAZ, GGAW, and unlocalized WGS-only assemblies into tiling path backbones comprised of finished clones from the following clone libraries: CHORI-261 (GGAW and GGAZ), TAM31 (GGAZ), TAM32 (GGAZ), TAM33 (GGAZ), J_AE (GGAZ), and J_AD (GGAW and GGAZ). The Gallus_gallus-5.0 BAC tiling path for GGAZ is unchanged from Gallus_gallus-4.0. The BAC tiling path for GGAW was manually determined by evaluating BAC fingerprint maps (Wallis *et al.* 2004) and BES placements on the Gallus_gallus-5.0 assembly. Assembled WGS additions to the sex chromosomes did not replace any previously established assembled BAC sequence, but were used to close or reduce gaps between BAC clones. Utilizing unpublished radiation hybrid map data, with >50 markers, we assigned the E22C19WAU28 linkage group to GGA33.

To identify putative misassemblies in the Gallus_gallus-5.0 assembly, we aligned it to Gallus_gallus-4.0 [a previously defined linkage map (Groenen *et al.* 2009)], and to end sequences from the CHORI-261 library (a set of end sequences not directly incorporated into the assembly; *n* = 116,288). For all assembly-to-assembly alignments, each Gallus_gallus-5. 0 or 4.0 chromosome was broken into nonoverlapping 1-kb segments and aligned to identify uniquely aligning segments, using BLAT (Kent 2002). Linkage map markers (Groenen *et al.* 2009) were aligned to the assembly using BLAT. The marker data alone ordered and oriented 233 scaffolds spanning 912 Mb of the sequence. All major discrepancies were resolved after reviewing all available data. CHORI-261 BES were screened prior to alignment to remove low quality bases and vector contamination, and then aligned to a repeat-masked copy of Gallus_gallus-5.0 (Schneider *et al.* 2013). Only clone placements comprised of correctly oriented BES placements and having a length within three SD of the library average insert size were considered concordant. A detailed description of clone placement methods was documented earlier (Schneider *et al.* 2013). To further measure chromosome completeness, we aligned the bird #256 Illumina sequences (accession no. SRR3954707) and an independent bird (accession no. ENA PRJEB15276) to Gallus_gallus-4.0 or Gallus_gallus-5.0, using BWA-MEM (Li and Durbin 2009).

### Retrotransposons

Long Terminal Repeat (LTR) retrotransposons were identified in the Gallus_gallus-5.0 assembly with an iterative pipeline LocaTR (Mason *et al.* 2016) that incorporates LTR_STRUC (McCarthy and McDonald 2003), LTR Harvest (Ellinghaus *et al.* 2008), MGEScan_LTR (Rho *et al.* 2007), and RepeatMasker (Smit 2013) search algorithms. These data were compared to a recent LTR annotation of the Gallus_gallus-4.0 assembly (Mason *et al.* 2016).

### Variant detection

To call SNPs and indels for both assemblies, we aligned 36× sequence coverage of Illumina data (bird #256; accession no. SRR3954707) using a convergence of variants approach with independent calls from SAMtools (Li and Durbin 2009) and VarScan2 variant calling software (Koboldt *et al.* 2013), as described earlier (Montague *et al.* 2014).

### Gene annotation

The Gallus_gallus-5.0 assembly was annotated using the NCBI Eukaryotic Annotation Pipeline (https://www.ncbi.nlm.nih.gov/books/NBK169439/). The assembly was masked with Windowmasker (Morgulis *et al.* 2006), which identified 24.5% of the genome as repetitive. The annotation process was initiated by aligning avian proteins, and *Gallus gallus* transcripts (cDNA and ESTs) and RNA-Seq data to the masked genome (Kapustin *et al.* 2008). In total, >9.2 billion RNA sequences were retrieved from the NCBI Sequence Archive (see annotation report at http://www.ncbi.nlm.nih.gov/genome/annotation_euk/Gallus_gallus/103/) and aligned to Gallus_gallus-5.0, of which overlapping RNA-Seq data, transcript, and protein alignments with compatible splice patterns and coding frames were merged into chains by the Chainer component of Gnomon. Chains representing incomplete models (missing a stop or a start codon or internal exons) but with high coding propensity scores were then extended into complete models using the *ab initio* component of Gnomon. Gnomon predictions with the same splice pattern as an aligned known RefSeq transcript were discarded along with Gnomon predictions deemed low confidence based on several criteria, including evidence support, number of exons, conflicting prediction on the other strand, and for high *ab initio* models (>50%), homology to a UniProtKB/SwissProt. The remaining set of Gnomon models were assigned functional annotation by orthology to human version GRCh37 and homology to known UniProtKB/SwissProt models, and finally assigned Gene IDs and accessions (XM_, XP, XR_) before being loaded onto the NCBI Nucleotide and Protein databases. The final annotation, comprising the RefSeq models and the placed known RefSeq, were published on the NCBI FTP site as annotation release 103.

### Gene representation

To better understand differences in gene content between Gallus_gallus-4.0 and Gallus_gallus-5.0, we aligned the two assemblies to each other using BLAST, and identified the reciprocal best hits as matching regions. The overlap in the annotated features on Gallus_gallus-4.0 (release 102) and Gallus_gallus-5.0 (release 103) in the matching regions were analyzed. Scores for overlapping current and previous gene and transcript features were calculated based on overlap in exon sequence and matches in exon boundaries and used to characterize the change in each pairs of current and previous features. The predicted protein sequences corresponding to newly predicted coding genes were retrieved from the NCBI proteome (ftp://ftp.ncbi.nih.gov/genomes/Gallus_gallus/protein/protein.fa.gz) and scanned for conserved Interpro signatures in 11 databases of protein predictive models (Mitchell *et al.* 2015) (File S1 and Table S2). Blast2GO (Gotz *et al.* 2008) suite (v3.2) was used to perform comprehensive gene ontology (GO) analysis (Huntley *et al.* 2015). Initially, GO terms were assigned to novel proteins based on sequence similarity to other proteins in Swiss-Prot (Boutet *et al.* 2016). The results were integrated with ontology terms predicted by InterProScan version 5 analysis (Jones *et al.* 2014). Finally, Annex-based GO term augmentation was done, making use of univocal relationships between the three GO categories (Myhre *et al.* 2006; Gotz *et al.* 2008).

In an attempt to define how many transcripts remain missing or are partial copies in Gallus_gallus-5.0, we aligned the chicken RefSeq transcripts (*n* = 6994) using Splign (Kapustin *et al.* 2008), with minimum exon identity set to 75%. In addition, we revisited our earlier study that defined a set of high confidence missing avian genes relative to other closely related sauropsids (Lovell *et al.* 2014) to further characterize gene representation. To search for missing genes, we used previously defined BLAT (Kent 2002)/BLAST search parameters (Lovell *et al.* 2014). We also searched NCBI’s Entrez gene for entries corresponding to the set of 571 gene model predictions that we previously found to be missing in Gallus_gallus-4.0 (Lovell *et al.* 2014). We also examined another 143 genes that were previously found to be partial and highly truncated, or that were only present on unplaced segments in Gallus_gallus-4.0. Entries that matched according to gene name (*i.e.*, Gene Symbol) or gene description were retrieved for further verification. We next manually examined each matching entry from the missing gene list and confirmed its orthology with the correct human (GRCh37) or lizard (anoCar2) ortholog by searching for evidence of complete or partial synteny. However, in cases where synteny analysis was not possible, we used a criterion of reciprocal best BLAT alignments of nucleotide and/or protein sequences against the human and lizard genome assemblies, or best BLASTn (Chen *et al.* 2015) alignment to identify nucleotide sequences in NCBI’s database for future correction.

To further exhaust the possibility that some of the previously missing genes in Gallus_gallus-4.0 might be hidden in Gallus_gallus-5.0, but are either not predicted or are represented by unannotated or incorrectly annotated Entrez gene entries, we conducted a systematic BLAST search of Gallus_gallus-5.0 using probes derived from synteny-verified orthologs from the closest avian relatives (*e.g.*, turkey, duck, and goose), as well as orthologous genes in other bird species (*e.g.*, common starling, Tibetan tit) or in lizard, alligator, and human genomes. All BLAST searches were conducted using conservative parameters (Block Substitution Matrix 45) for highly divergent sequences, and significant hits were manually verified by synteny analysis and/or reciprocal best BLASTn searches of nucleotide databases, using segments of the chicken genome as queries.

### Immune regions

The NCBI Genome Workbench tool (version 2.10.0) was used to compare the alignments between an assembled region of the chicken major histocompatibility complex (MHC) from bird #256 (accession no. AB268588.1) and chromosome 16 sequences in both Gallus_gallus-4.0 and Gallus_gallus-5.0 in a MegaBLAST (Johnson *et al.* 2008) search. Alignments were generated, and adjacent fragments of alignments were merged and redundant fragments removed. An alignment span view table displays the gaps, indels, and stretches of complete alignment regions for each of the two assembled versions (Figure S1).

To assess the inclusion of CHIR loci in Gallus_gallus-5.0, we used seven CHIR loci sequences from the WAG BAC clones (WAG-112A23, WAG-19H9, WAG-4C11, WAG-52G8, WAG-58B13, WAG-88M21, and WAG-93H17) and mapped them onto Gallus_gallus-5.0 using BLASTn. These WAG BAC clones were not constructed from bird #256 DNA and so only served to verify representation, not haplotype order. Since the CHIR loci were assigned by FISH to microchromosome 31 (Viertlboeck *et al.* 2005), we used a group of 84 predicted CHIR transcripts and aligned all to the Gallus_gallus-5.0 version of GGA31 or the associated unlocalized scaffolds, including the linkage group LGE64, using GMAP (Wu *et al.* 2016).

### Genetic linkage mapping

Genotyping was performed to identify new linkage groups and to aid the genome assembly using the East Lansing (EL) mapping population created with individuals from the UCD001 and UCD003 lines (Cheng *et al.* 1995). Unassigned sequence contigs of ≥2 kb were identified and, with existing Illumina reads from UCD003, were aligned to identify SNPs using the same process as described for variant detection (Cheng *et al.* 1995). All SNPs were evaluated using the Affymetrix Axiom myDesign Array pipeline (Affymetrix, Santa Clara, CA) to develop a custom array of 60,000 SNPs. We assigned a minimum of two or more SNPs per unplaced contig, and required known SNPs to be equally spaced throughout the Gallus_gallus-4.0 assembly. To generate the linkage map, the resulting genotypes from 88 EL progeny and prior EL genetic markers were mapped using MapManager QTX (Manly *et al.* 2001).

### Data availability

The authors state that all data necessary for confirming the conclusions presented in the article are represented fully within the article.

## Results

### Genome assembly representation

We generated 50.6× sequence coverage of long single molecule sequences, specifically termed subreads (bird #256; PRJNA10808) of differing sequencing chemistry that were comprised of 34% P4/C2 (21 Gb; 4024 bases average reads of insert length) and 66% P5/C3 (40.9 Gb; 7505 bases average reads of insert length) reads. Using all long single molecule sequences, we error-corrected a total of 18.7 Gb (15.3× sequence coverage) using a module within the MHAP/PbCR algorithm (Koren *et al.* 2012). All error-corrected sequences were originally assembled into contigs to a total size of 1.21 Gb, with an N50 contig length of 1.07 Mb (Table S1). A secondary correction of residual indels and single bases using aligned reads (bird #256; accession no. SRR3954707) and the ICORN2 tool (Tsai *et al.* 2010) replaced a total of 1,191,673 insertions, 870,666 deletions, and 561,565 single base sites. After gap closing, additional scaffolding with fosmid and BES, and finally, merging of Gallus_gallus-4.0 sequences, we achieved a final assembly contiguity of 1.23 Gb with an N50 contig and scaffold length of 2.9 and 6.4 Mb, respectively (Table 1 and see Table S1 for iterative build statistics). Overall scaffolding continuity decreased in Gallus_gallus-5.0 compared with Gallus_gallus-4.0, despite Gallus_gallus-4.0 starting at a twofold higher point (12 Mb). We believe this is driven mostly by merging smaller unplaced sequence scaffolds and the assembly gain of a much higher amount of unplaced sequences in Gallus_gallus-5.0 (138 *vs.* 61 Mb), a benefit of capturing more challenging sequence architecture with the long read sequencing technology. Unplaced scaffold N50 length was 4.6 and 12.5 kb in the Gallus_gallus-4.0 and Gallus_gallus-5.0, respectively. Our alignment of Gallus_gallus-4.0 recovered ∼13 Mb of unique sequences not assembled in the second draft assembly (Table S1). Our Gallus_gallus-5.0 assembly represents a >10-fold increase in the size of the N50 contig length with a gain of 183 Mb of new sequence and only 4.3% as many spanned (paired reads define the gap within a scaffold) assembly gaps (*n* = 358) among placed (ordered and oriented) chromosomal sequences as compared with Gallus_gallus-4.0 (*n* = 8246). Gallus_gallus-5.0 also contains 2348 fewer contigs (24,693 total contigs) than Gallus_gallus-4.0 (27,041 total contigs).

**Table 1:** Assembly contiguity metrics of chicken genomes by version

Metrics	Gallus_gallus-4.0	Gallus_gallus-5.0
Total base length (bp)	1,046,932,099	1,230,258,557
Total spanned gaps*^a^*	8246	358
Total contigs	27,041	24,693
N50 contig length (bp)*^b^*	279,750	2,894,815
Placed contigs (bp)*^c^*	1,014,655,963	1,091,712,069
Unplaced contigs (bp)	32,120,124	138,199,872
Total scaffolds	16,847	23,870
N50 scaffold length (bp)*^b^*	12,877,381	6,379,610

a Total spanned gaps are calculated on ordered and oriented chromosome sequences.

b N50 is the percentage of the genome assembly that is the measured length metric or greater.

c Placed contigs refer to chromosome sequences with order and orientation or just chromosomal assignment without order or orientation.

For all Gallus_gallus-5.0 sequences 6.5% (81 Mb) has been assigned to a chromosome but with no order or orientation, compared with 2.1% (20.9 Mb) in Gallus_gallus-4.0. Among sequences with chromosomal order and orientation, we observe a gain of 16.4 Mb in new sequence not found in Gallus_gallus-4.0 (Figure S2). For the sex chromosomes GGAW and GGAZ, we see total increases of 3.46 Mb and 98 kb, respectively. The small incremental gain in GGAZ sequence is due to the prior Gallus_gallus-4.0 version of GGAZ being a BAC-based high quality assembly (Bellott *et al.* 2010). Despite a substantial sequence gain in GGAW, we still estimate only ∼22% of the predicted 30 Mb GGAW chromosome (D.W. Bellott, H. Skaletsky, T. Cho, L. Brown, D. Locke, N. Chen, S. Galkina, T. Pyntikova, N. Koutseva, T. Graves, C. Kremitzki, W.C. Warren, A.G. Clark, E. Gaginskaya, R.K. Wilson, and D.C. Page, unpublished results) is assembled. Only GGA24 experienced a slight decrease (2.6 kb) of sequence in Gallus_gallus-5.0 (Figure S2). Another measure of completeness is the percentage of same source sequences (bird #256; accession no. SRR3954707) that align to each Gallus_gallus-5.0 autosome. Overall, the average aligned coverage of each autosome was 97.5%, with low coverage of two autosome outliers, GGA22 (88.7%) and GGA25 (80.2%) (Figure S3). We find these chromosome coverage anomalies in Gallus_gallus-5.0 come from added sequences that are rich in GC content and thus not sequenced with Illumina technology. For example, we add unordered sequences (∼640 kb) to GGA22 that are 54% GC but 42% GC in the ordered and oriented classification. Similar to our earlier findings (International Chicken Genome Sequencing Consortium 2004), we find the microchromosomes show higher GC content (48%) than the macrochromosomes (40%), with some autosomes displaying extremes, *e.g.*, GGA33 (1.6 Mb) with 56% GC composition (Figure S4 and Figure S5).

### Repeats

A total of 34.8 Mb of the Gallus_gallus-5.0 assembly was identified as LTR retrotransposon using the LocaTR pipeline (Mason *et al.* 2016), which accounts for 2.83% of the genome and is 3.4 Mb more than in Gallus_gallus-4.0. This, as well as greater annotation of Chicken Repeat 1 (CR1) LINEs, brings the total chicken repeat content to over 200 Mb (16.4%), >70 Mb more than in Gallus_gallus-4.0 (Table 2).

**Table 2:** A comparative summary of assembled repeat content

	Gallus_gallus-4.0	Gallus_gallus-5.0
Total repeat content	130,832,793 (12.5%)	202,236,305 (16.4%)
CR1 copy number	194,734	225,230
LTR retrotransposon content	31,490,117 (3.0%)	34,811,469 (2.8%)
Intact LTR retrotransposons	1073	1212

While the majority of the 63,651 annotated LTR retrotransposon elements were fragmented, 1212 structurally intact elements were identified; 139 more than in Gallus_gallus-4.0. We find element distribution is significantly correlated with chromosome size (*r* = 0.72, *P* < 0.001), although less positively than in the Gallus_gallus-4.0 assembly (*r* = 0.91, *P* < 0.001), suggesting that the more complete microchromosomes now include a higher proportion of their repetitive sequences. Overall, 37.8% of intact LTR retrotransposons, unrelated by insertion age or genera, were identified within clusters, where element density was five times that of the genome-wide level. Clusters were invariably found in regions of the genome known to show low recombination rate (International Chicken Genome Sequencing Consortium 2004). Pertinently, element density is 64% higher on GGAZ than would be expected on an autosome of the same length.

### Genome assembly accuracy

**Figure 1:**
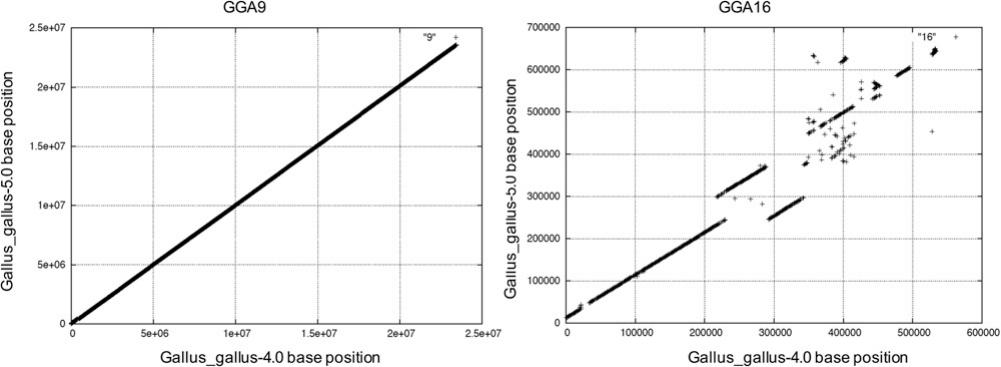
Chromosome synteny alignments of GGA9 and GGA16 derived from Gallus_gallus-4.0 and Gallus_gallus-5.0. Each assembly chromosome file was aligned by BLAT to the other in 1 kb blocks, then parsed by base location.

In general, we observe a high level of sequence assembly synteny in the alignment of the current and previous assembled versions, with few chromosomes exhibiting discrepancies (File S2). We provide one example of near complete synteny, GGA9, and one of remaining discordance, GGA16 (Figure 1). Moreover, alignment of Gallus_gallus-5.0 chromosomes to the chicken genetic linkage map (Cheng *et al.* 1995) reveals few discrepancies (File S3).

To measure base level accuracy, we reviewed single nucleotide substitutions and indels utilizing 36× coverage of aligned data (bird #256; accession no. SRR3954707) to all Gallus_gallus-5.0 placed chromosomes. We called 573,791 high confidence SNPs and of these, we found 15,083 are homozygous allelic differences, a frequency of 5.68 × 10^−4^ genome-wide *vs.* 5.96 × 10^-4^ in Gallus_gallus-4.0, suggesting a very low level of assembly single base consensus error remains after error correction. Given that we used these same reads to correct base error, we also aligned 16.7× coverage of sequences from another bird, Roslin J_line_561 1035N0001 (accession no. ENA PRJEB15276), to call SNPs. We observed 4.25 × 10^−3^ and 4.17 × 10^−3^ SNPs normalized per assembled genome base size for Gallus_gallus-5.0 and Gallus_gallus-4.0, respectively. In a similar experiment (#256 bird; accession no. SRR3954707), we detect normalized high confidence SNP frequencies of 2.26 × 10^−3^ and 5.8 × 10^−4^ for the Gallus_gallus-4.0 and Gallus_gallus-5.0 assemblies for GGAW, respectively. Given that all GGAW SNPs should be base calling errors since GGAW is in a haploid state, we estimate a 3.89-fold higher error rate in Gallus_gallus-4.0; again, not unexpected given we base error-corrected GGAW in only Gallus_gallus-5.0. We also observed a 5.3-fold higher rate of putative false indels within the Gallus_gallus-4.0 version of GGAW. We do concede better assembled repeats in GGAW of Gallus_gallus-5.0 may also reduce misalignments and lower base calling error. Overall, we found that much of the higher estimated base calling error in Gallus_gallus-4.0 is due to various factors, but predominately lack of base error correction with Illumina data and better assembled repeats in Gallus_gallus-5.0.

To measure the overall level of assembly-genome concordance, we aligned BES from the CHORI-261 library to the Gallus_gallus-5.0 assembly, including the scaffolds with no order or orientation but placement on a chromosome, and scaffolds with no assignment. Our expectation was few erroneous alignments would be found, given we utilized this same data to scaffold our original draft assembly, but other postassembly manipulation steps mandated we validate this assumption. We found 89.0% (*n* = 42,316) of clones with both end sequences were placed and 98% of placed clones had a unique concordant placement (*n* = 41,419), indicating the assembly is largely concordant.

### Gene annotation

**Figure 2:**
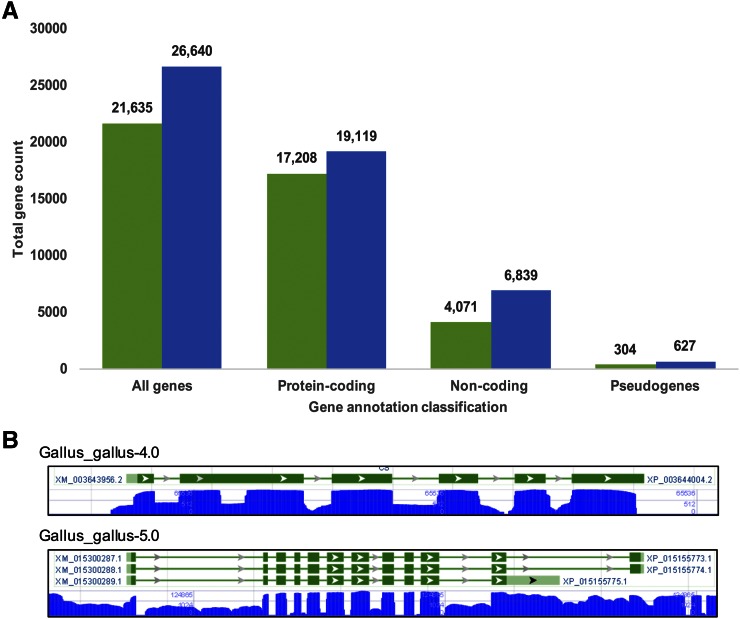
A summary of gene representation within each assembled version. (A) Gene counts derived from the NCBI RefSeq database are parsed by defined gene categories for each assembled version of the chicken genome. Green and blue bars are Gallus_gallus-4.0 and Gallus_gallus-5.0, respectively. (B) Gene model build comparison of the citrate synthase gene (gene ID 100858903) in each assembled version of the chicken genome.

The Gallus_gallus-5.0 assembly was annotated using the NCBI Eukaryotic Annotation Pipeline. The resulting annotation was generated using, among other types of evidence, >9.2 billion RNA sequences from 124 different tissue samples (a listing of tissue sources is found in the NCBI annotation report here: http://www.ncbi.nlm.nih.gov/genome/annotation_euk/Gallus_gallus/103/). The final gene set contains 26,640 genes (including noncoding genes and pseudogenes), 249,174 exons, and 215,764 introns (Figure 2A). Analysis of the changes in gene annotation between Gallus_gallus-4.0 (NCBI annotation release 102) and Gallus_gallus-5.0 (NCBI annotation release 103) shows significant improvement. Of a total of 19,119 protein-coding genes in annotation release 103, 15,260 genes are shared with annotation release 102, and 3859 are unique by definition (Figure 2A). However, there are some count summary differences due to annotation redundancy, mostly the result of immunoglobulin regions or pseudogenes. Upon filtering for annotation duplicates, we derived a set of 3800 unique genes for further analysis. For noncoding RNAs, we see 2768 new transcripts emerge in the Gallus_gallus-5.0 reference (Figure 2A).

Among the 3800 novel protein-coding genes annotated on Gallus_gallus-5.0, 980 genes (1357 proteins) are mapped to a location on Gallus_gallus-4.0 (Current-novel) and therefore were overlooked in annotation release 102; 2129 genes (1357 proteins) are in novel (new sequence) regions of Gallus_gallus-5.0 (Current-unmapped), implying that the sequence added to Gallus_gallus-5.0 helped gene discovery; 350 (478 proteins) cannot be mapped unambiguously to a location on Gallus_gallus-4.0 (Current-other); and there are 361 genes (528 proteins) in other categories, *e.g.*, merged and split genes or changed locus (Table S2). This later set is not considered completely novel. In total, 3304 (75.2%) of the novel proteins were annotated with at least one conserved profile, adding up to 1705 unique InterProScan models. The immunoglobulin-like fold is the most common conserved domain among all categories of novel genes, with 223 proteins in total (File S4). InterProScan (Mitchell *et al.* 2015) was also able to assign 615 unique GO annotation terms to 2170 proteins based on functional domain conservation. In total, 2665 (60%) novel proteins were assigned at least one GO term, and 2275 unique terms were identified (File S5, File S6, File S7, and File S8).

Not only were more genes predicted in Gallus_gallus-5.0, the structure of many genes was improved. The CS citrate synthase gene is an example where in Gallus_gallus-4.0 we annotated one RefSeq transcript with six exons and 5′ and 3′ sequence gaps, while a total of three RefSeq transcripts with 12 exons and no sequence gaps are presented in Gallus_gallus-5.0 (Figure 2B). The citrate synthase gene is localized to GGA33 in Gallus_gallus-5.0, but was unplaced in Gallus_gallus-4.0.

To estimate how much transcript sequence is still missing in Gallus_gallus-5.0, we examined the alignment of chicken RefSeq genes (*n* = 6994). This gene set is gathered from the protein-coding and noncoding known RefSeqs (*e.g.*, with curation support) that were also used during the full gene annotation run. Alignment statistics are available (http://www.ncbi.nlm.nih.gov/genome/annotation_euk/Gallus_gallus/103/#AlignmentStats). In total, we found only 0.19% have no alignment; 0.77% have an alignment that spans more than one scaffold, suggesting that the corresponding gene is split; and 1.66% have an alignment that covers <95% of the coding sequence, an indication of incompleteness.

We also re-examined a set of 571 genes that were previously found to be missing in the chicken genome (Lovell *et al.* 2014) (in Lovell *et al.* 2014 see Table S1 and Table S6, plus select entries in Table S4 and Table S18). In parallel, we also conducted Entrez (Brown *et al.* 2015) gene and BLAST searches of Gallus_gallus-5.0 for an additional set of 143 genes (see select entries within Table S4 and Table S18 in Lovell *et al.* 2014) that were previously found to be partial and highly truncated, or that were only present on unplaced segments in Gallus_gallus-4.0.

Our analyses show that: (a) a subset of 232 genes are still absent in Gallus_gallus-5.0 and also not present in any of the >60 avian genomes currently in NCBI (Table S3); (b) a subset of 129 genes are still absent in Gallus_gallus-5.0, even though they have been found in other bird species (Table S4); (c) a subset of 240 genes that were not present in Gallus_gallus-4.0 are now present in Gallus_gallus-5.0 (Table S5), with associated new gene model predictions; and (d) yet another subset of 111 that had already been found in Gallus_gallus-4.0 are now better represented in Gallus_gallus-5.0 (Table S6), in many cases with longer sequences, predictive models, and/or chromosome placement/localization. Categories (c) and (d) are consistent with the broader analysis presented in previous sections, providing further evidence that Gallus_gallus-5.0 is a substantially improved assembly. In contrast, category (a) provides additional confirmatory evidence of protein gene losses specific to birds, whereas category (b) provides evidence for possible additional gene losses in chicken. Of note, the existing predictions for several of these genes are misannotated and actually represent paralogs (Table S3). Interestingly, a subset of these genes are orthologous to genes in regions of human chromosomes 19, 14, 12, and X which, based on other synteny, are likely to map to Gallus_gallus-5.0 microchromosomes (and tend to be orthologous to unplaced lizard scaffolds), suggesting that these elements of the chicken genome correspond to regions that may be difficult to sequence/assemble/map and/or that may have undergone substantial changes (rearrangements, sequence divergence) across these vertebrate lineages.

### Immune system genes: MHC genes on GGA16

**Figure 3:**
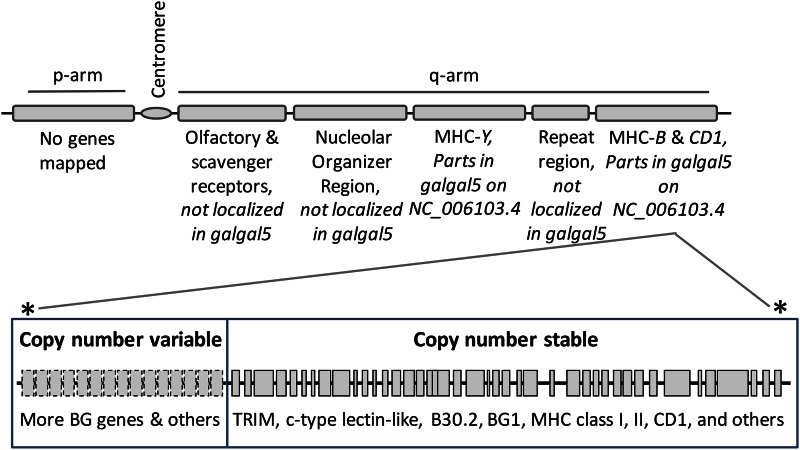
Map for chicken chromosome 16 (GGA16) p- and q-arms. Genes currently mapped to GGA16 are described in four regions: (1) the olfactory and scavenger receptor gene region, (2) the nucleolar organizer region containing ribosomal RNA genes, (3) the MHC-*Y* region containing mainly MHC class I-like and c-type lectin-like genes, and (4) the MHC-*B* and *CD1* gene region. Intervening between MHC-*Y* and the MHC-*B* and *CD1* region is a region known to contain repeat sequences. Currently available haplotype sequences suggest that in one portion of the MHC-*B* and *CD1* region the gene number is stable and in the other the copy number is variable. Asterisk indicates that whether the copy number variable subregion is proximal (as drawn) or the copy number stable region of MHC-*B* and *CD1* is proximal remains to be determined.

The chicken MHC on GGA16 has long been known as a gene region contributing significantly in genetic resistance to infectious diseases. For Gallus_gallus-5.0, we aligned the assembled regions of GGA16 to a previously characterized MHC-*B* sequence (MHC AB268588.1) that is derived from finished CHORI-261 BACs (Shiina *et al.* 2007), and then built an assembly overview of this critical region (Figure 3). Overall, we observe more sequence content, fewer gaps, single base substitutions, and indels reflecting higher base accuracy in this region (Figure S1). In one example, we found a 61-kb insertion in Gallus_gallus-4.0 compared with MHC AB268588.1, which we believe represents misplaced contigs since Gallus_gallus-5.0 and MHC AB268588.1 agree at this location. Utilizing this new MHC region of Gallus_gallus-5.0, we confirm significant SNP variability across MHC-*B*, supporting findings from an earlier extensive study of MHC haplotypes (Fulton *et al.* 2016). Gallus_gallus-5.0 also contains 1.5-fold more sequence identifiable as representative of the second MHC region on GGA16, called MHC-*Y*. MHC-*Y* contains distinctive MHC-like class I genes, similar to MR1 and class II genes, along with a number of c-type lectin-like genes (Miller and Taylor 2016). Although located on GGA16, MHC-*Y* is separated from MHC-*B* by a high frequency of recombination region and, as a result, MHC-*Y* haplotypes assort independently of MHC-*B* haplotypes (Figure 3). When both assembled versions are separately aligned with a previously assembled region of MHC-*Y*, we observed greater sequence content (identity increased nearly sixfold) and fewer gaps (0.025% *vs.* 0.19%) in Gallus_gallus-5.0.

### Immune system genes: CHIR receptors on GGA31

Immunoglobin-like receptor (CHIR) regions are important to study because of how they contribute to host response to the challenge of pathogens. In mammals, the leukocyte receptor complex (LRC) codes for immune-related cell surface receptors that regulate T and B lymphocytes, and natural killer cells. The chicken equivalent to LRC are the chicken CHIR loci (Laun *et al.* 2006). Many and possibly all CHIR are thought to be linked and have been assigned tentatively to GGA31 by FISH (Viertlboeck *et al.* 2005). Gallus_gallus-5.0 contains a GGA31 scaffold with 49,161 bp represented. Including an additional 11 unordered scaffolds lacking orientation, a total of 119,703 bp have been assigned to GGA31. Among the CHIR sequences previously assembled, at least 84% mapped to GGA31 in Gallus_gallus-5.0. Thus, while GGA31 may not be completely assembled in Gallus_gallus-5.0, the CHIR loci sequences are well represented.

### Linkage mapping of unplaced sequences

Genetic maps form the foundation for the chicken genome assembly. To improve the genetic map, 9585 of unassigned Gallus_gallus-5.0 sequence contigs 2 kb or larger (99% of the 138 Mb unassigned) were screened for potentially informative polymorphism. By aligning UCD003 (White Leghorn, the other parent of the East Lansing mapping population) sequences, 4160 scaffolds were identified that cover ∼39.6 Mb, with at least one SNP assigned that passed our quality thresholds. Further filtering based on Affymetrix design scores provided 5907 SNPs on 547 contigs that potentially could be genotyped, and of these, 3440 SNPs on 510 contigs could be assayed and successfully scored.

Following genetic mapping, 3437 SNPs were assigned to 29 new linkage groups (referenced as E101–E129; see File S9 for linkage maps), none of which were linked to existing markers among the known reference linkage groups associated with existing chromosomes (Table S7). Three SNPs were unlinked and assigned to E00. In the future, it is possible these linkage groups could aid efforts to validate “missing” microchromosome locations by traditional FISH methods, thereby better integrating the sequence assembly with the chicken karyotype. We provide a summary of linkage marker data should other groups need to utilize this information for future studies (File S10). However, at this time it is expected evolving *de novo* assembly algorithms, longer read input (*i.e.*, 15 kb), and higher resolution chromatin mapping techniques will resolve these gaps in connectivity and missing microchromosome assignments.

## Discussion

Higher quality genome assemblies are becoming increasingly necessary to achieve the full potential of next generation sequencing studies, as initial “draft” assemblies have been found insufficient for the more complete discovery of allelic contributions to particularly complex traits. Given the already substantial progress in avian trait mapping, coupled with the availability of genomic resources, there is continued motivation to improve the quality of the chicken reference genome (Reyer *et al.* 2015; Rubin *et al.* 2010). A key objective of this study was to improve genome representation in all ways possible. In this latest iteration of the chicken reference genome assembly, we add new sequences, improve sequence connectivity, identify errors, and provide a substantially improved gene set for future research. Compared to Gallus_gallus-4.0, the Gallus_gallus-5.0 assembly added a total of 183 Mb new sequences, showed a 10-fold increase in contig N50 length, and added 4679 annotated genes, both protein-coding and noncoding transcripts. However, and not unexpectedly, our assessment of the retrotransposon distribution genome-wide suggests these elements, and likely other repeat types, may be major hurdles in assembling the remaining missing sequences, particularly microchromosomes. Nonetheless, annotated repeat content increased across all repeat classes present in the chicken genome. This included annotation of 30,496 more copies of CR1—the most numerous chicken repetitive element. Total LTR retrotransposon content increased by 3.3 Mbp in Gallus_gallus-5.0, despite the annotated percentage falling due to the increased assembly length. Despite a modest improvement in our ability to detect these repeats in Gallus_gallus-5.0, there remain regions highly enriched with fragmented and intact LTR retrotransposons in both unplaced and chromosome-aligned sequences (*e.g.*, GGA16 and GGAW). Similar observations were made for GGA30, GGA31, and GGA33, newly available in Gallus_gallus-5.0.

Another important goal was to improve assembly base accuracy throughout this reference. Our results clearly demonstrate that overall base accuracy is higher in the Gallus_gallus-5.0 assembly as a result of the Illumina error correction process, deeper sequence coverage, and the sequence technology composition of the Gallus_gallus-4.0 assembly, which is a hybrid assembly of 454 pyrosequences (∼12×) and Sanger sequencing (∼6×).

Our higher level of sequence representation in the chicken genome, diverse tissue RNA-Seq data, and improved gene finding algorithms all contributed to the increased gene count observed in Gallus_gallus-5.0. We used this new gene catalog to continue efforts aimed at defining genes that are uniquely missing in the avian lineage and thus represent a possible means to develop new comparative models for study of biomedical relevance. A large fraction of our original set of missing genes of high confidence still remain undiscovered in Gallus_gallus-5.0, thus, the chicken could potentially serve as a naturally evolved model to study the physiological consequence of these gene losses. These missing chicken genes are present and largely conserved in most other vertebrate lineages, including reptiles (lizard, turtle, and crocodile), and are organized into syntenic clusters that mostly map onto human chromosomes 19, 14, X, and 12, or are located in very close proximity to these clusters (Lovell *et al.* 2014). Future efforts to reannotate the current collection of avian genomes and many others currently underway will benefit from these and other advances (Zhang *et al.* 2015). Nonetheless, in chicken and other species, many genes still have no assigned function and can only be classified through experimentation and manual curation—both costly approaches.

A variety of immunologically interesting genes have been mapped to GGA16, including genes in MHC-*B*, MHC-*Y*, *SRCR*, and *OR* (Miller and Taylor 2016). Given the highly polymorphic nature of the region (Fulton *et al.* 2016) and the known immune function of many of the genes, accurate and complete genome assembly is important for continued studies of immunity in avian diseases. Assembly of GGA16 has advanced in Gallus_gallus-5.0 and is now supporting the emerging picture that some segments of GGA16 are subject to gene copy number variation (Afanassieff *et al.* 2001; Salomonsen *et al.* 2014; Fulton *et al.* 2016). However, more complete MHC and other immune system genes sequence representation are needed to further advance our knowledge of immune response. For example, the immunological importance of the CHIR loci has made them major research targets. CHIR regions were previously localized to GGA31 but our new results suggest that Gallus_gallus-5.0 contains a more complete portion of the transcribed region of the CHIR cluster. However, we do observe CHIR locus orientation discrepancies, such as CHIR transcript split mappings, that will necessitate changes to future assembled versions of this region.

Many phenotypes that are of interest to poultry breeders, such as disease resistance, growth, and reproduction, are, in other species, regulated by genes that are members of large gene families. It is well known that these rapidly evolving, dynamic regions are often collapsed or even absent from draft genome assemblies (Chaisson *et al.* 2014). These observations imply that a significant amount of avian phenotypic variation is likely to be modulated by genes or gene families imbedded within repetitive structures. While the current Gallus_gallus-5.0 assembly is a major step forward toward generating a more complete reference, the future generation of data from evolving long single molecule sequencing chemistries and other emerging scaffolding technologies (*e.g.*, chromatin proximity mapping) will lead to additional improvements in the quality and completeness of the chicken genome (Chaisson *et al.* 2014; Putnam *et al.* 2016).

## Supplementary Material

Supplemental material www.g3journal.org/lookup/suppl/doi:10.1534/g3.116.035923/-/DC1.
