# Supplementary material for: A New Chicken Genome Assembly Provides Insight into Avian Genome Structure
Source: G3 (Bethesda). 2016 Nov 14;7(1):109–17. doi: 10.1534/g3.116.035923 (PMC5217101; doi:10.1534/g3.116.035923)
Supplement: Supplementary file 1 [file 109FigureS1.docx]

**Figure S1**. Measured sequence representation and discrepancies in chicken MHC. Alignments of MHC accession AB268588 (an independent gap free assembly of bird #256 based on tiled CHORI-251 BACs) to Gallus_gallus-4.0 and Gallus_gallus-5.0 were performed. In Gallus_gallus-4.0 there are 342 single base mismatches and 137 indels while Gallus_gallus-5.0 shows 200 mismatches and 111 indels. Red bars indicate indel or single base mismatch events. Blue bars represent inserted sequences. Gaps are denoted as no grey bar sequence between within each assembled version.

Gallus_gallus-5.0

Gallus_gallus-4.0

Annotated NCBI genes of Gallus_gallus-5.0

AB268588.1 accessioned sequence
