# Supplementary material for: A New Chicken Genome Assembly Provides Insight into Avian Genome Structure
Source: G3 (Bethesda). 2016 Nov 14;7(1):109–17. doi: 10.1534/g3.116.035923 (PMC5217101; doi:10.1534/g3.116.035923)
Supplement: Supplementary file 3 [file 109FigureS3.docx]

**Figure S3**. Gallus_gallus-5.0 sequence coverage of aligned reference sequences (bird #256; accession SRR3954707). All sequences were aligned to each assembled version of the chicken genome using the standard BWA-MEM aligner. All bases aligned that pass our quality criteria were counted to calculate percentage of bases aligned.
