# Supplementary material for: A New Chicken Genome Assembly Provides Insight into Avian Genome Structure
Source: G3 (Bethesda). 2016 Nov 14;7(1):109–17. doi: 10.1534/g3.116.035923 (PMC5217101; doi:10.1534/g3.116.035923)
Supplement: Supplementary file 4 [file 109FigureS4.docx]

**Figure S4.** Distribution of total GC bases for individual Gallus_gallus-5.0 chromosomes.
