# Supplementary material for: A New Chicken Genome Assembly Provides Insight into Avian Genome Structure
Source: G3 (Bethesda). 2016 Nov 14;7(1):109–17. doi: 10.1534/g3.116.035923 (PMC5217101; doi:10.1534/g3.116.035923)

**Figure S5.** Subread distribution for all long read that served as input for de novo assembly. The green histogram presents subread counts by size. The black plot line represents the size of bases for subread length.


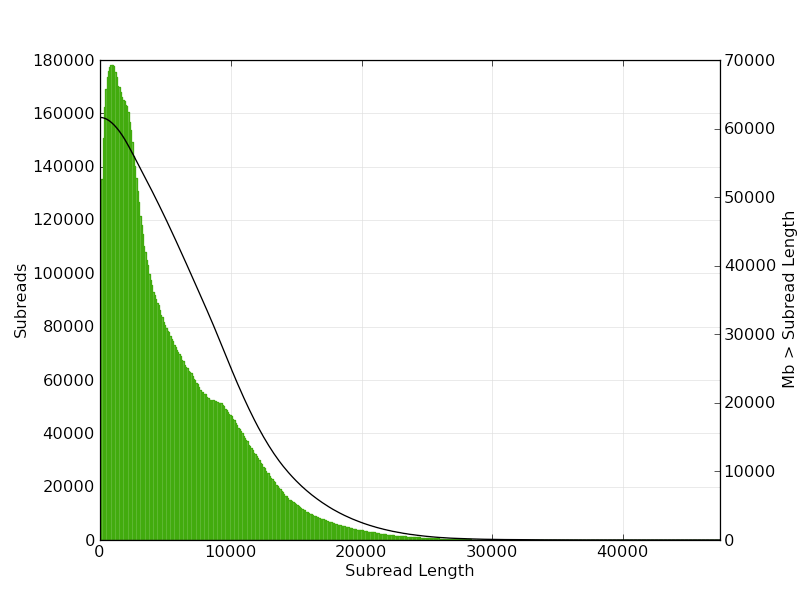

Supplement: Supplementary file 5 [file 109FigureS5.docx]
