# Supplementary material for: A New Chicken Genome Assembly Provides Insight into Avian Genome Structure
Source: G3 (Bethesda). 2016 Nov 14;7(1):109–17. doi: 10.1534/g3.116.035923 (PMC5217101; doi:10.1534/g3.116.035923)
Supplement: Supplementary file 6 [file 109FileS1.docx]

File S1. The predicted protein sequences corresponding to newly predicted coding genes retrieved from the NCBI proteome (<ftp://ftp.ncbi.nih.gov/genomes/Gallus_gallus/protein/protein.fa.gz>) and scanned for conserved Interpro signatures in eleven databases of protein predictive models. (.xls, 4.83 MB)

<http://www.g3journal.org/lookup/suppl/doi:10.1534/g3.116.035923/-/DC1/FileS1.xls>
