# Supplementary material for: A New Chicken Genome Assembly Provides Insight into Avian Genome Structure
Source: G3 (Bethesda). 2016 Nov 14;7(1):109–17. doi: 10.1534/g3.116.035923 (PMC5217101; doi:10.1534/g3.116.035923)
Supplement: Supplementary file 8 [file 109FileS2.pdf]

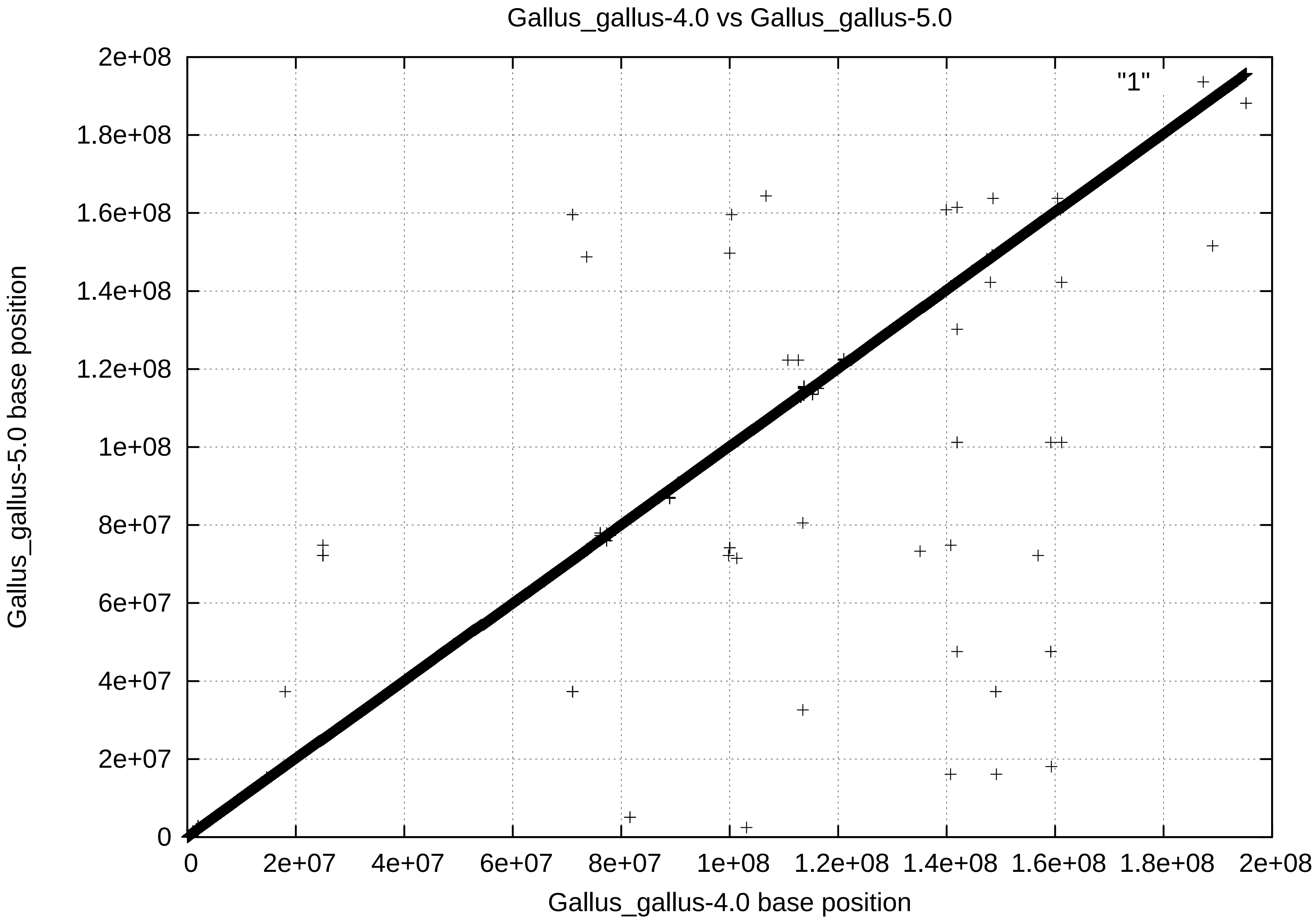
Gallus_gallus-4.0 vs Gallus_gallus-5.0
"1"
2e+08
1.8e+08
1.6e+08
1.4e+08
1.2e+08
1e+08
8e+07
6e+07
4e+07
2e+07
0
Gallus_gallus-5.0 base position
0
2e+07
4e+07
6e+07
8e+07
1e+08
1.2e+08
1.4e+08
1.6e+08
1.8e+08
2e+08
Gallus_gallus-4.0 base position

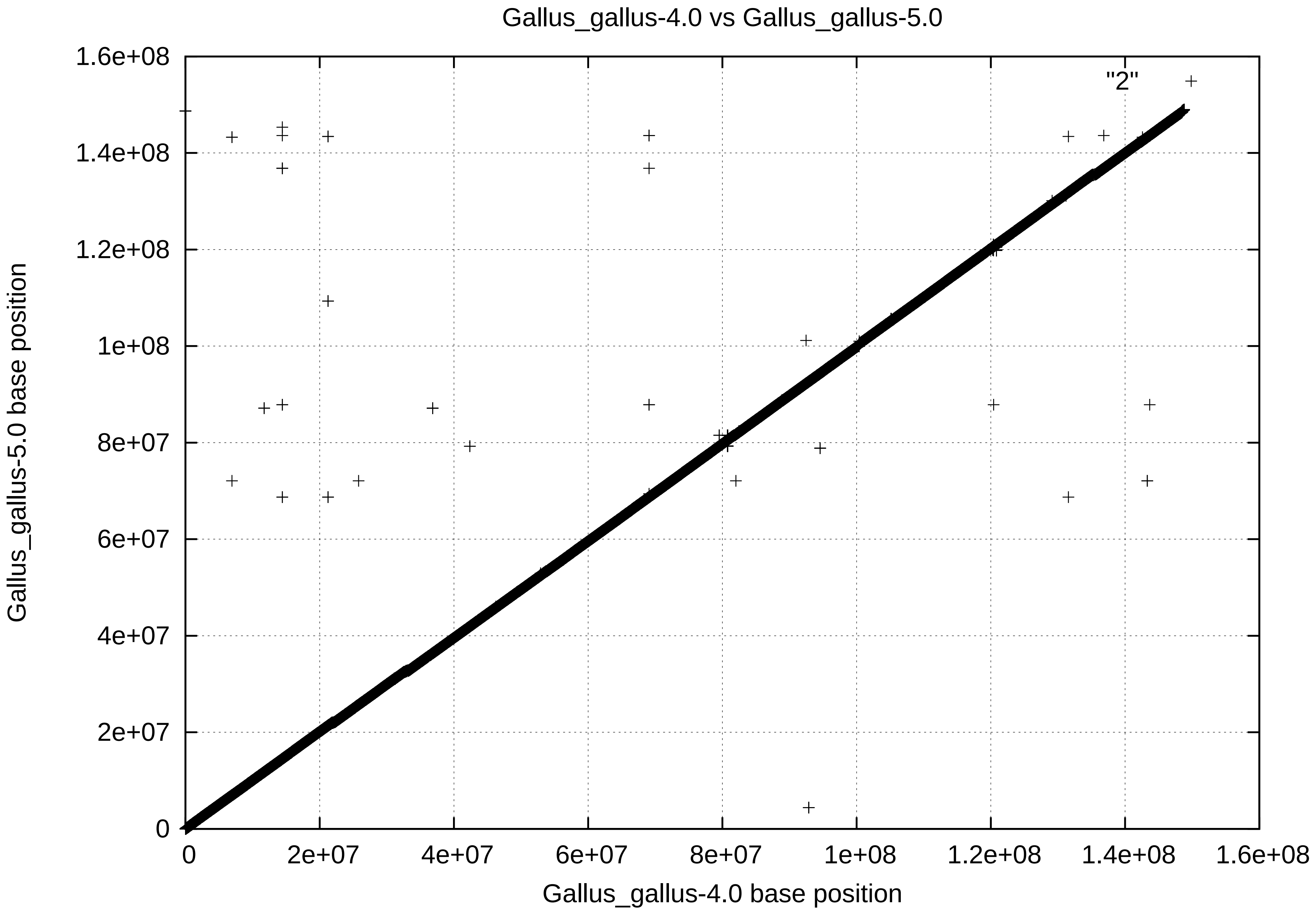
Gallus_gallus-4.0 vs Gallus_gallus-5.0
"2"
Gallus_gallus-5.0 base position
Gallus_gallus-4.0 base position
0
2e+07
4e+07
6e+07
8e+07
1e+08
1.2e+08
1.4e+08
1.6e+08

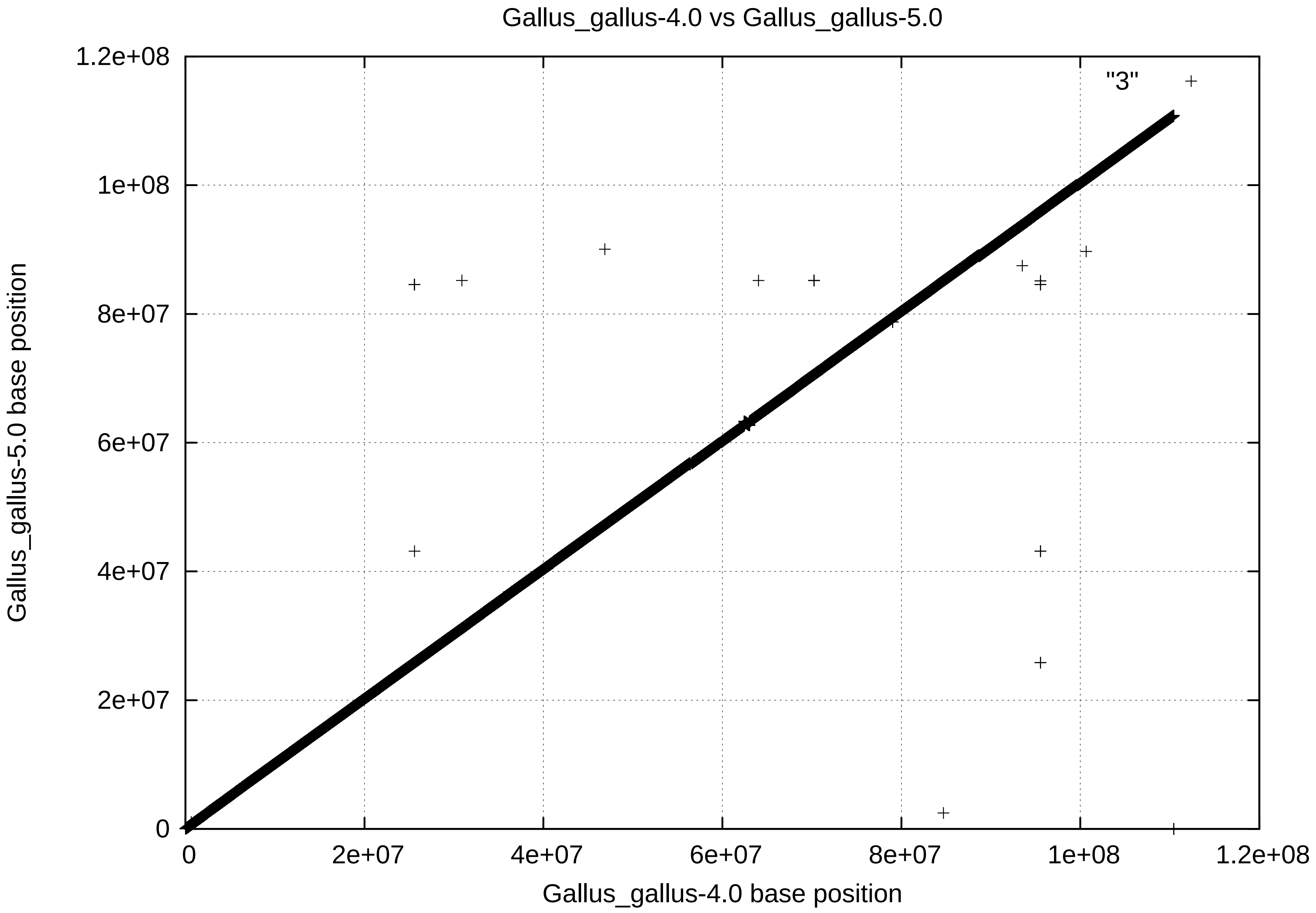

Gallus_gallus-4.0 vs Gallus_gallus-5.0
"3"
Gallus_gallus-5.0 base position
Gallus_gallus-4.0 base position
0
2e+07
4e+07
6e+07
8e+07
1e+08
1.2e+08

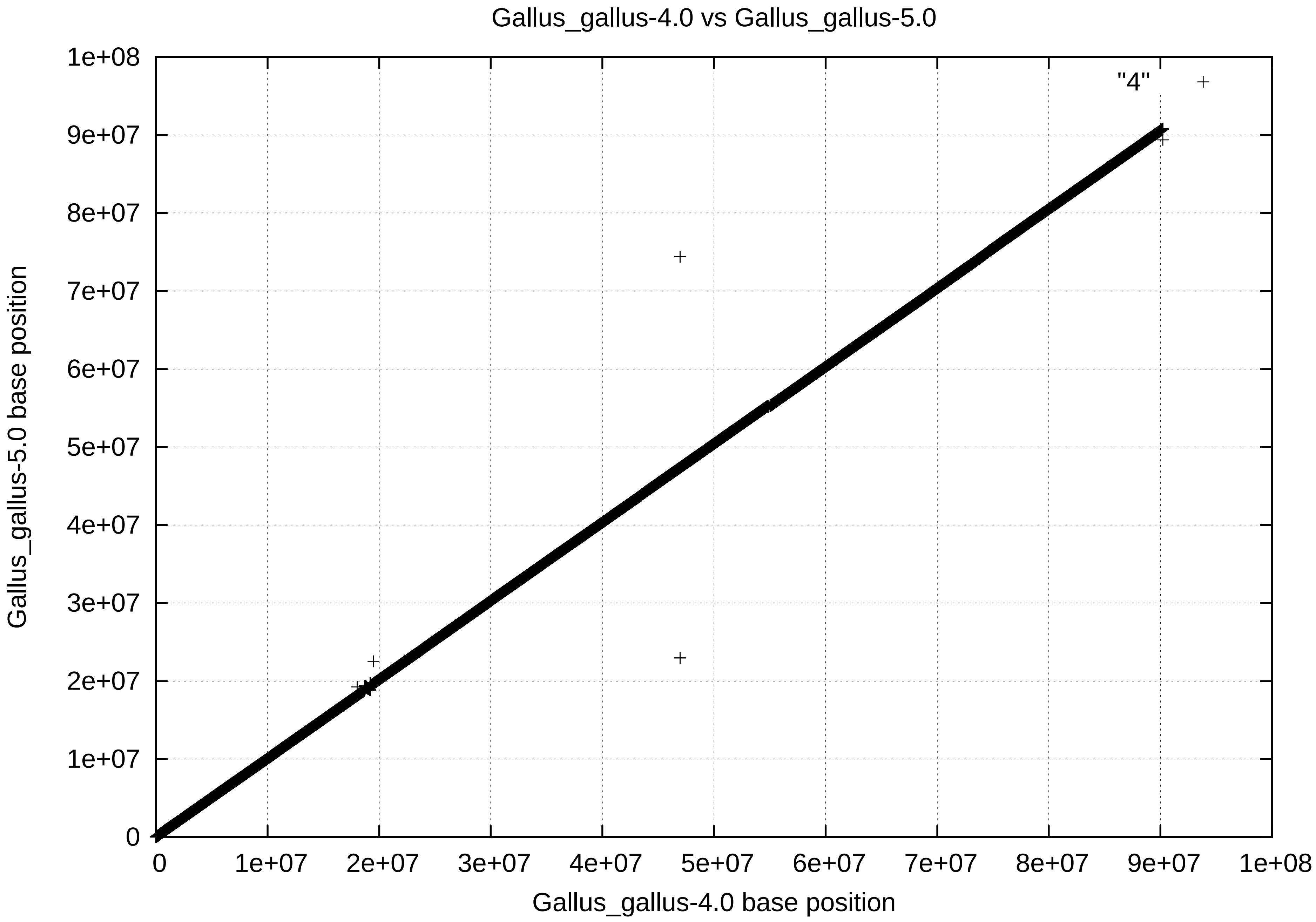

Gallus_gallus-4.0 vs Gallus_gallus-5.0
"4"
Gallus_gallus-5.0 base position
Gallus_gallus-4.0 base position
0
1e+07
2e+07
3e+07
4e+07
5e+07
6e+07
7e+07
8e+07
9e+07
1e+08

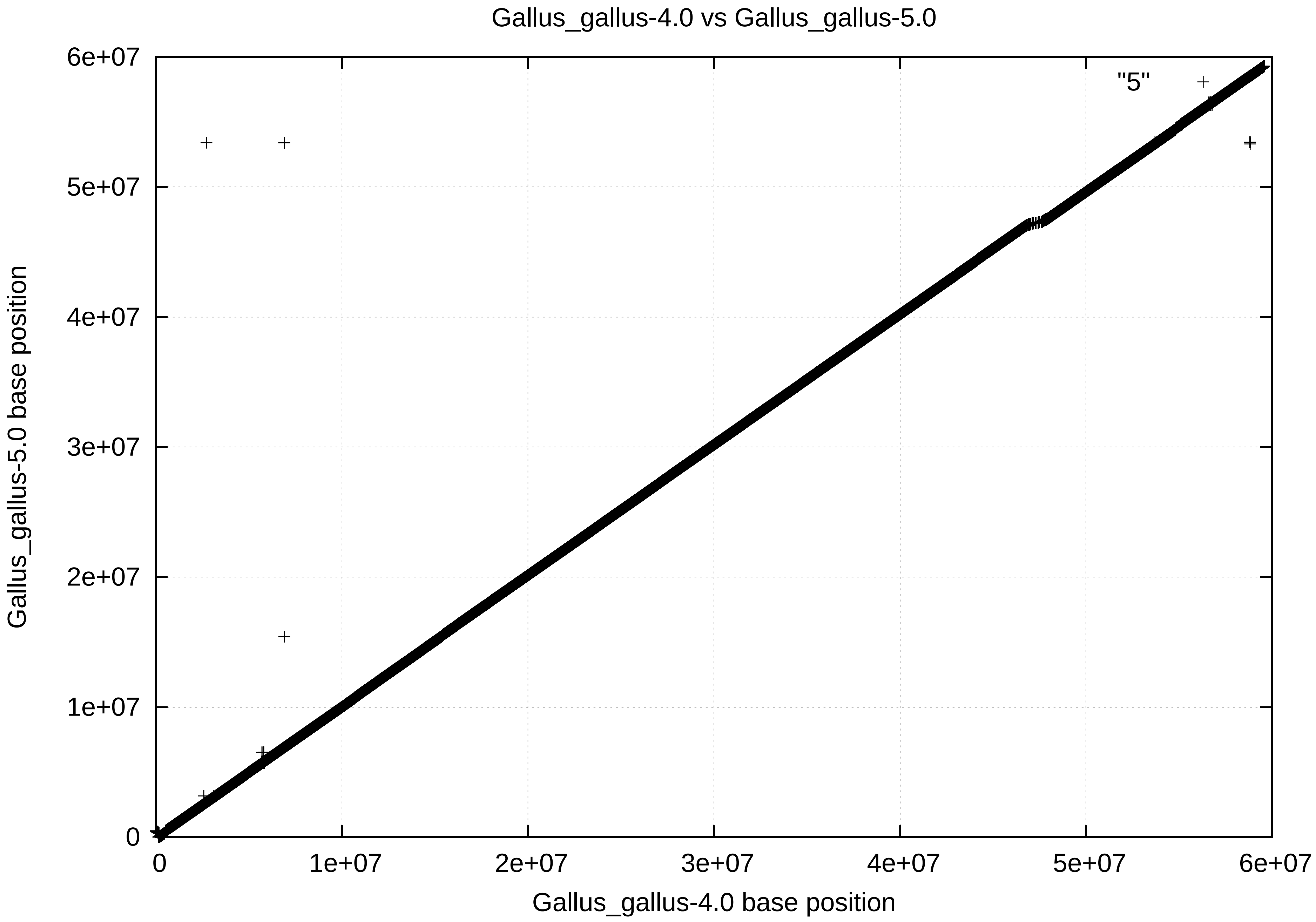

Gallus_gallus-4.0 vs Gallus_gallus-5.0
"5"
Gallus_gallus-5.0 base position
Gallus_gallus-4.0 base position
0
1e+07
2e+07
3e+07
4e+07
5e+07
6e+07

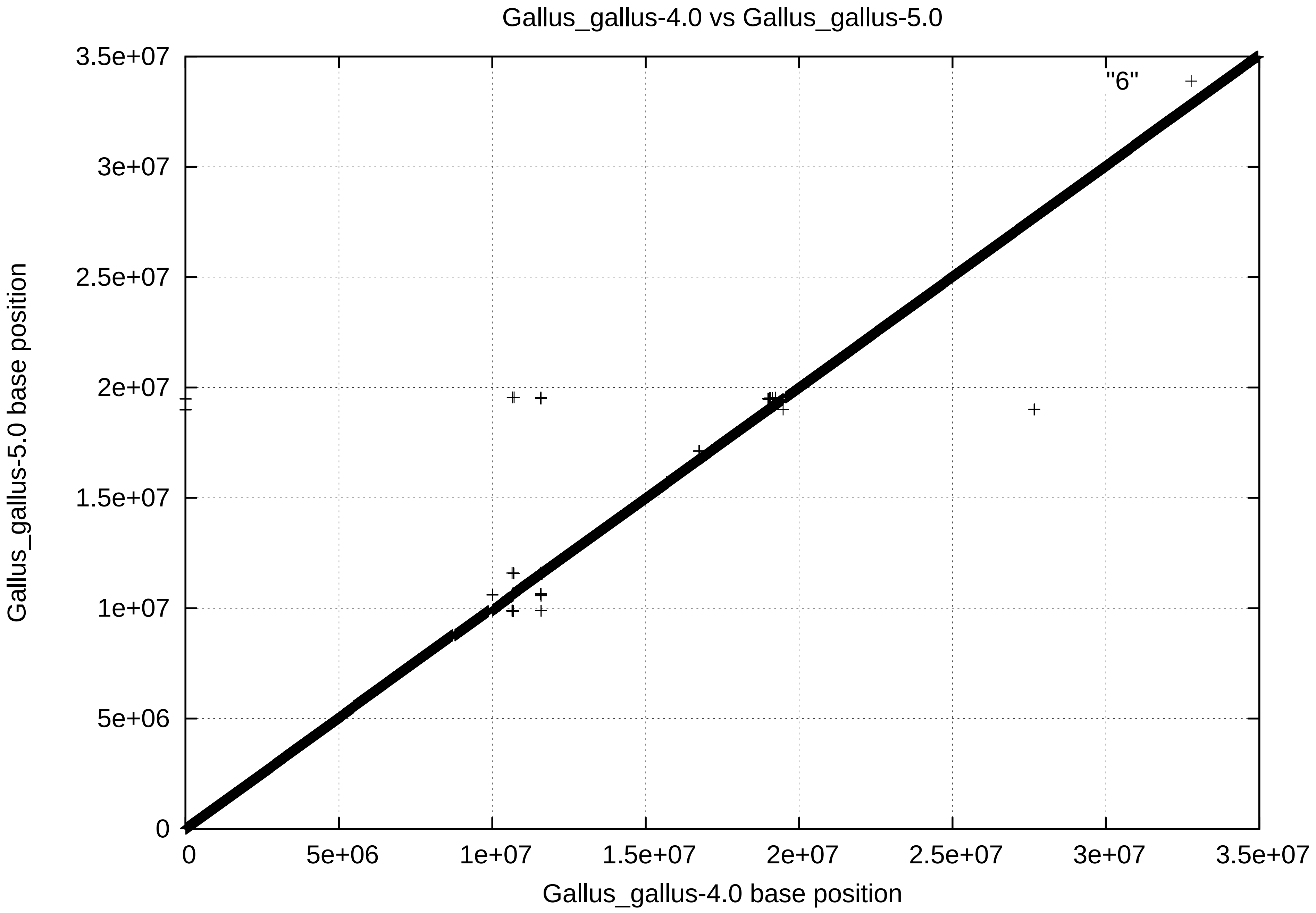

Gallus_gallus-4.0 vs Gallus_gallus-5.0
"6"
3.5e+07
3e+07
2.5e+07
2e+07
1.5e+07
1e+07
5e+06
0
0
5e+06
1e+07
1.5e+07
2e+07
2.5e+07
3e+07
3.5e+07
Gallus_gallus-4.0 base position
Gallus_gallus-5.0 base position

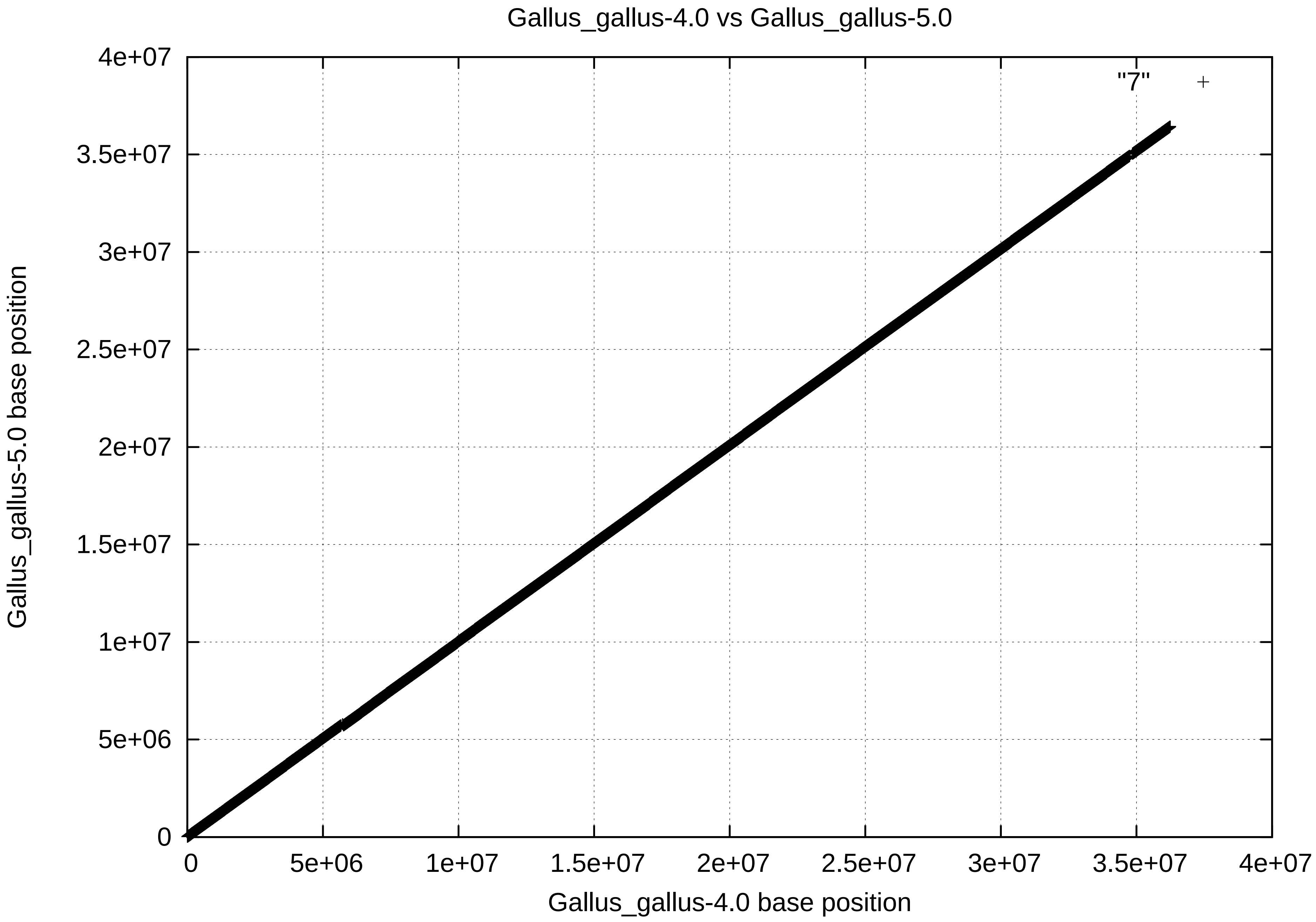

Gallus_gallus-4.0 vs Gallus_gallus-5.0
"7"
4e+07
3.5e+07
3e+07
2.5e+07
2e+07
1.5e+07
1e+07
5e+06
0
0
5e+06
1e+07
1.5e+07
2e+07
2.5e+07
3e+07
3.5e+07
4e+07
Gallus_gallus-4.0 base position
Gallus_gallus-5.0 base position

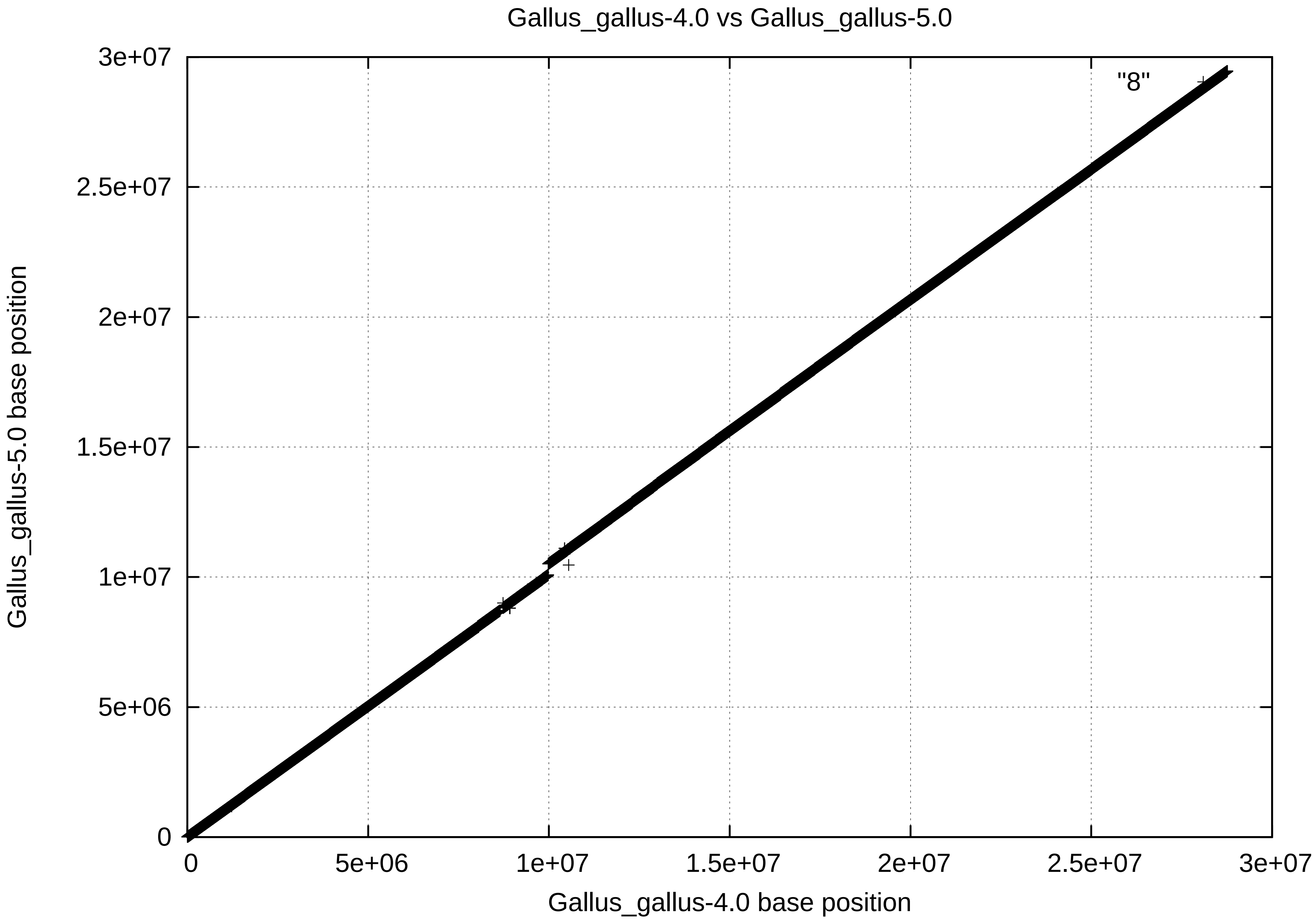

Gallus_gallus-4.0 vs Gallus_gallus-5.0
"8"
3e+07
2.5e+07
2e+07
1.5e+07
1e+07
5e+06
0
0
5e+06
1e+07
1.5e+07
2e+07
2.5e+07
3e+07
Gallus_gallus-4.0 base position
Gallus_gallus-5.0 base position

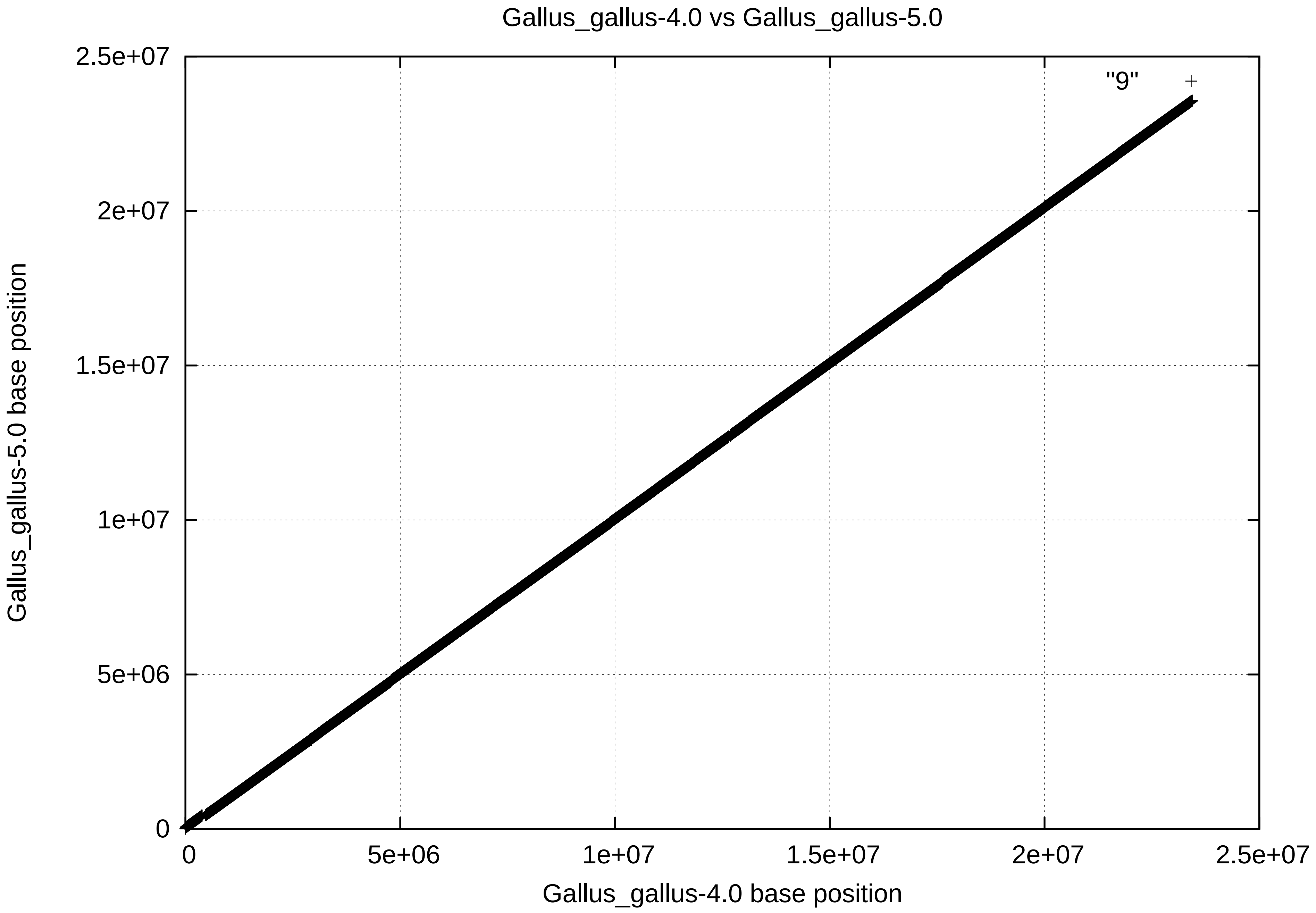

Gallus_gallus-4.0 vs Gallus_gallus-5.0
"9"
2.5e+07
2e+07
1.5e+07
1e+07
5e+06
0
0
5e+06
1e+07
1.5e+07
2e+07
2.5e+07
Gallus_gallus-5.0 base position
Gallus_gallus-4.0 base position

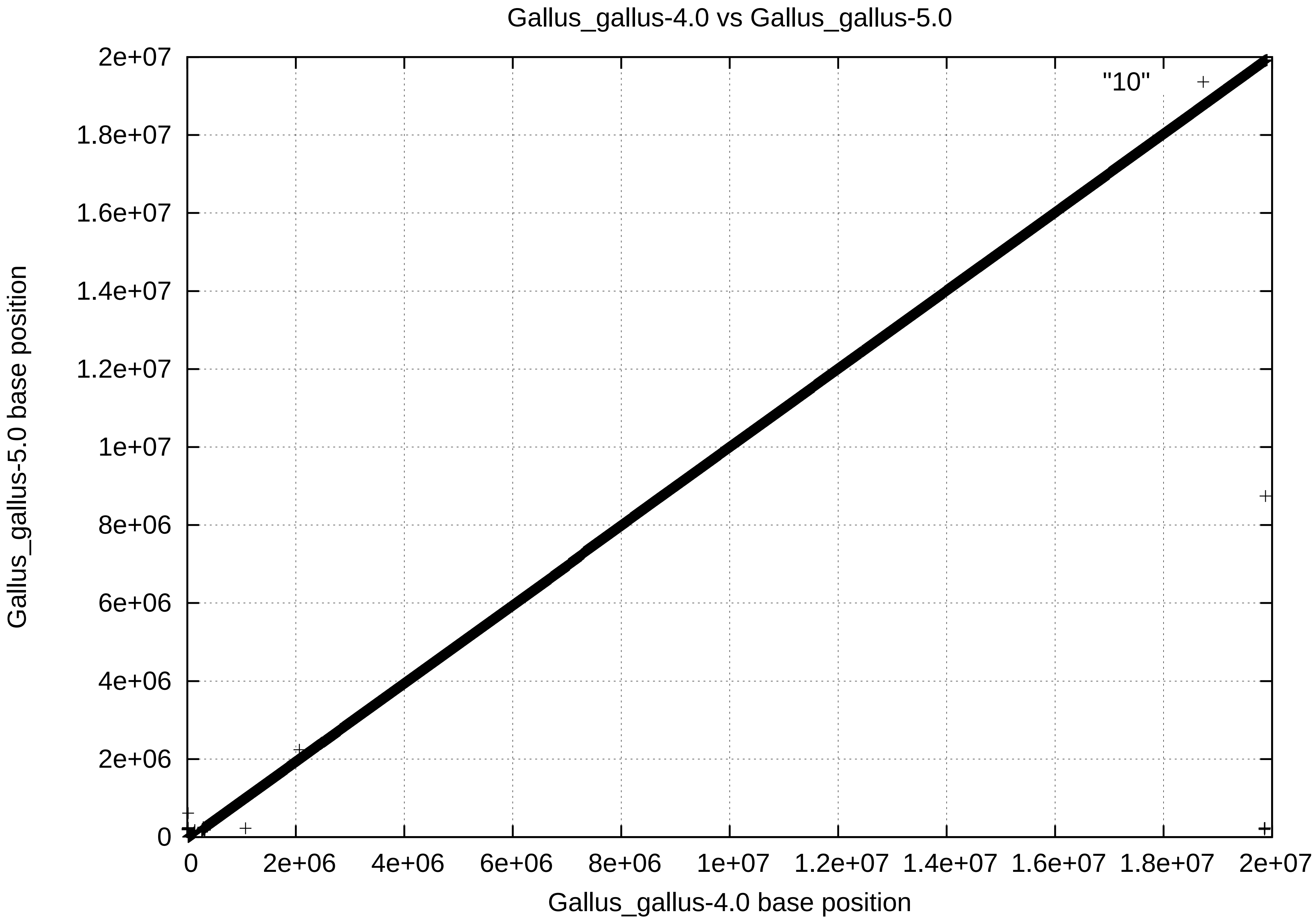
Gallus_gallus-4.0 vs Gallus_gallus-5.0
"10"
Gallus_gallus-5.0 base position
Gallus_gallus-4.0 base position
0
2e+06
4e+06
6e+06
8e+06
1e+07
1.2e+07
1.4e+07
1.6e+07
1.8e+07
2e+07

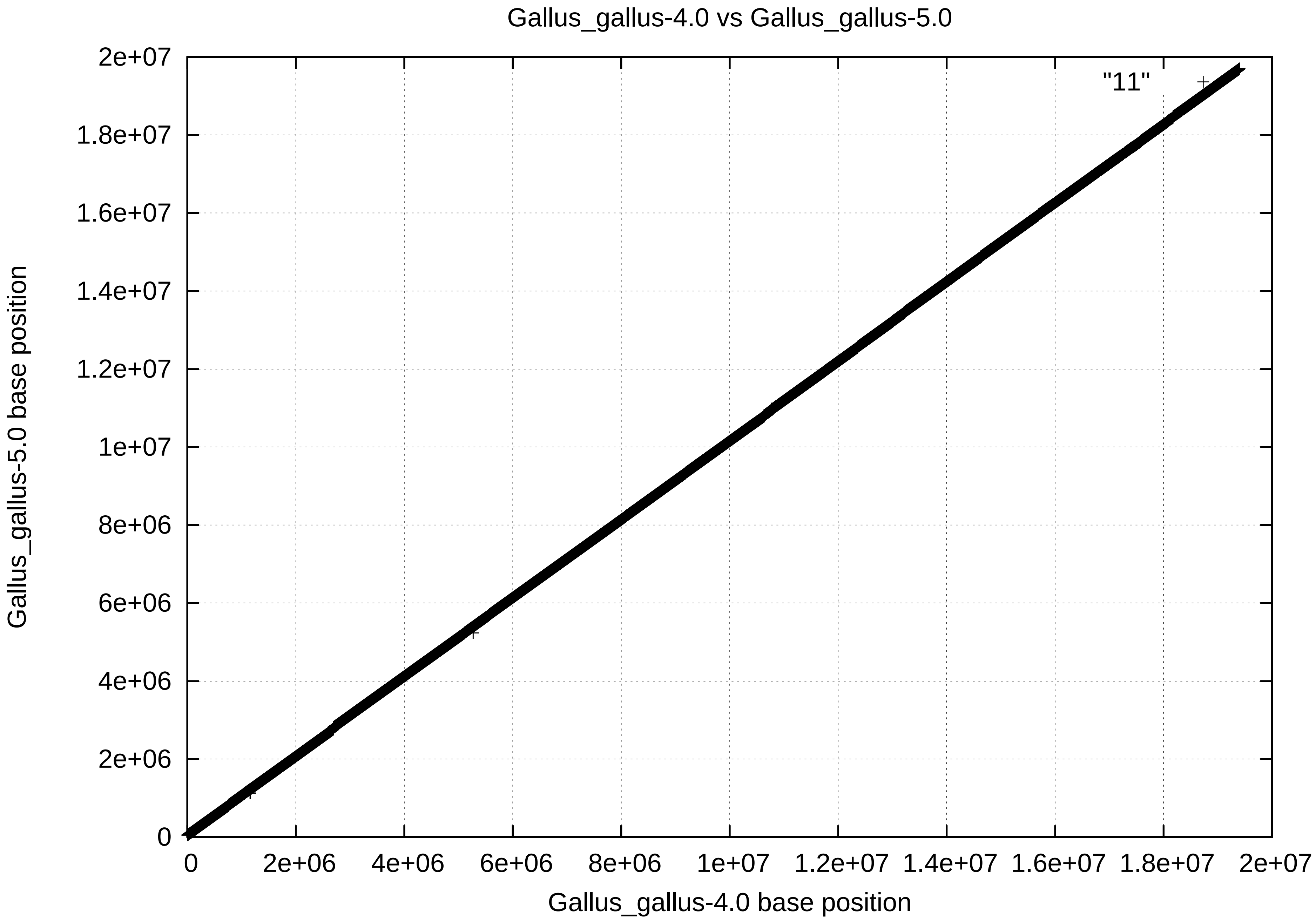

Gallus_gallus-4.0 vs Gallus_gallus-5.0
"11"
Gallus_gallus-5.0 base position
Gallus_gallus-4.0 base position
0
2e+06
4e+06
6e+06
8e+06
1e+07
1.2e+07
1.4e+07
1.6e+07
1.8e+07
2e+07

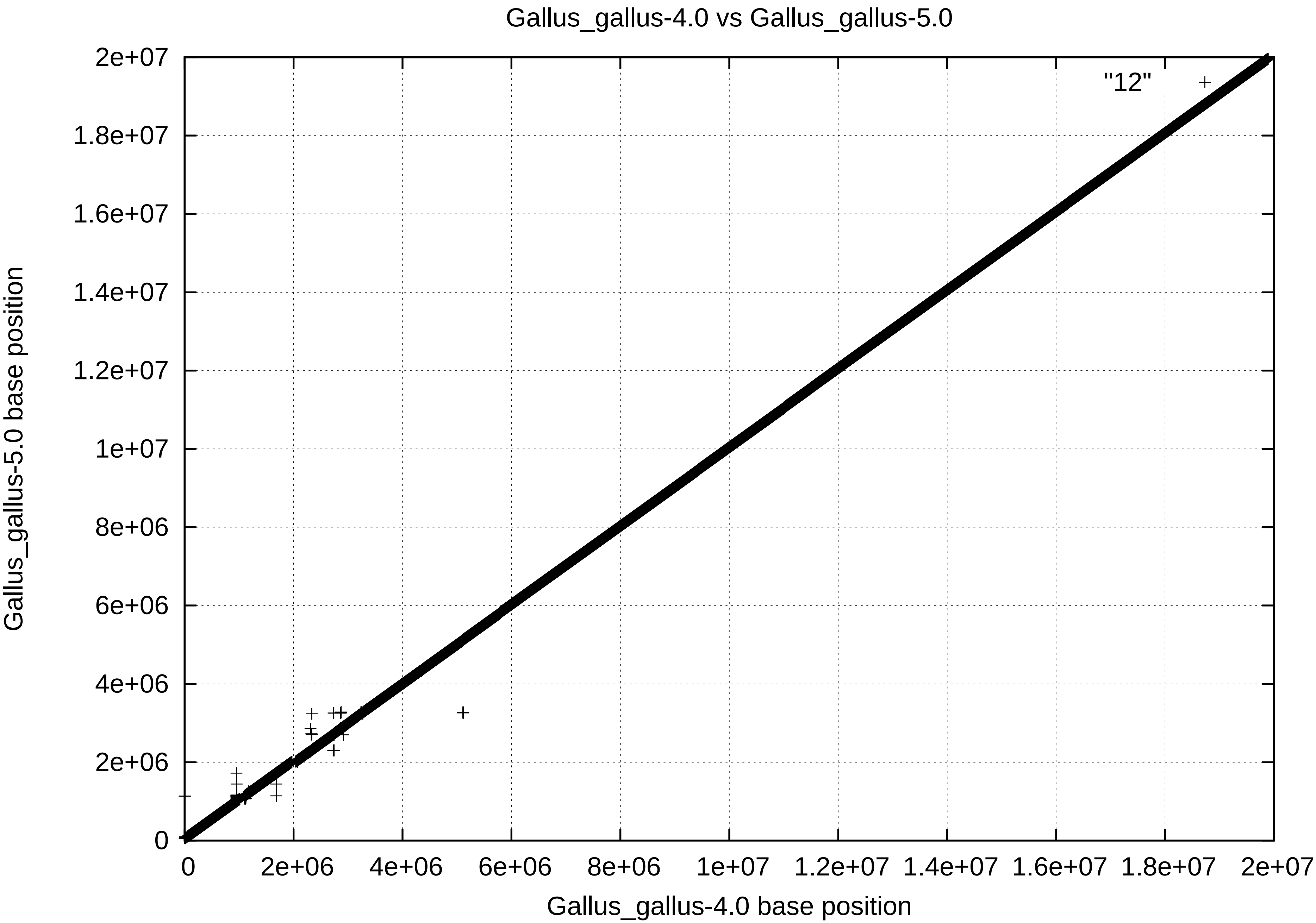

Gallus_gallus-4.0 vs Gallus_gallus-5.0
"12"
Gallus_gallus-5.0 base position
Gallus_gallus-4.0 base position
0
2e+06
4e+06
6e+06
8e+06
1e+07
1.2e+07
1.4e+07
1.6e+07
1.8e+07
2e+07

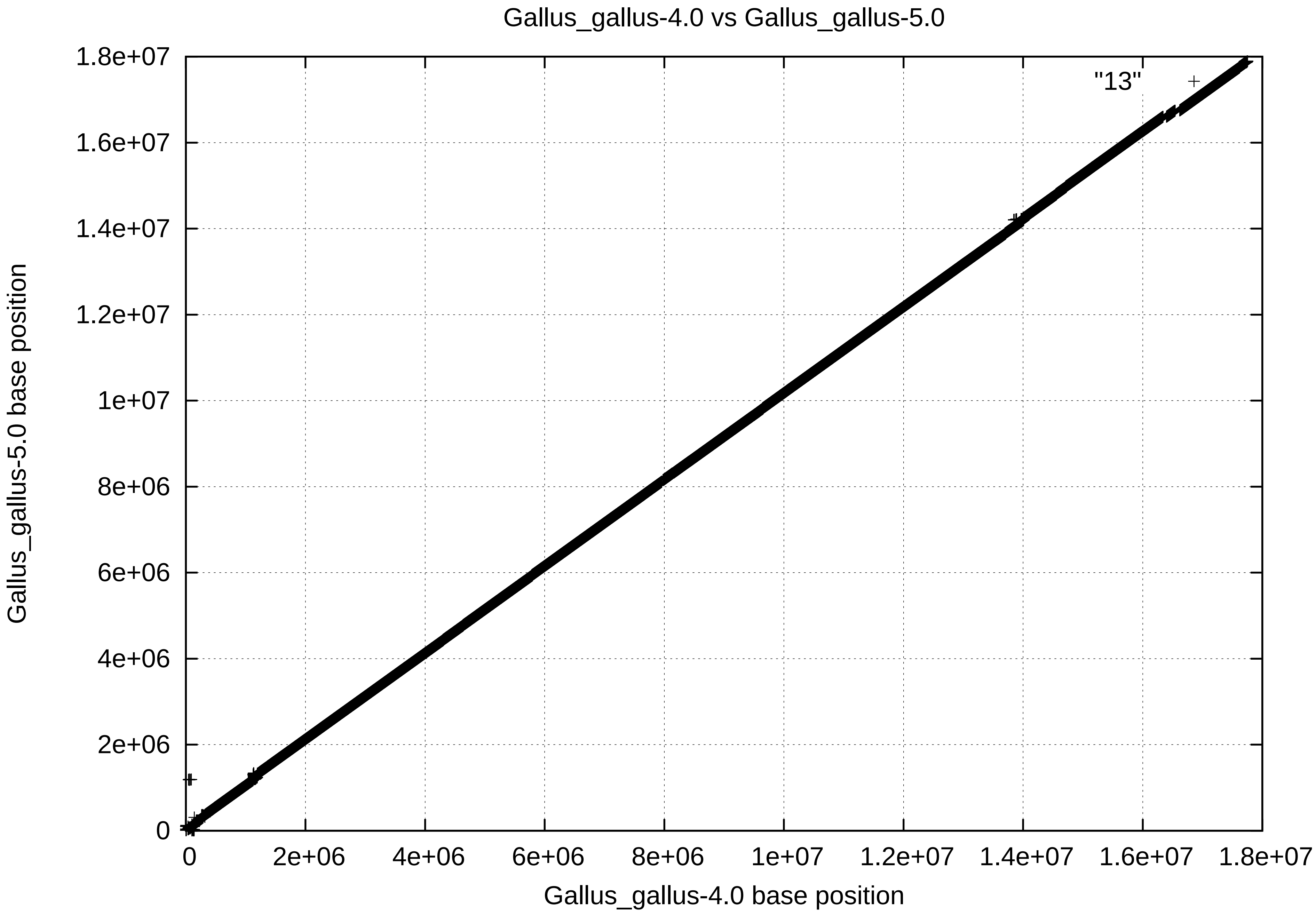

Gallus_gallus-4.0 vs Gallus_gallus-5.0
"13"
Gallus_gallus-5.0 base position
Gallus_gallus-4.0 base position
0
2e+06
4e+06
6e+06
8e+06
1e+07
1.2e+07
1.4e+07
1.6e+07
1.8e+07

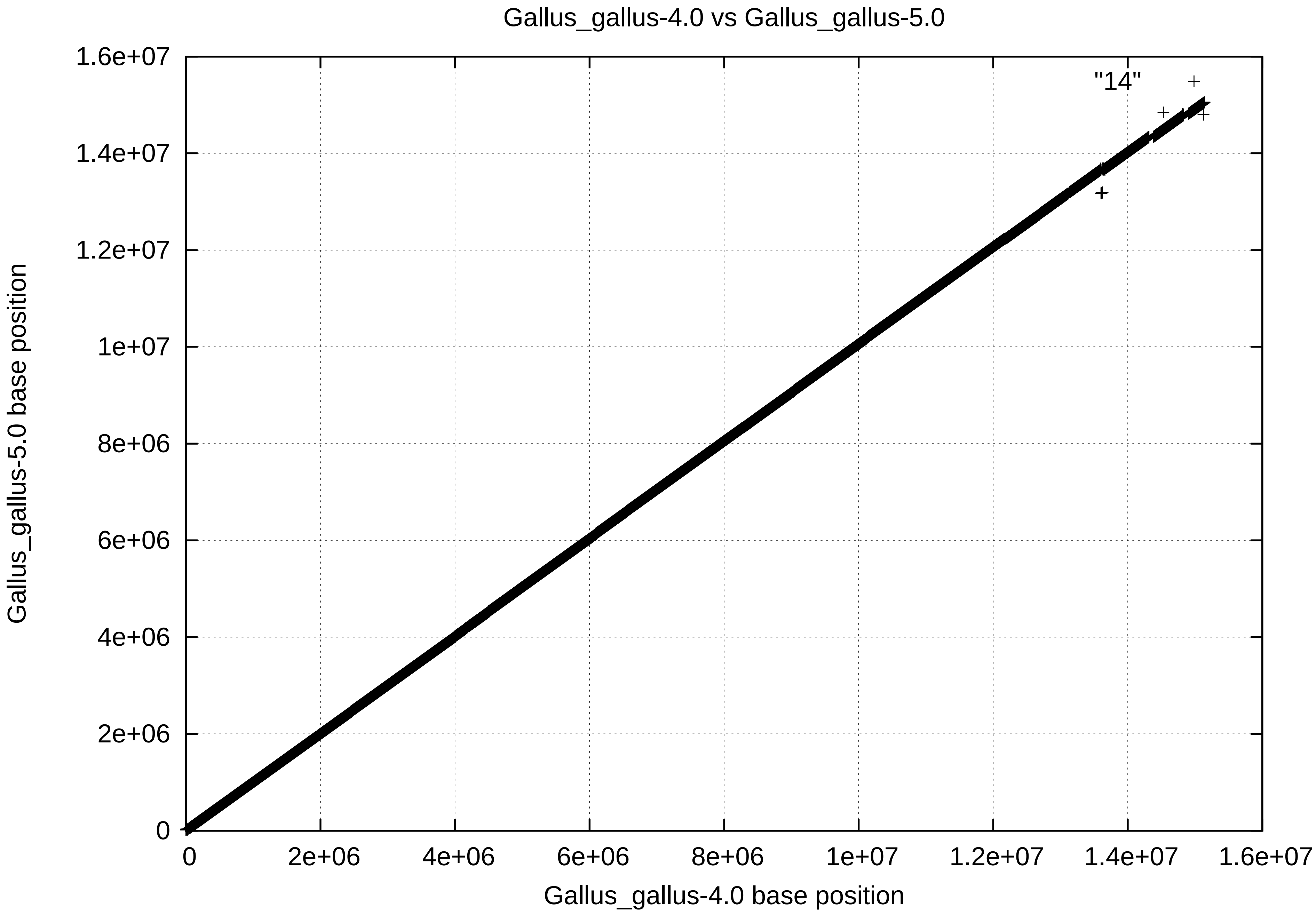

Gallus_gallus-4.0 vs Gallus_gallus-5.0
"14"
1.6e+07
1.4e+07
1.2e+07
1e+07
8e+06
6e+06
4e+06
2e+06
0
0
2e+06
4e+06
6e+06
8e+06
1e+07
1.2e+07
1.4e+07
1.6e+07
Gallus_gallus-5.0 base position
Gallus_gallus-4.0 base position

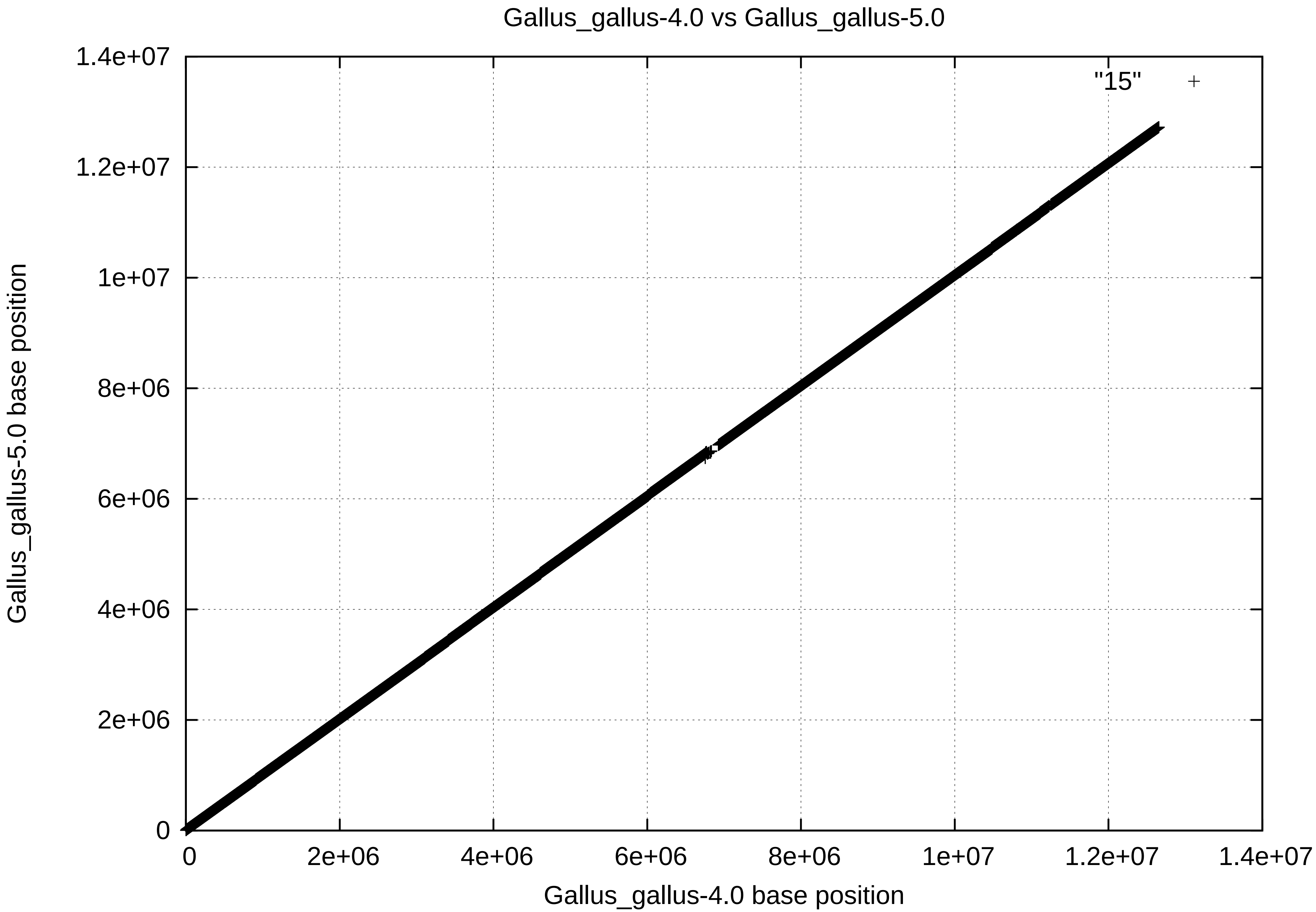
Gallus_gallus-4.0 vs Gallus_gallus-5.0
"15"
1.4e+07
1.2e+07
1e+07
8e+06
6e+06
4e+06
2e+06
0
0
2e+06
4e+06
6e+06
8e+06
1e+07
1.2e+07
1.4e+07
Gallus_gallus-4.0 base position
Gallus_gallus-5.0 base position

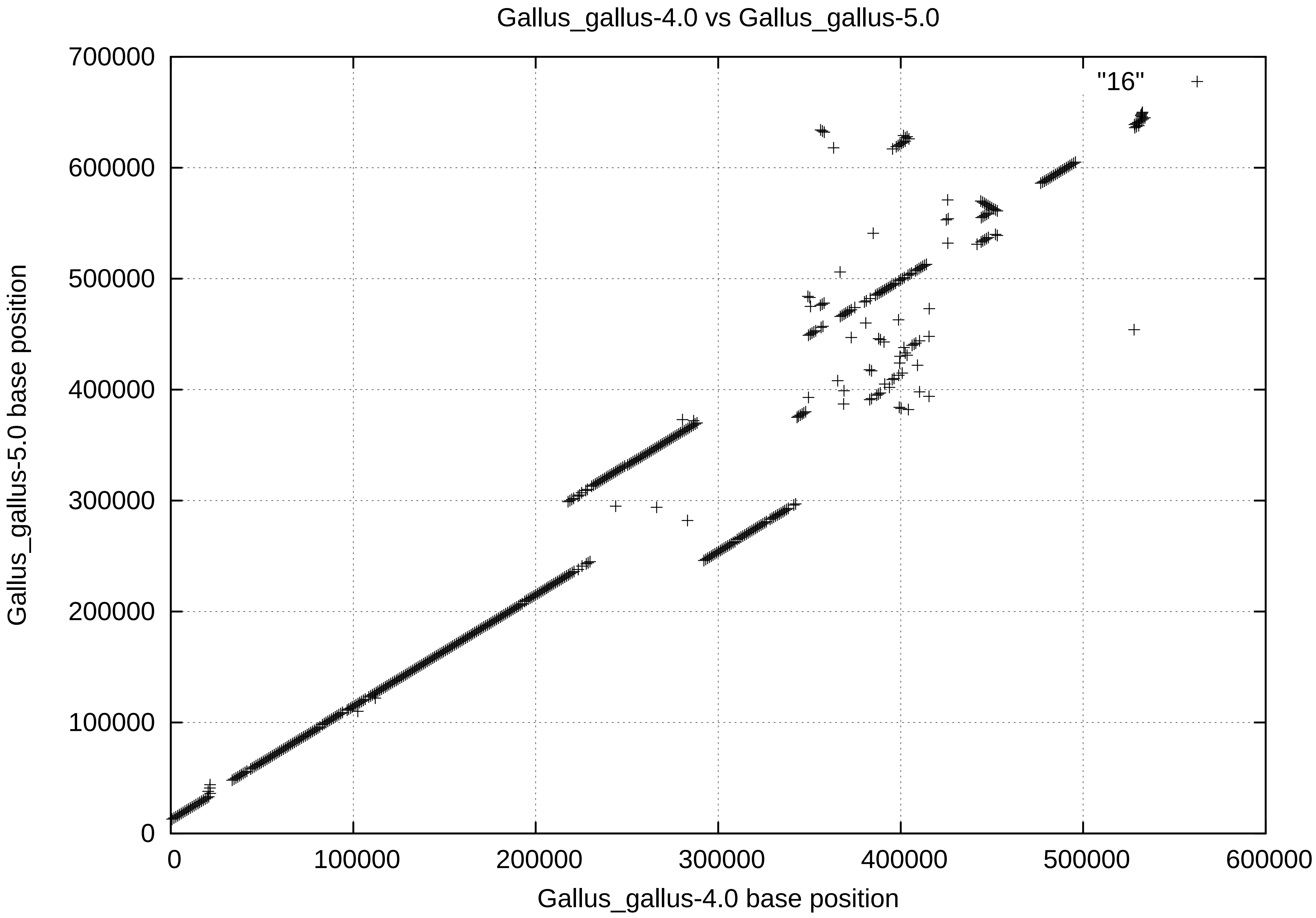
Gallus_gallus-4.0 vs Gallus_gallus-5.0
"16"
700000
600000
500000
400000
300000
200000
100000
0
0
100000
200000
300000
400000
500000
600000
Gallus_gallus-4.0 base position
Gallus_gallus-5.0 base position

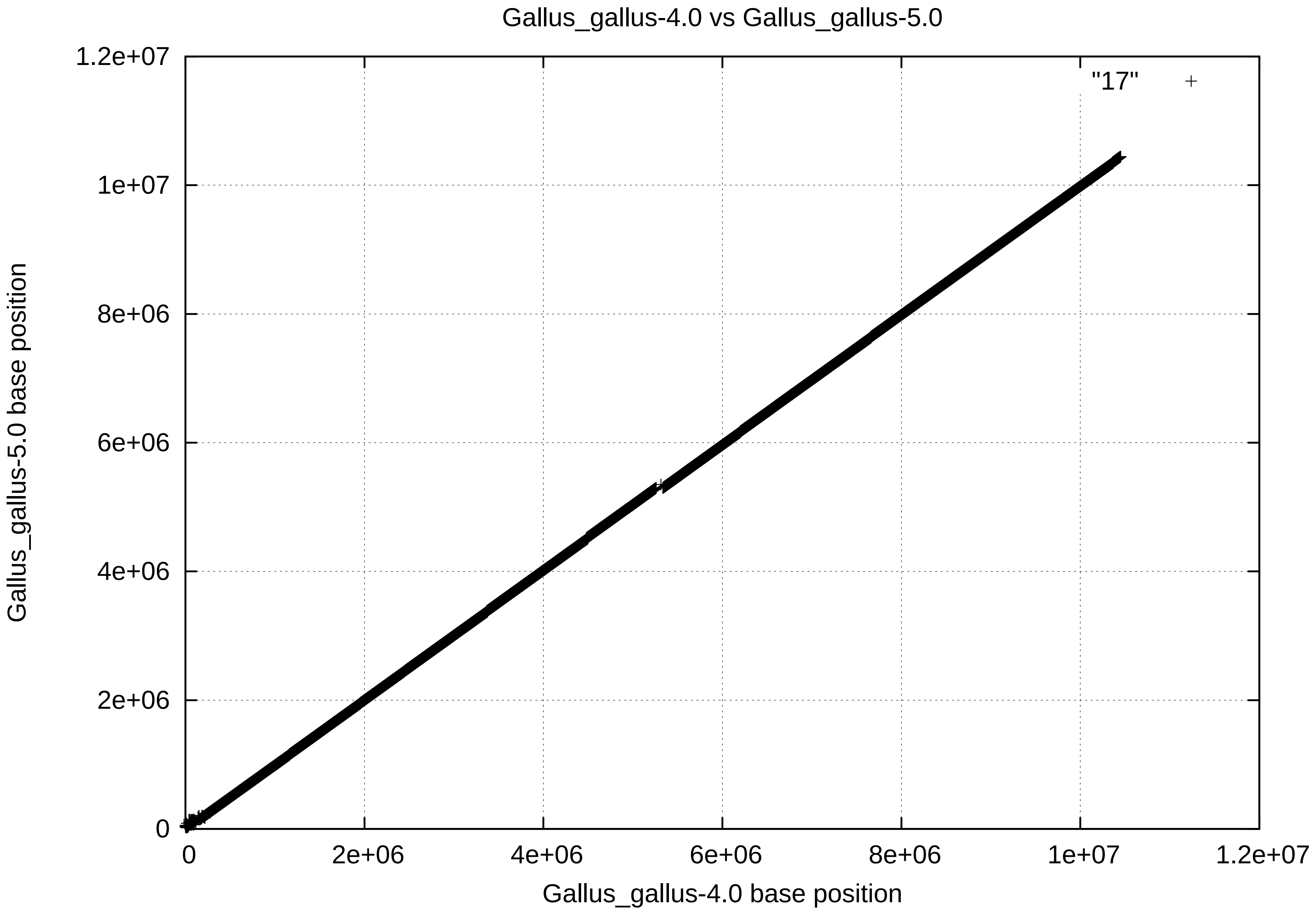

Gallus_gallus-4.0 vs Gallus_gallus-5.0
"17"
1.2e+07
1e+07
8e+06
6e+06
4e+06
2e+06
0
0
2e+06
4e+06
6e+06
8e+06
1e+07
1.2e+07
Gallus_gallus-4.0 base position
Gallus_gallus-5.0 base position

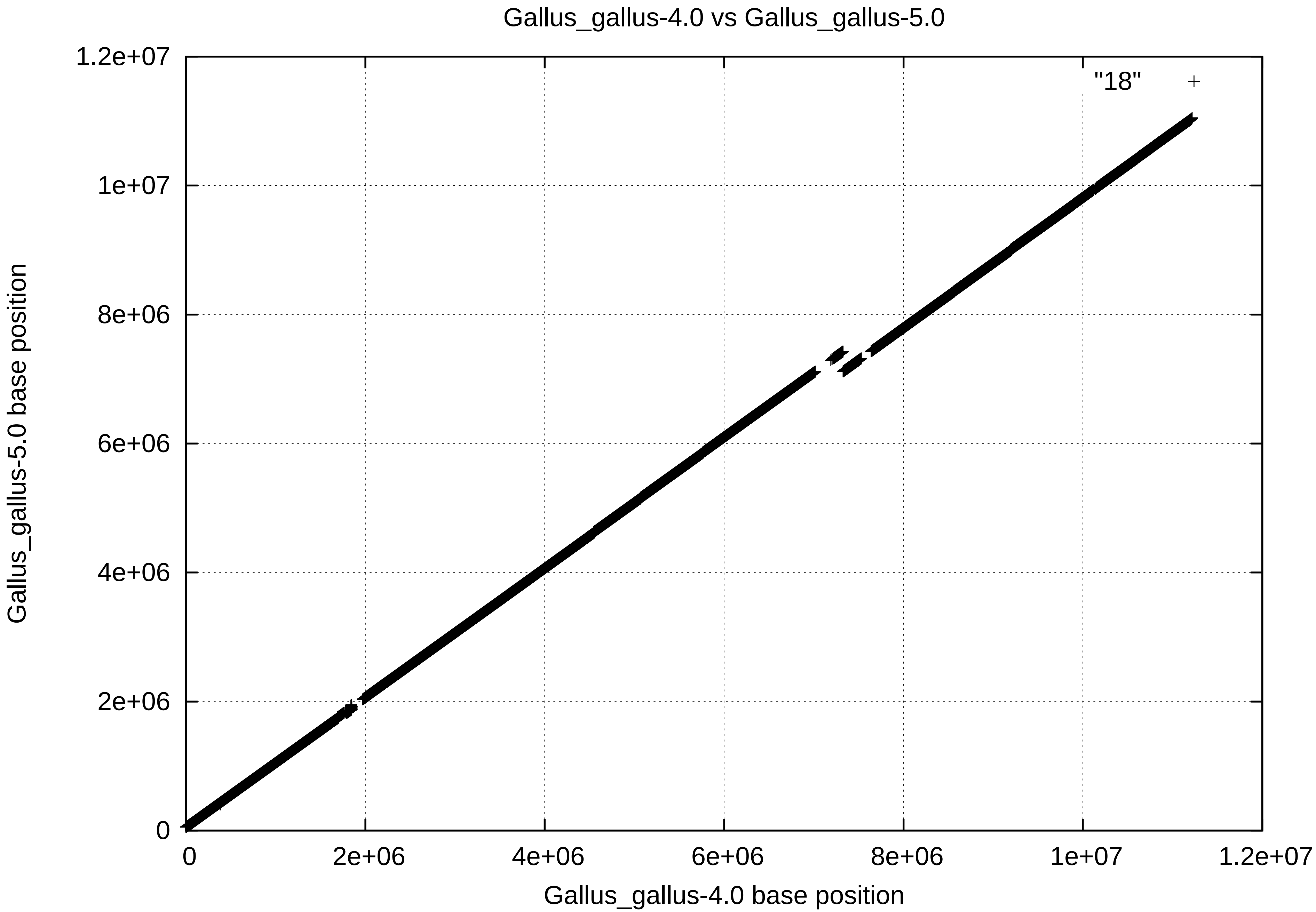
Gallus_gallus-4.0 vs Gallus_gallus-5.0
"18"
Gallus_gallus-5.0 base position
Gallus_gallus-4.0 base position
0
2e+06
4e+06
6e+06
8e+06
1e+07
1.2e+07

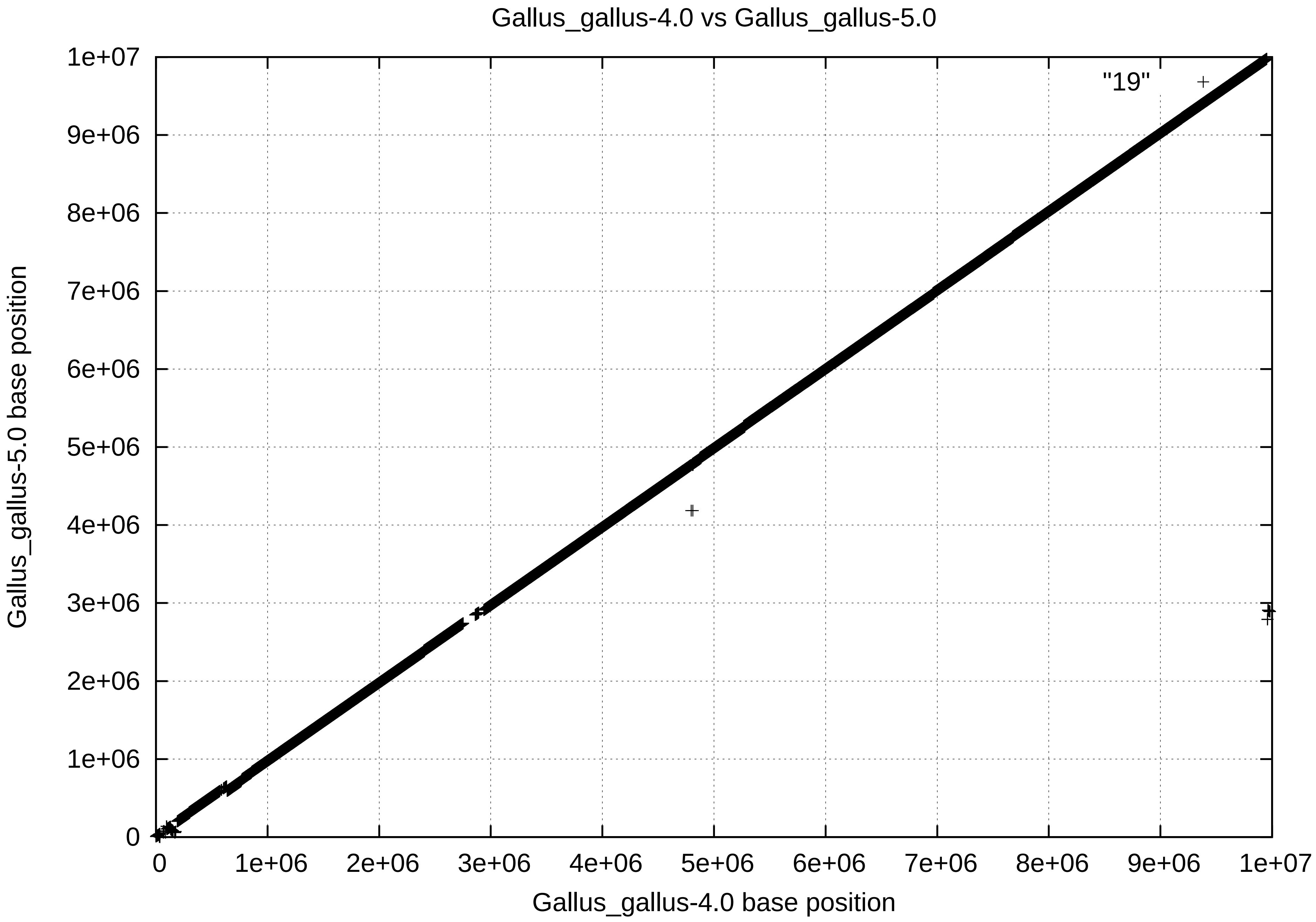

Gallus_gallus-4.0 vs Gallus_gallus-5.0
"19"
Gallus_gallus-5.0 base position
Gallus_gallus-4.0 base position
0
1e+06
2e+06
3e+06
4e+06
5e+06
6e+06
7e+06
8e+06
9e+06
1e+07

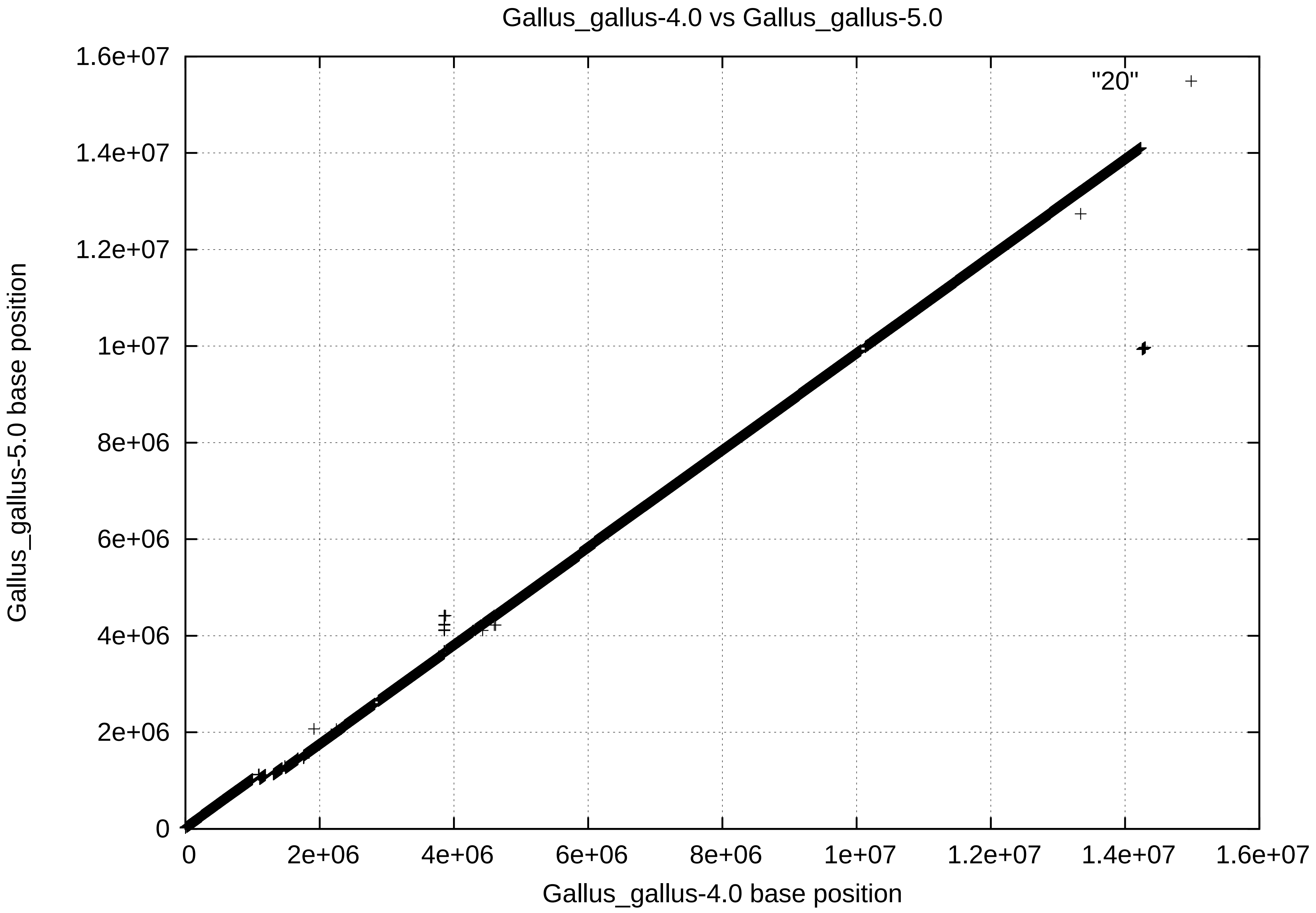

Gallus_gallus-4.0 vs Gallus_gallus-5.0
"20"
1.6e+07
1.4e+07
1.2e+07
1e+07
8e+06
6e+06
4e+06
2e+06
0
Gallus_gallus-5.0 base position
0
2e+06
4e+06
6e+06
8e+06
1e+07
1.2e+07
1.4e+07
1.6e+07
Gallus_gallus-4.0 base position

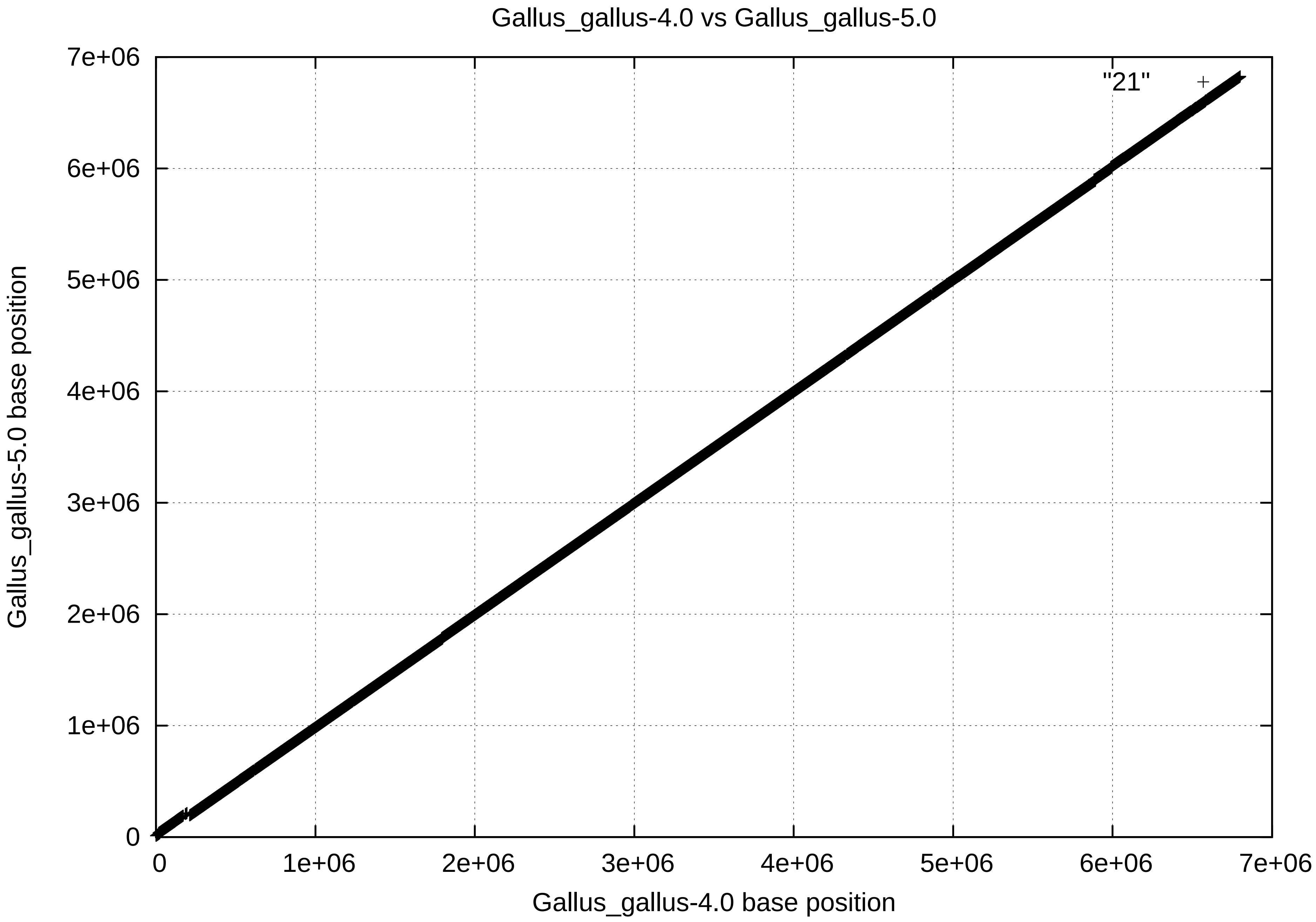
Gallus_gallus-4.0 vs Gallus_gallus-5.0
Gallus_gallus-5.0 base position
Gallus_gallus-4.0 base position
"21"
0
1e+06
2e+06
3e+06
4e+06
5e+06
6e+06
7e+06

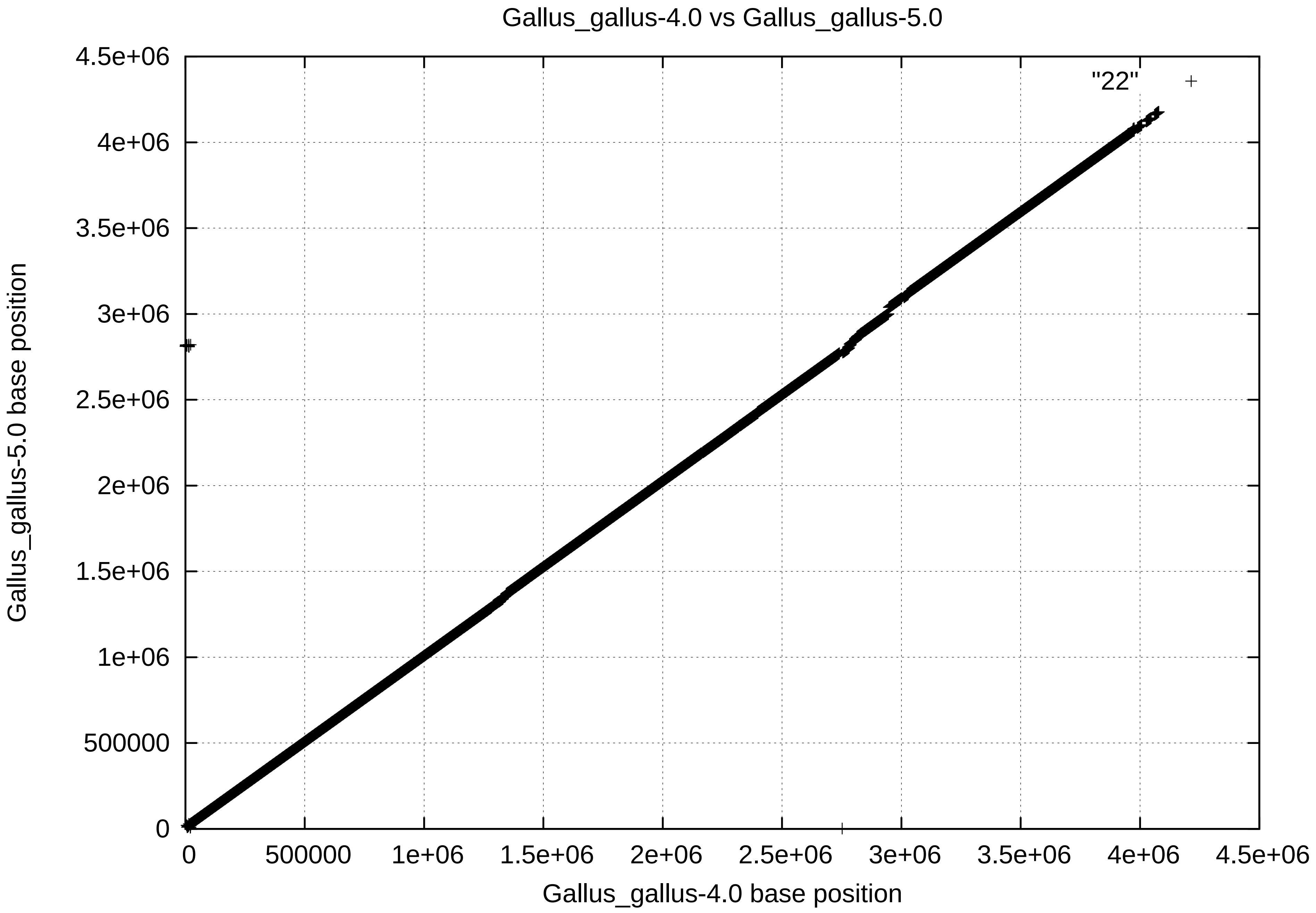

Gallus_gallus-4.0 vs Gallus_gallus-5.0
"22"
4.5e+06
4e+06
3.5e+06
3e+06
2.5e+06
2e+06
1.5e+06
1e+06
500000
0
Gallus_gallus-5.0 base position
0
500000
1e+06
1.5e+06
2e+06
2.5e+06
3e+06
3.5e+06
4e+06
4.5e+06
Gallus_gallus-4.0 base position

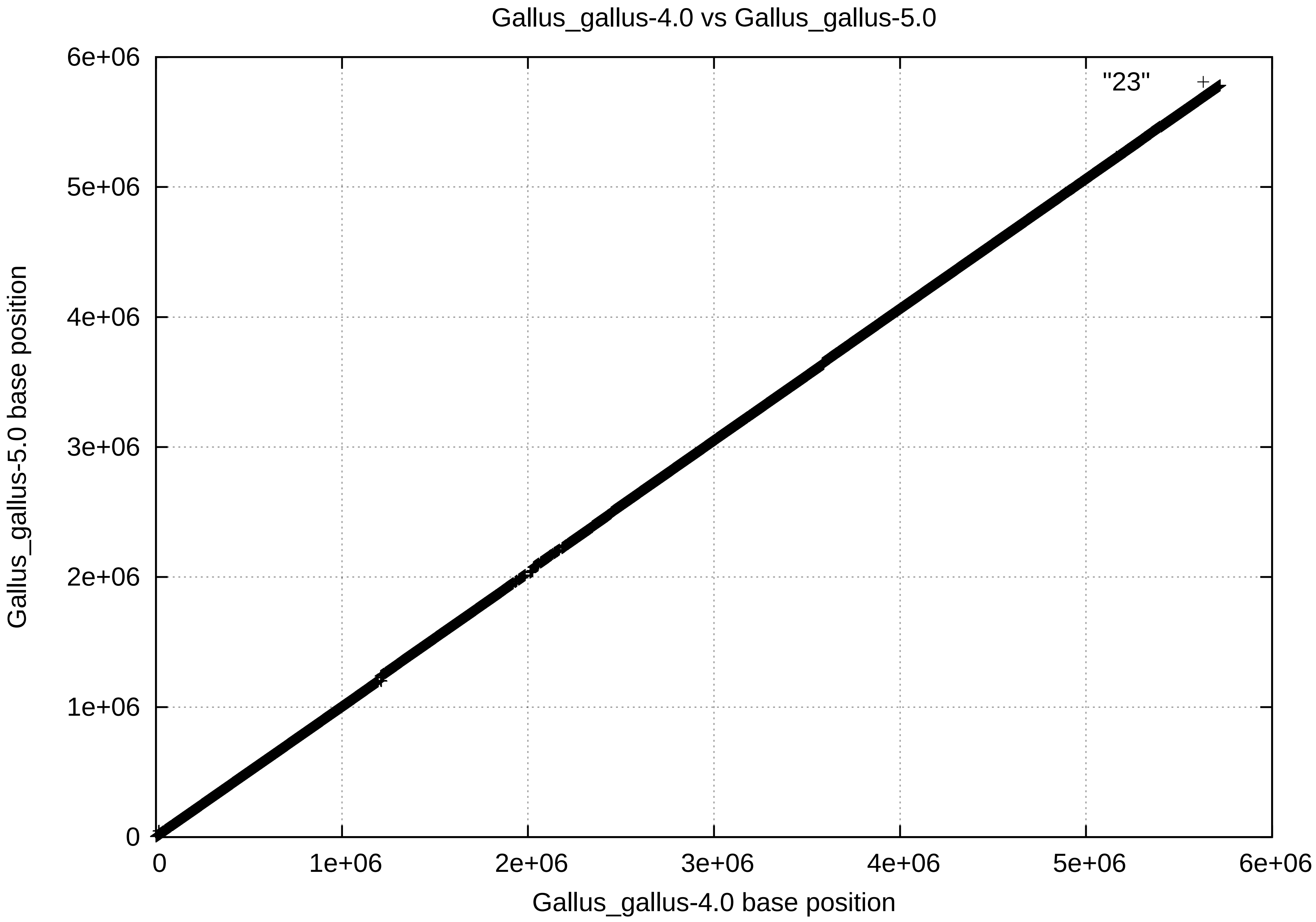
Gallus_gallus-4.0 vs Gallus_gallus-5.0
"23"
Gallus_gallus-5.0 base position
Gallus_gallus-4.0 base position
0
1e+06
2e+06
3e+06
4e+06
5e+06
6e+06

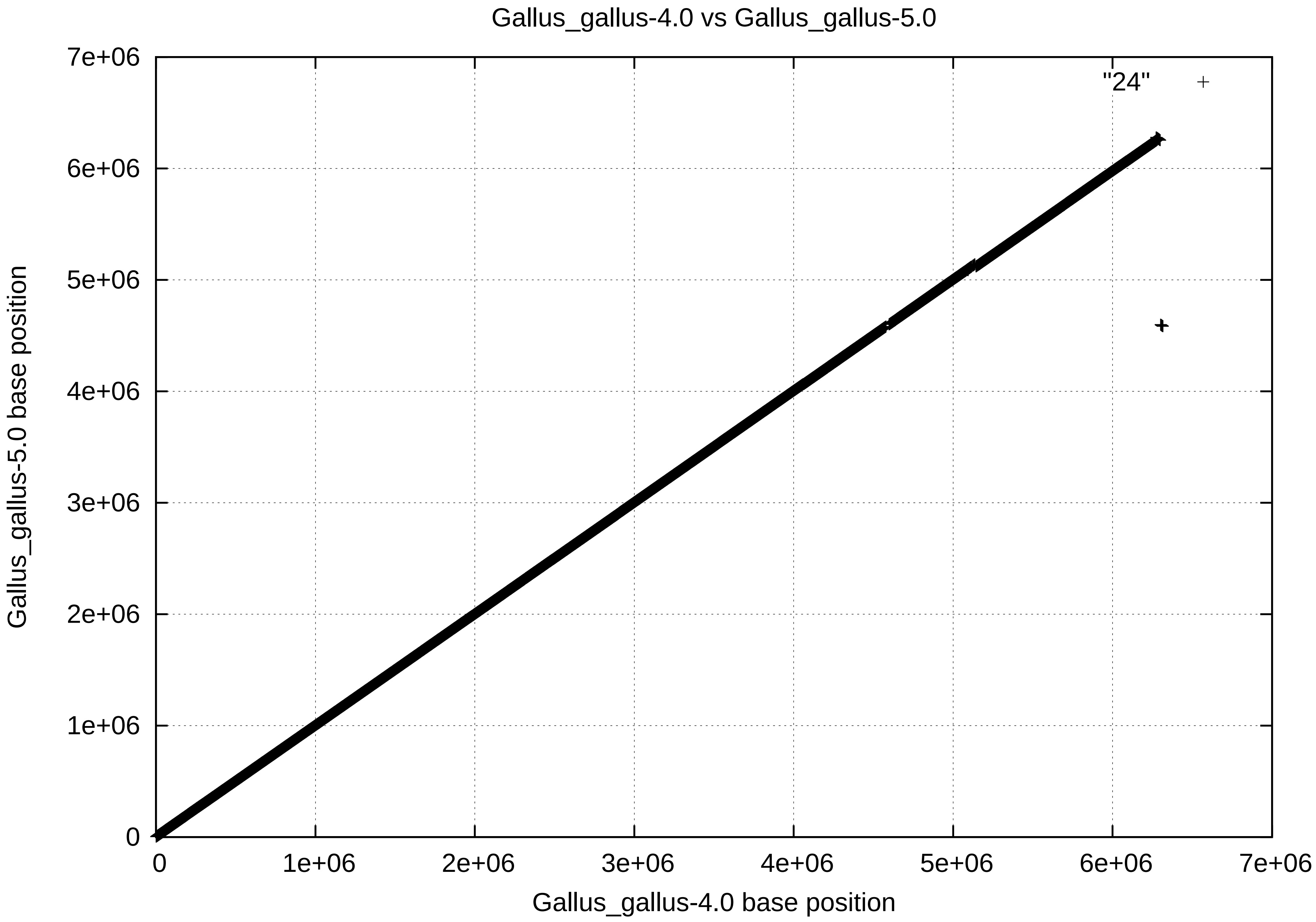

Gallus_gallus-4.0 vs Gallus_gallus-5.0
"24"
Gallus_gallus-5.0 base position
Gallus_gallus-4.0 base position
0
1e+06
2e+06
3e+06
4e+06
5e+06
6e+06
7e+06

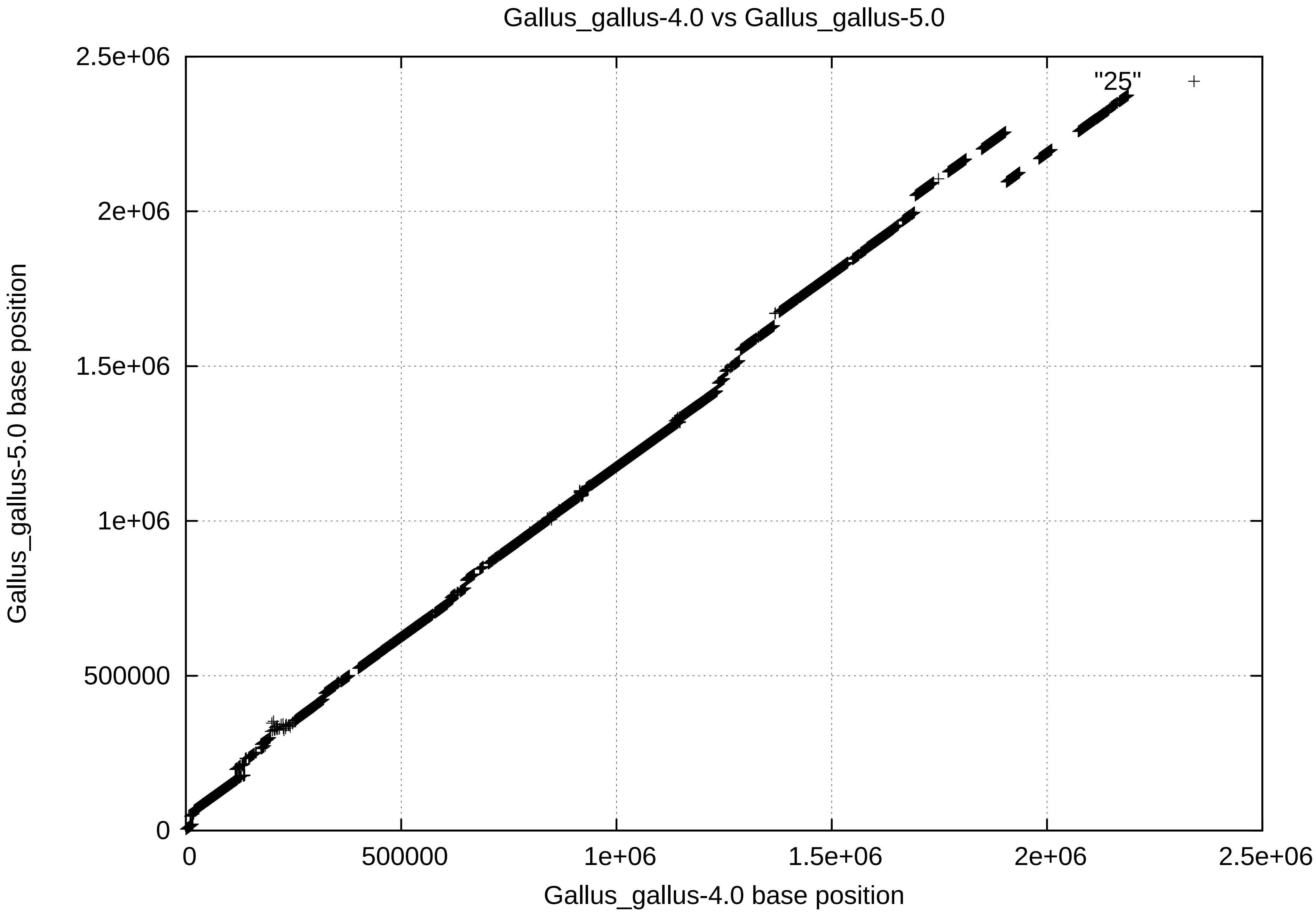

Gallus_gallus-4.0 vs Gallus_gallus-5.0
"25"
2.5e+06
2e+06
1.5e+06
1e+06
500000
0
500000
1e+06
1.5e+06
2e+06
2.5e+06
Gallus_gallus-4.0 base position
Gallus_gallus-5.0 base position

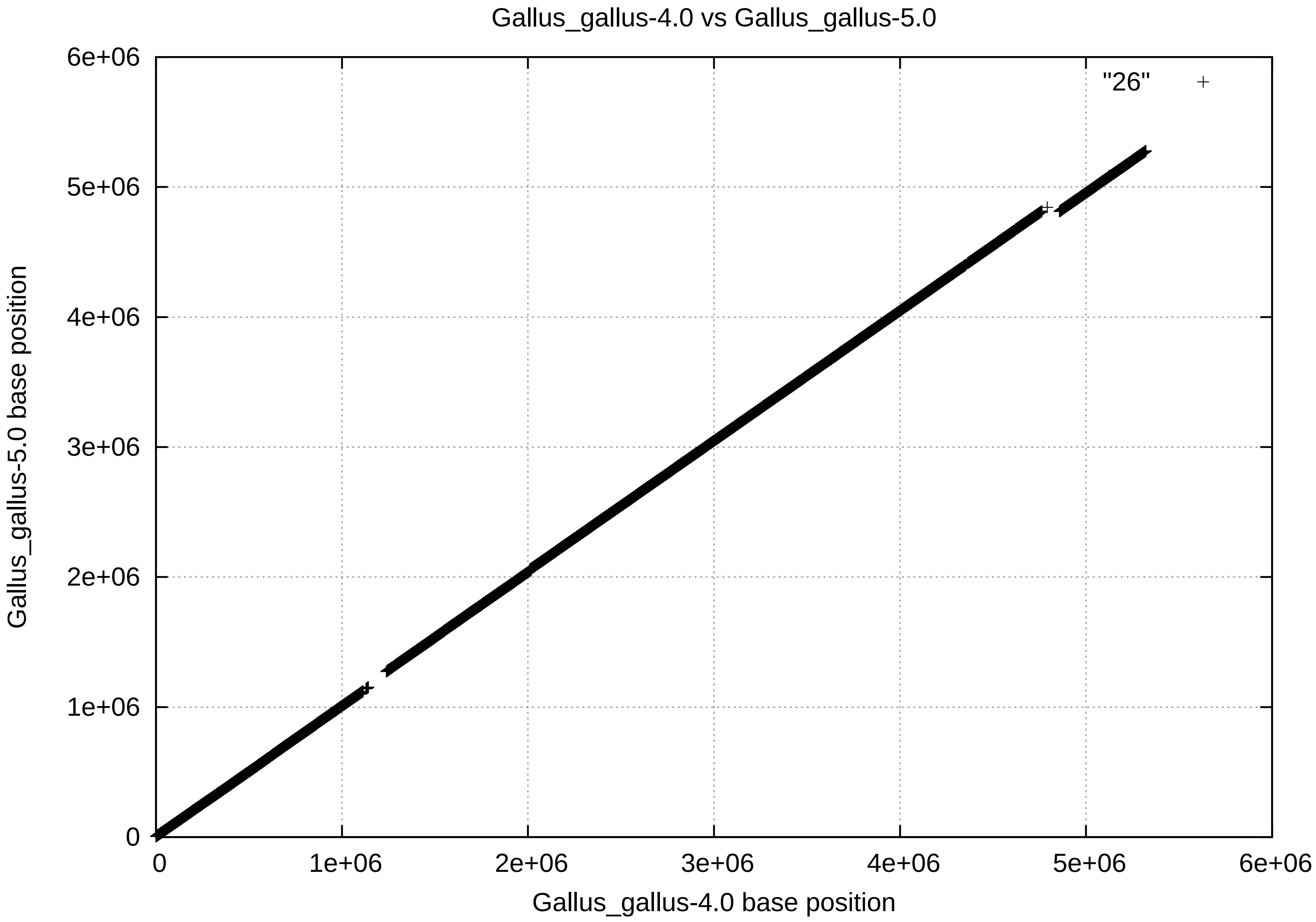
Gallus_gallus-4.0 vs Gallus_gallus-5.0
"26"
Gallus_gallus-5.0 base position
Gallus_gallus-4.0 base position
0
1e+06
2e+06
3e+06
4e+06
5e+06
6e+06

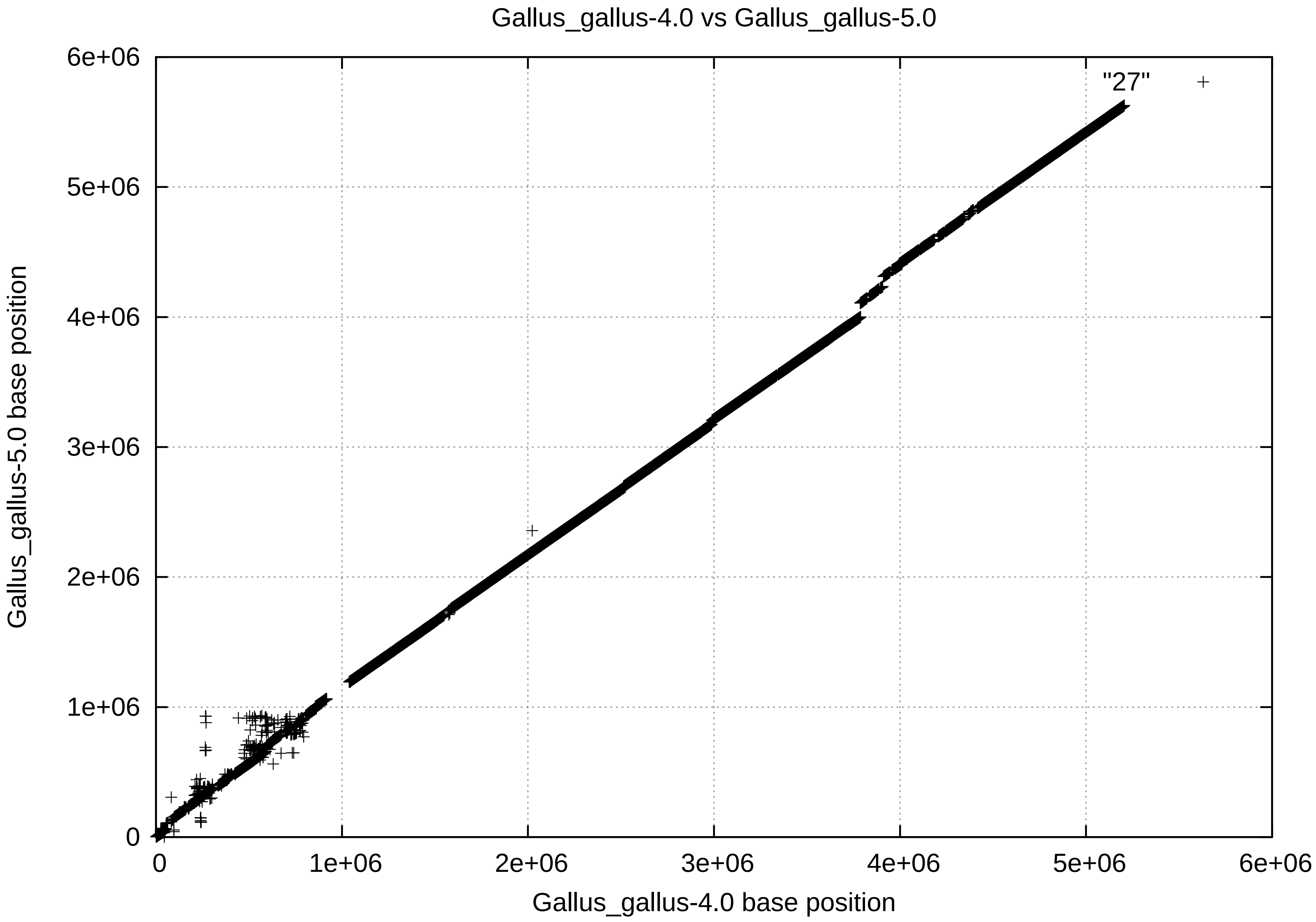
Gallus_gallus-4.0 vs Gallus_gallus-5.0
"27"
Gallus_gallus-5.0 base position
Gallus_gallus-4.0 base position
0
1e+06
2e+06
3e+06
4e+06
5e+06
6e+06

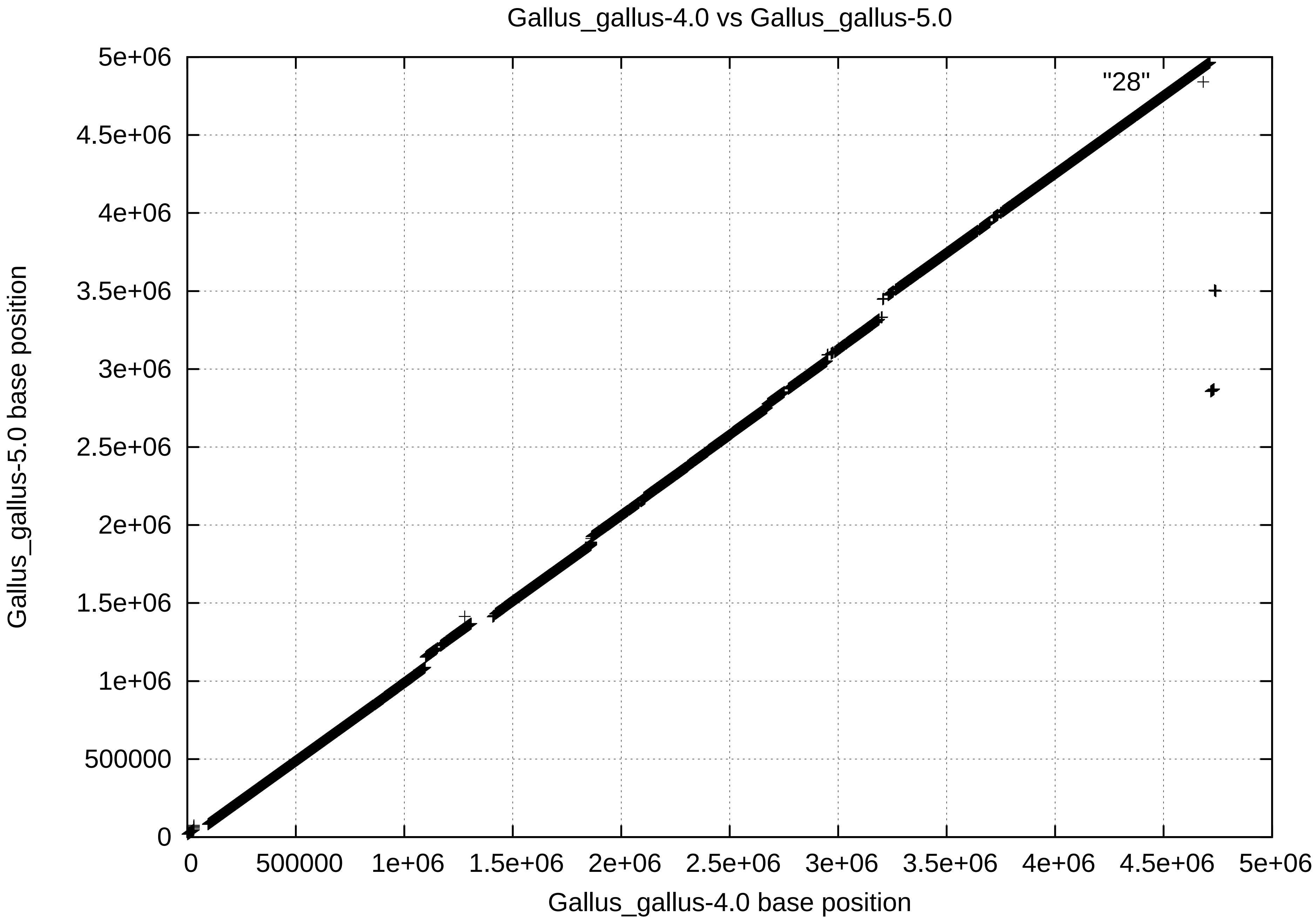

Gallus_gallus-4.0 vs Gallus_gallus-5.0
"28"
Gallus_gallus-5.0 base position
Gallus_gallus-4.0 base position
0
500000
1e+06
1.5e+06
2e+06
2.5e+06
3e+06
3.5e+06
4e+06
4.5e+06
5e+06

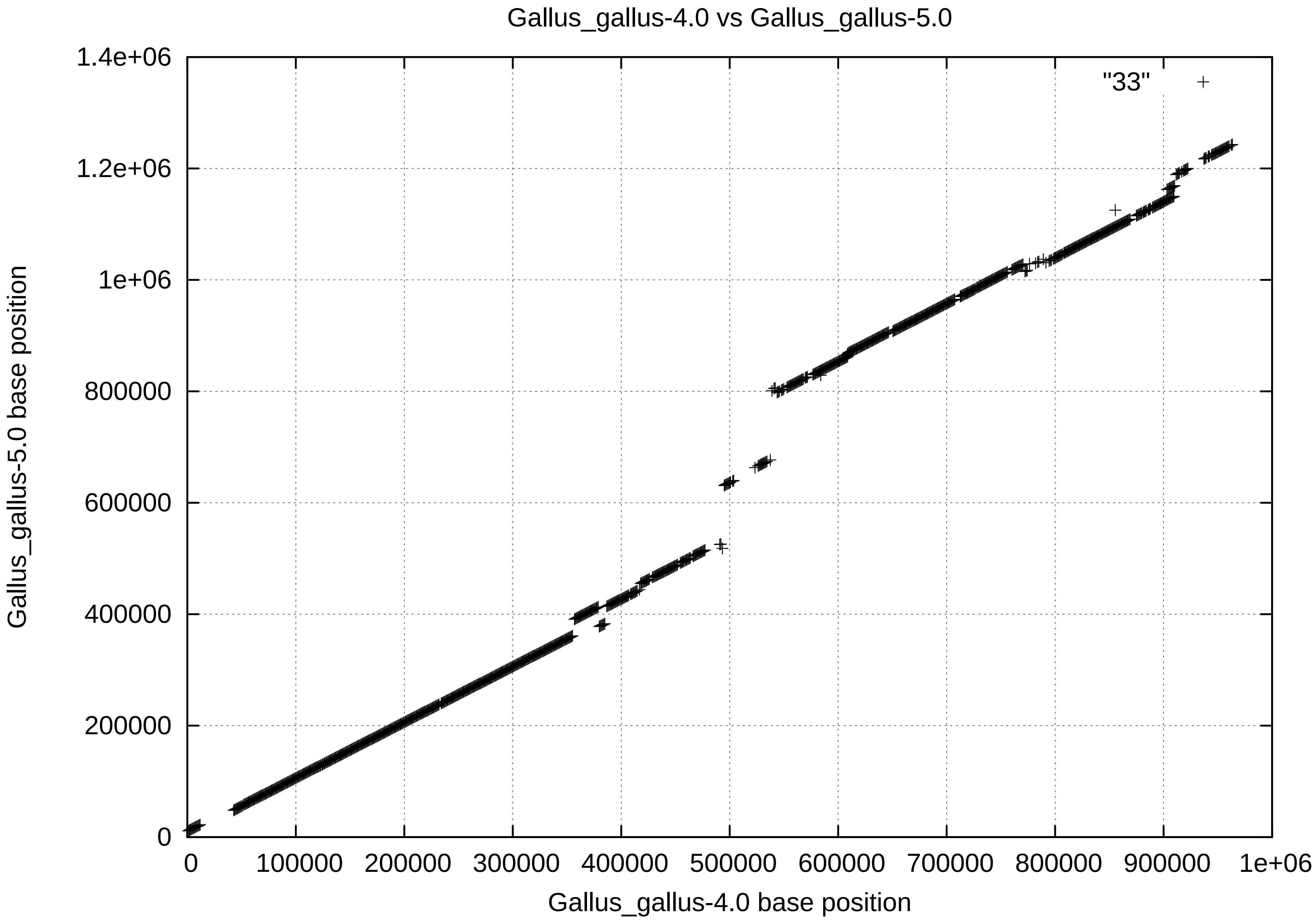
Gallus_gallus-4.0 vs Gallus_gallus-5.0
"33"
1.4e+06
1.2e+06
1e+06
800000
600000
400000
200000
0
Gallus_gallus-5.0 base position
0
100000
200000
300000
400000
500000
600000
700000
800000
900000
1e+06
Gallus_gallus-4.0 base position

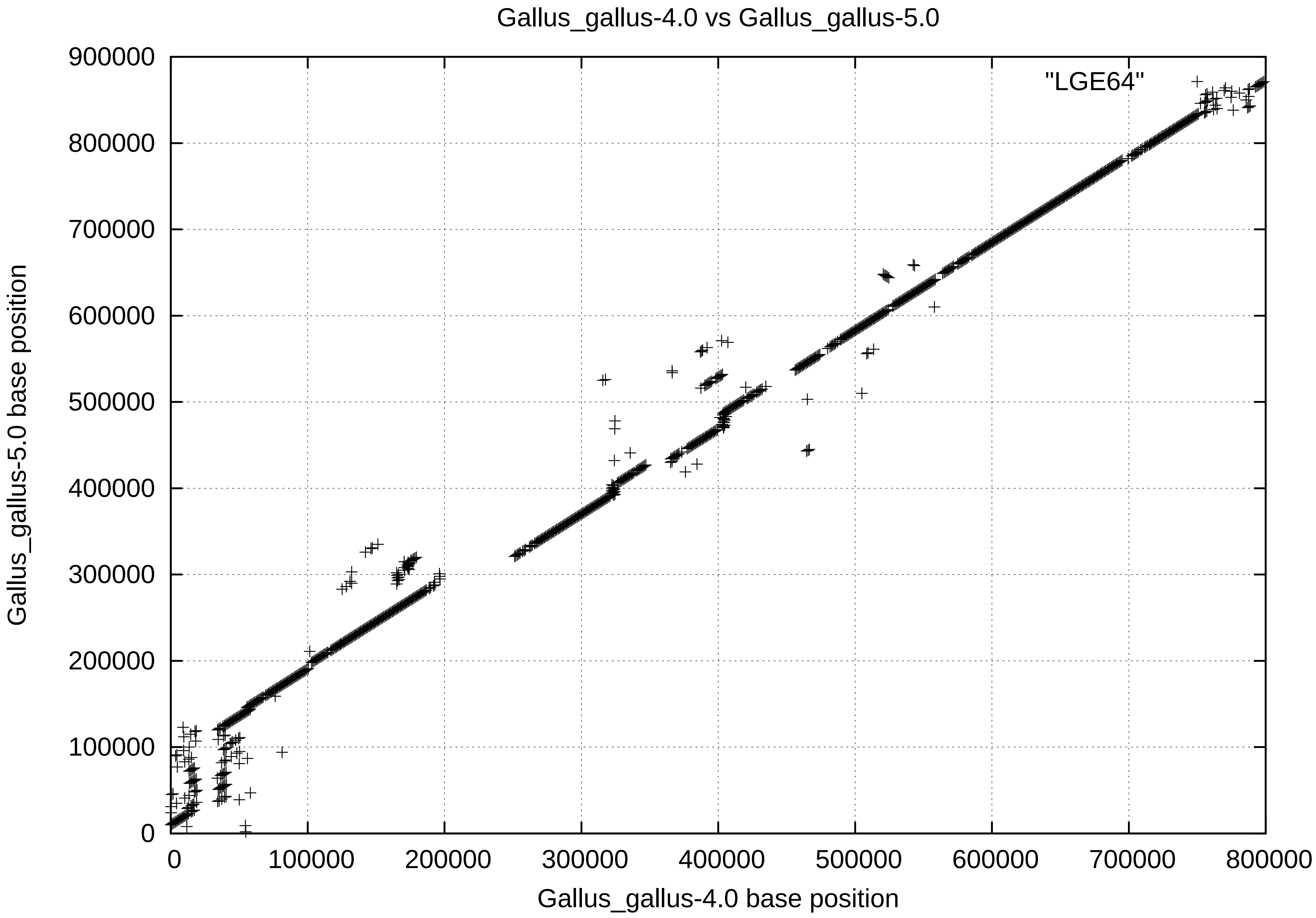

Gallus_gallus-4.0 vs Gallus_gallus-5.0
"LGE64"
900000
800000
700000
600000
500000
400000
300000
200000
100000
0
0
100000
200000
300000
400000
500000
600000
700000
800000
Gallus_gallus-4.0 base position
Gallus_gallus-5.0 base position
