# Supplementary material for: A New Chicken Genome Assembly Provides Insight into Avian Genome Structure
Source: G3 (Bethesda). 2016 Nov 14;7(1):109–17. doi: 10.1534/g3.116.035923 (PMC5217101; doi:10.1534/g3.116.035923)
Supplement: Supplementary file 9 [file 109FileS3.pdf]

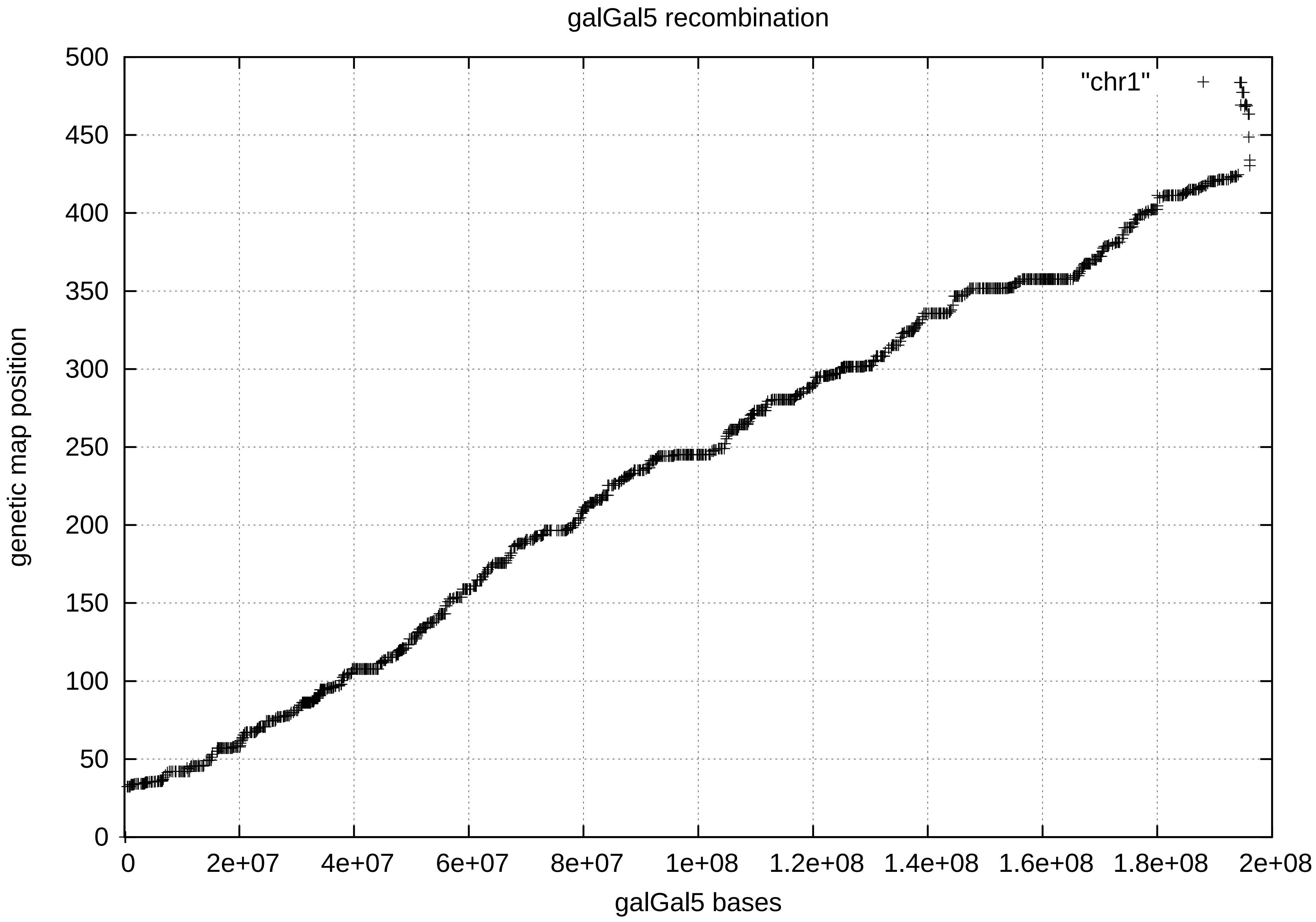
galGal5 recombination
genetic map position
galGal5 bases
"chr1"
0
50
100
150
200
250
300
350
400
450
500
0
2e+07
4e+07
6e+07
8e+07
1e+08
1.2e+08
1.4e+08
1.6e+08
1.8e+08
2e+08

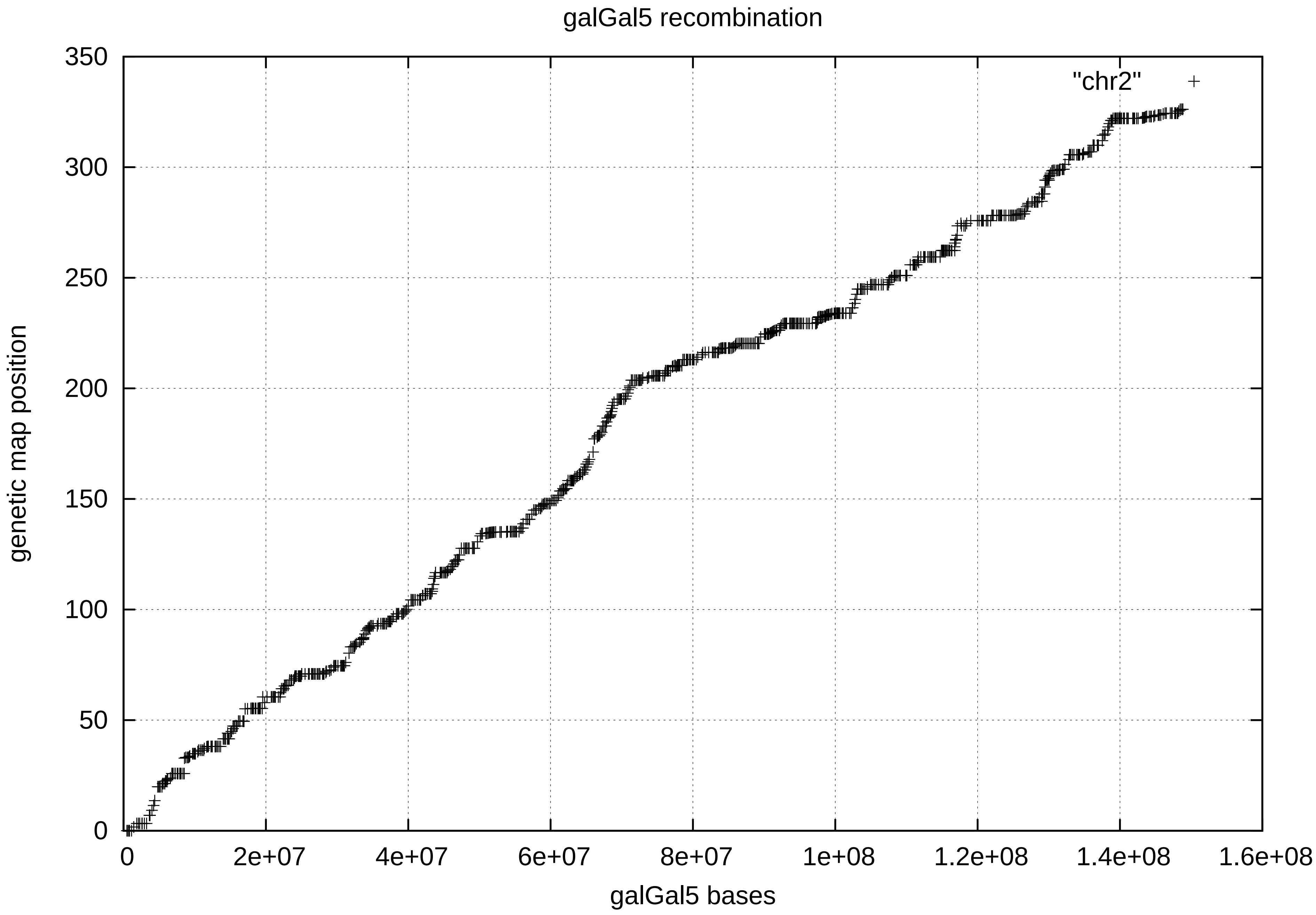
galGal5 recombination
"chr2"
genetic map position
350
300
250
200
150
100
50
0
0
2e+07
4e+07
6e+07
8e+07
1e+08
1.2e+08
1.4e+08
1.6e+08
galGal5 bases

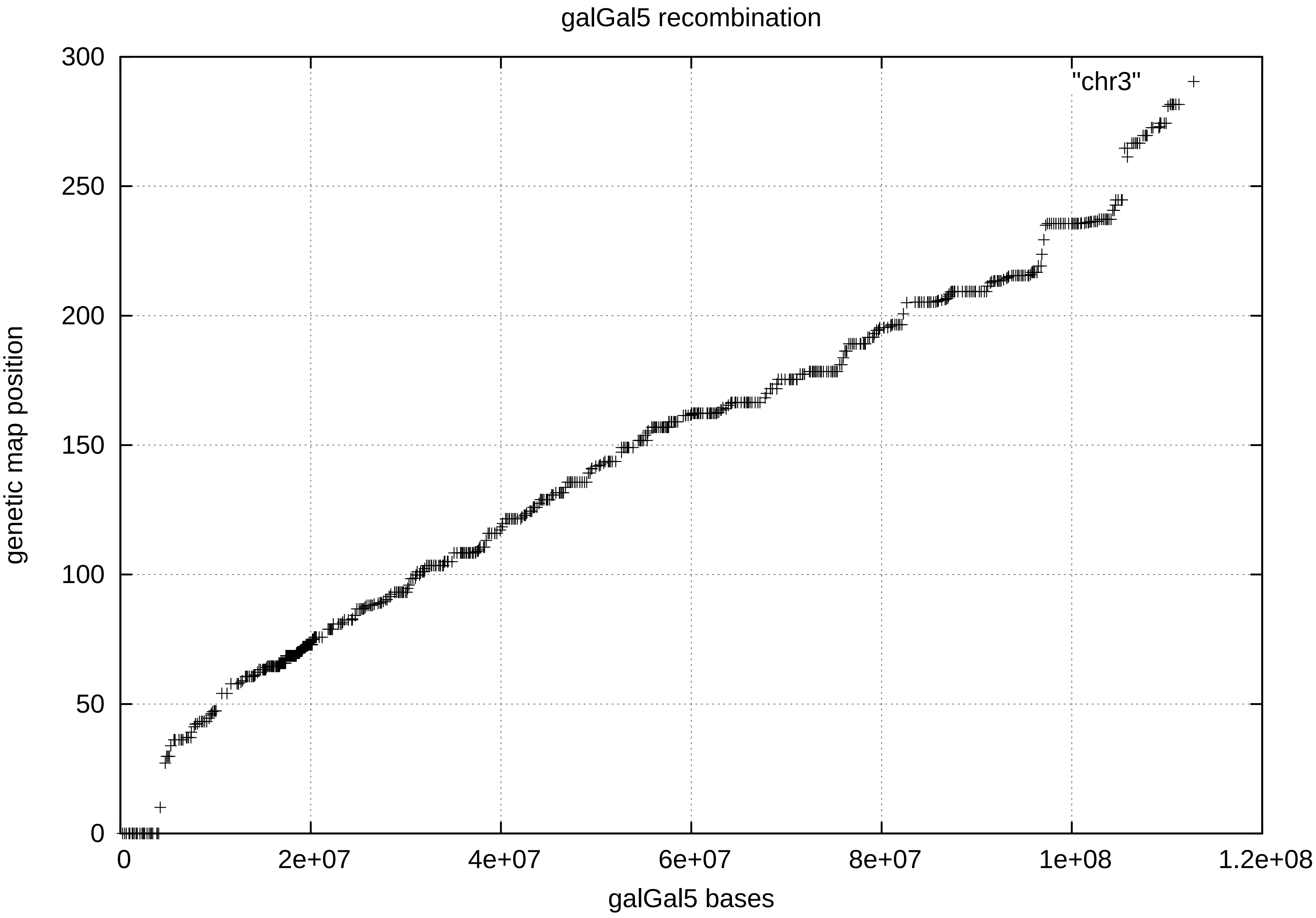
galGal5 recombination
"chr3"
genetic map position
galGal5 bases
0
50
100
150
200
250
300
0
2e+07
4e+07
6e+07
8e+07
1e+08
1.2e+08

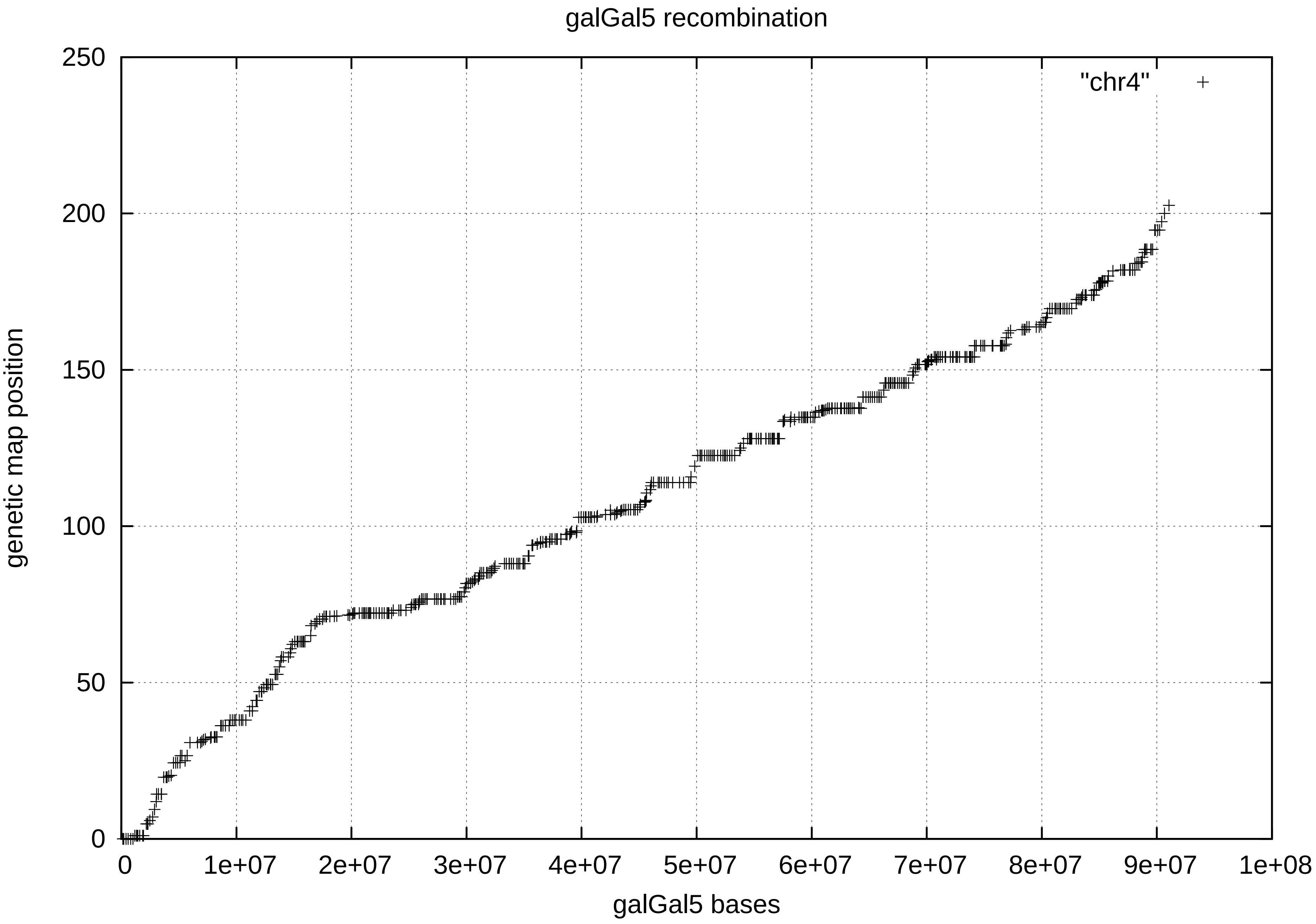

galGal5 recombination
"chr4"
genetic map position
galGal5 bases
0
50
100
150
200
250
0
1e+07
2e+07
3e+07
4e+07
5e+07
6e+07
7e+07
8e+07
9e+07
1e+08

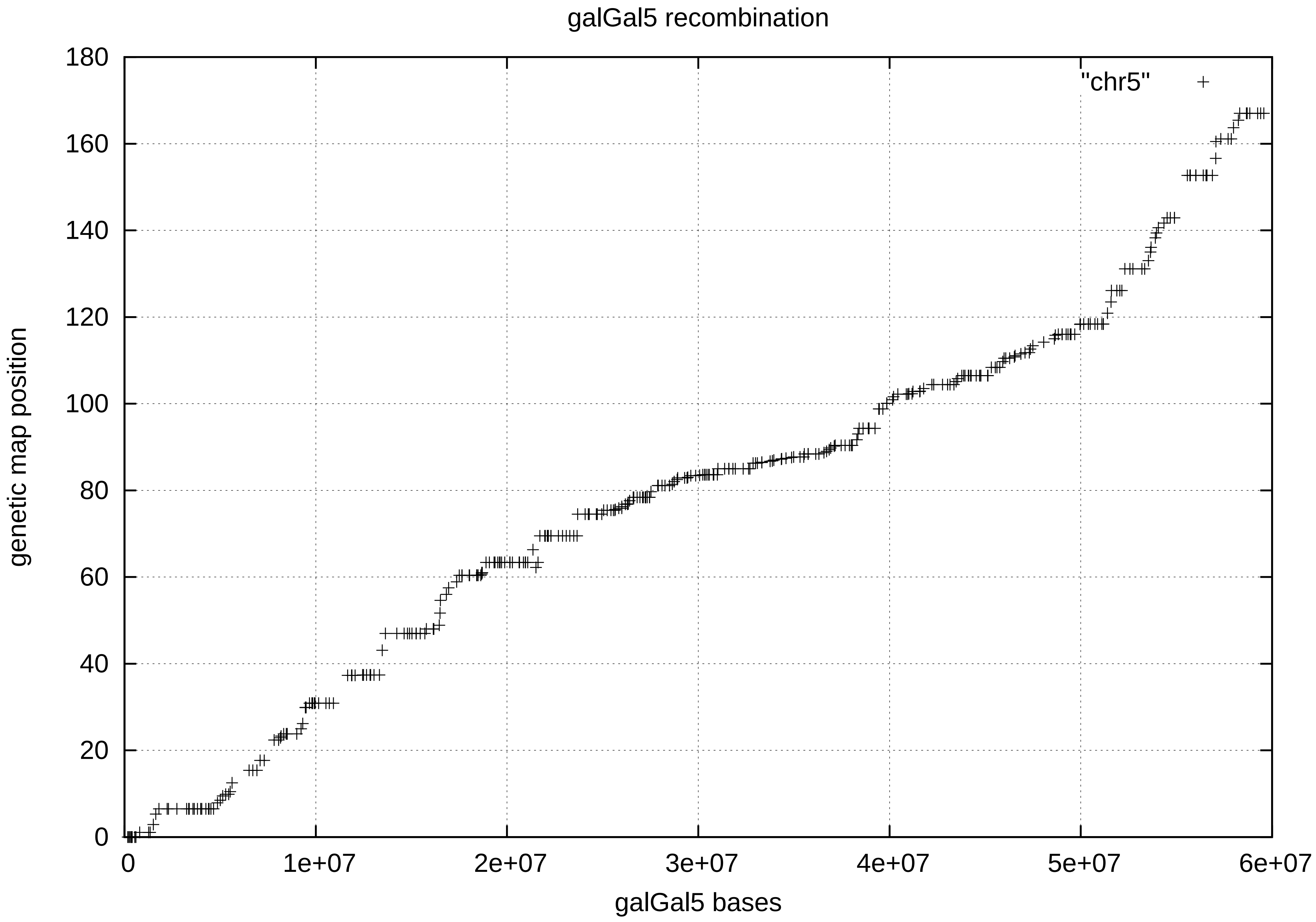
galGal5 recombination
"chr5"
180
160
140
120
100
80
60
40
20
0
0
1e+07
2e+07
3e+07
4e+07
5e+07
6e+07
genetic map position
galGal5 bases

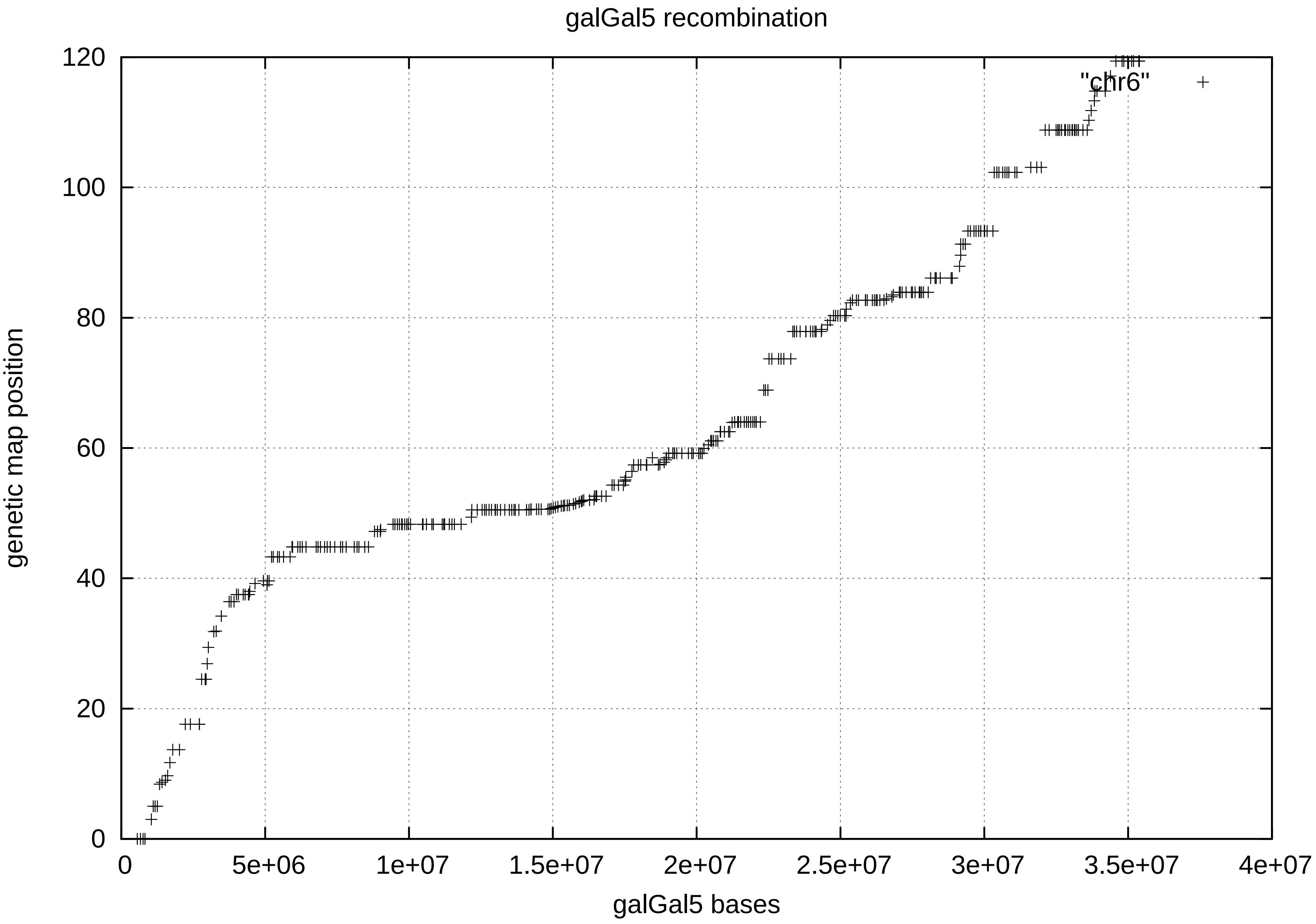

galGal5 recombination
"chr6"
genetic map position
galGal5 bases
0
20
40
60
80
100
120
0
5e+06
1e+07
1.5e+07
2e+07
2.5e+07
3e+07
3.5e+07
4e+07

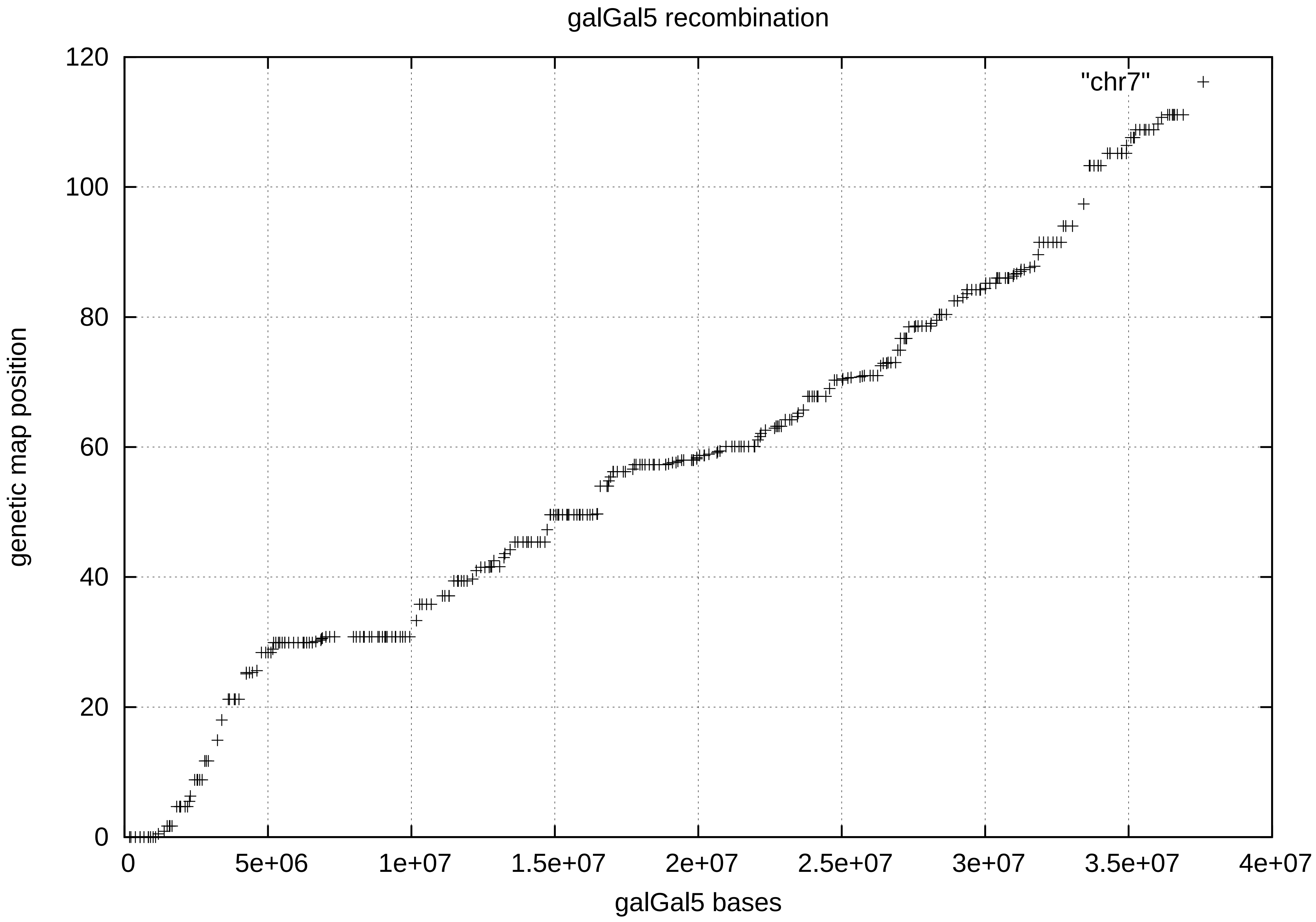
galGal5 recombination
genetic map position
galGal5 bases
"chr7"
0
20
40
60
80
100
120
0
5e+06
1e+07
1.5e+07
2e+07
2.5e+07
3e+07
3.5e+07
4e+07

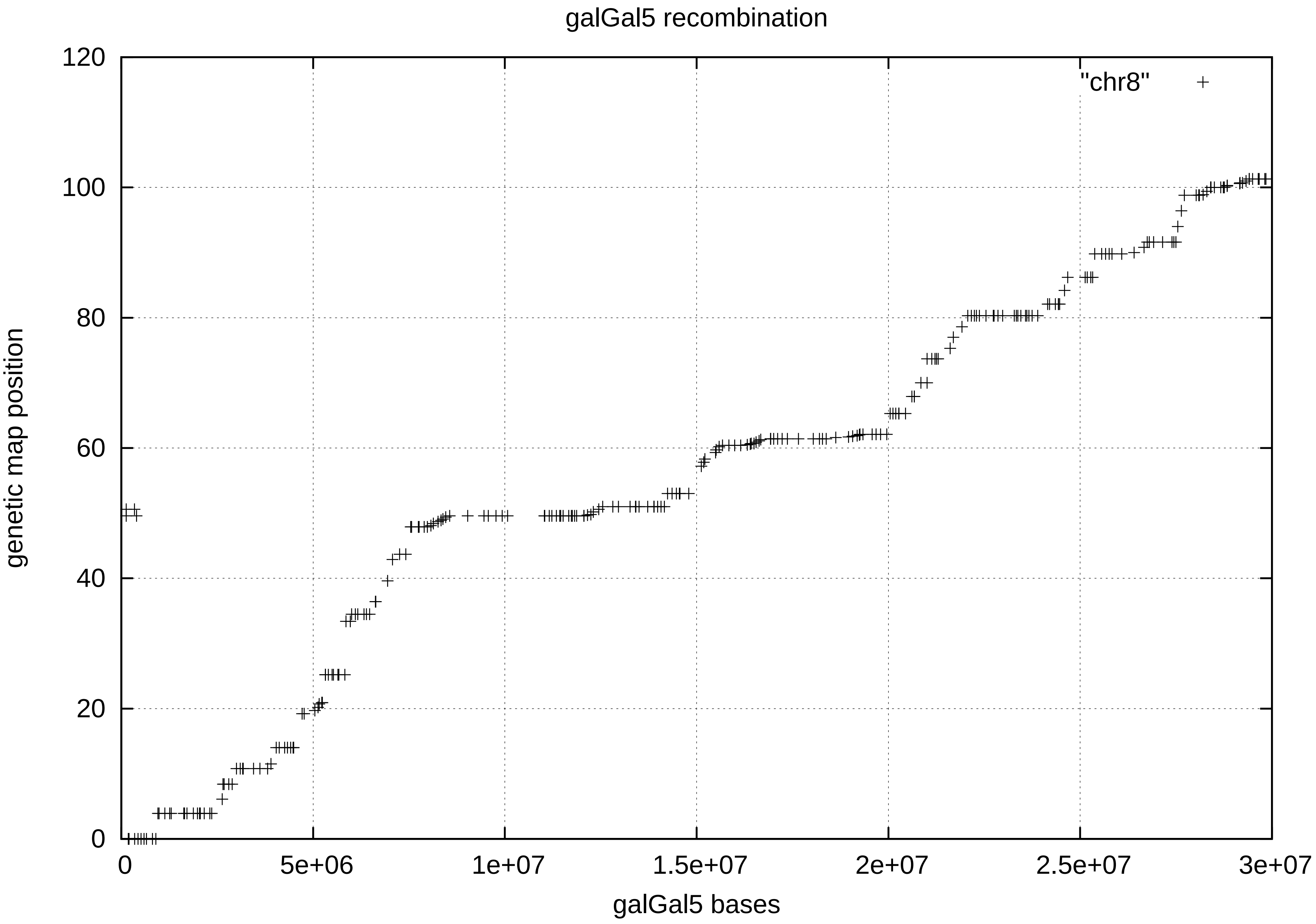
galGal5 recombination
"chr8"
genetic map position
galGal5 bases
0
20
40
60
80
100
120
0
5e+06
1e+07
1.5e+07
2e+07
2.5e+07
3e+07

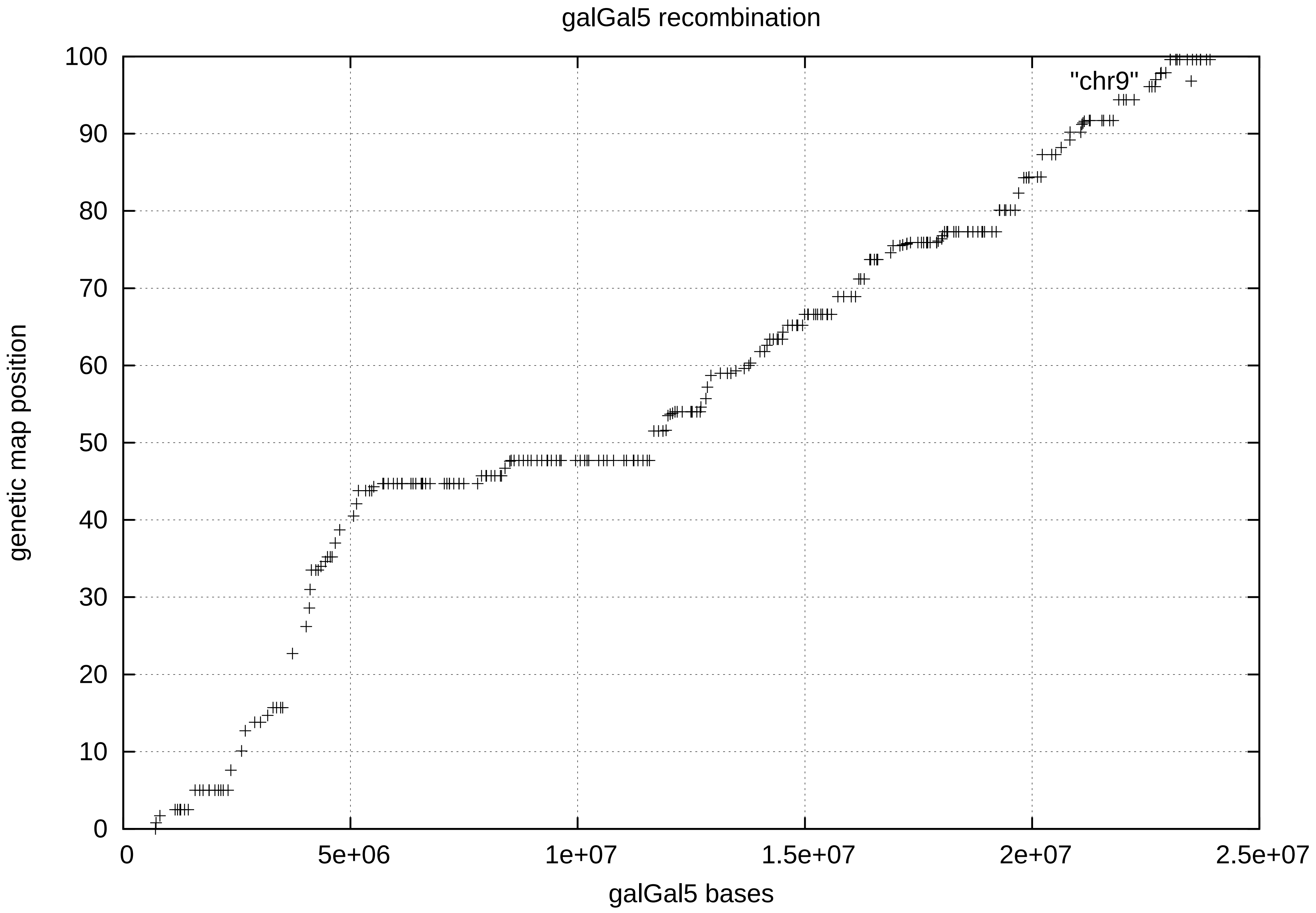
galGal5 recombination
genetic map position
galGal5 bases
"chr9"
0
10
20
30
40
50
60
70
80
90
100
0
5e+06
1e+07
1.5e+07
2e+07
2.5e+07

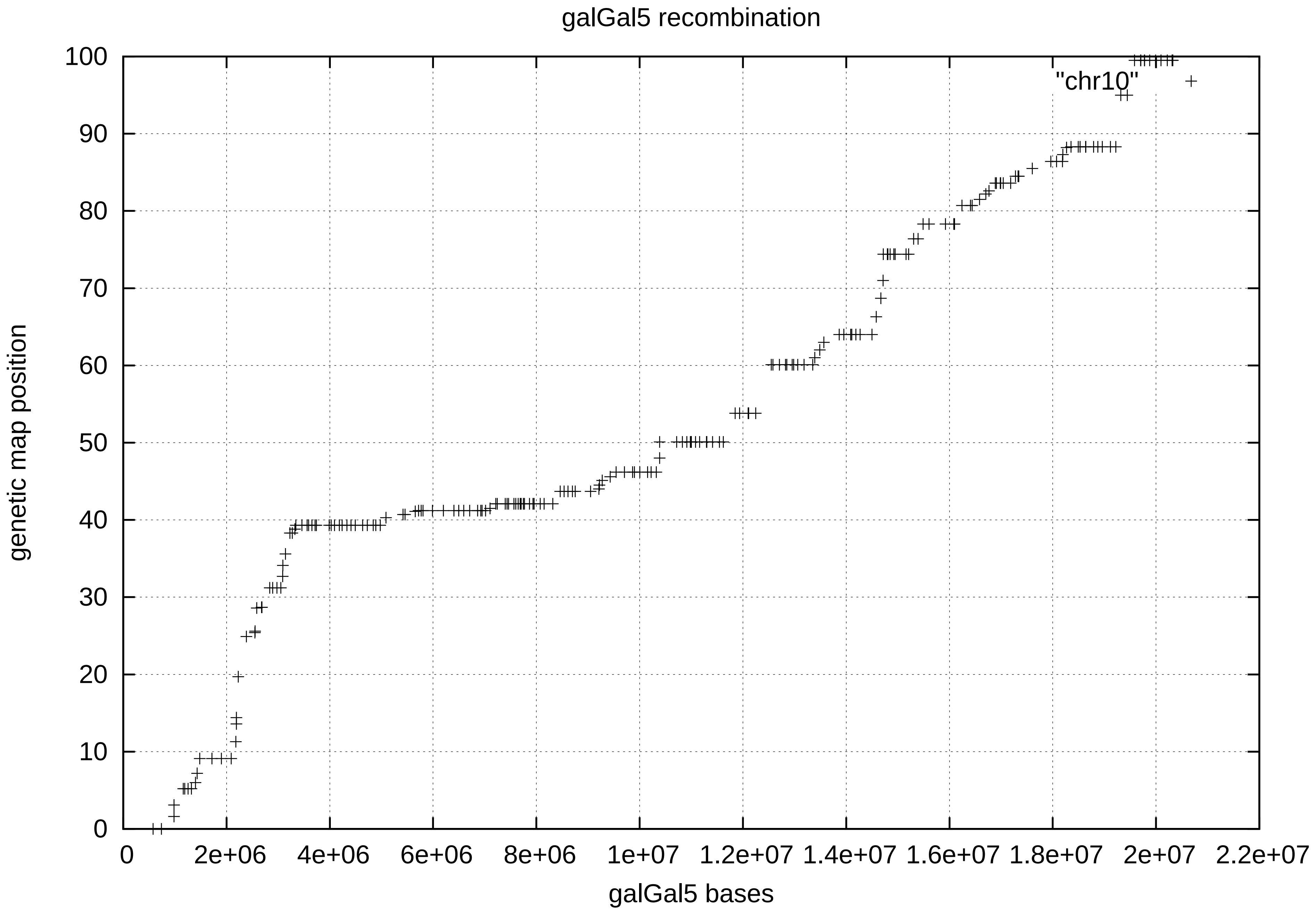
galGal5 recombination
"chr10"
genetic map position
galGal5 bases
0
10
20
30
40
50
60
70
80
90
100
0
2e+06
4e+06
6e+06
8e+06
1e+07
1.2e+07
1.4e+07
1.6e+07
1.8e+07
2e+07
2.2e+07

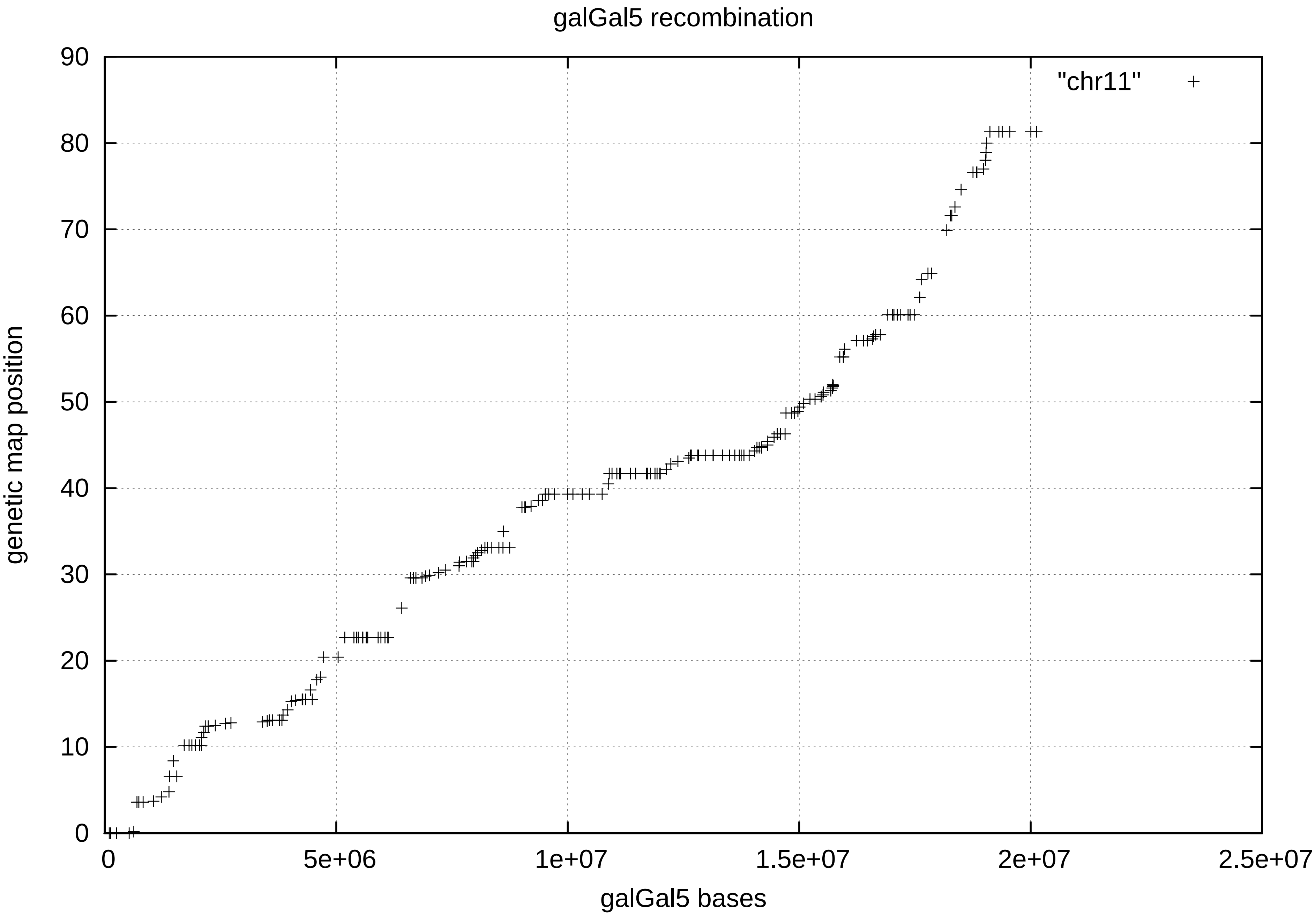
galGal5 recombination
"chr11"
genetic map position
galGal5 bases
0
10
20
30
40
50
60
70
80
90
0
5e+06
1e+07
1.5e+07
2e+07
2.5e+07

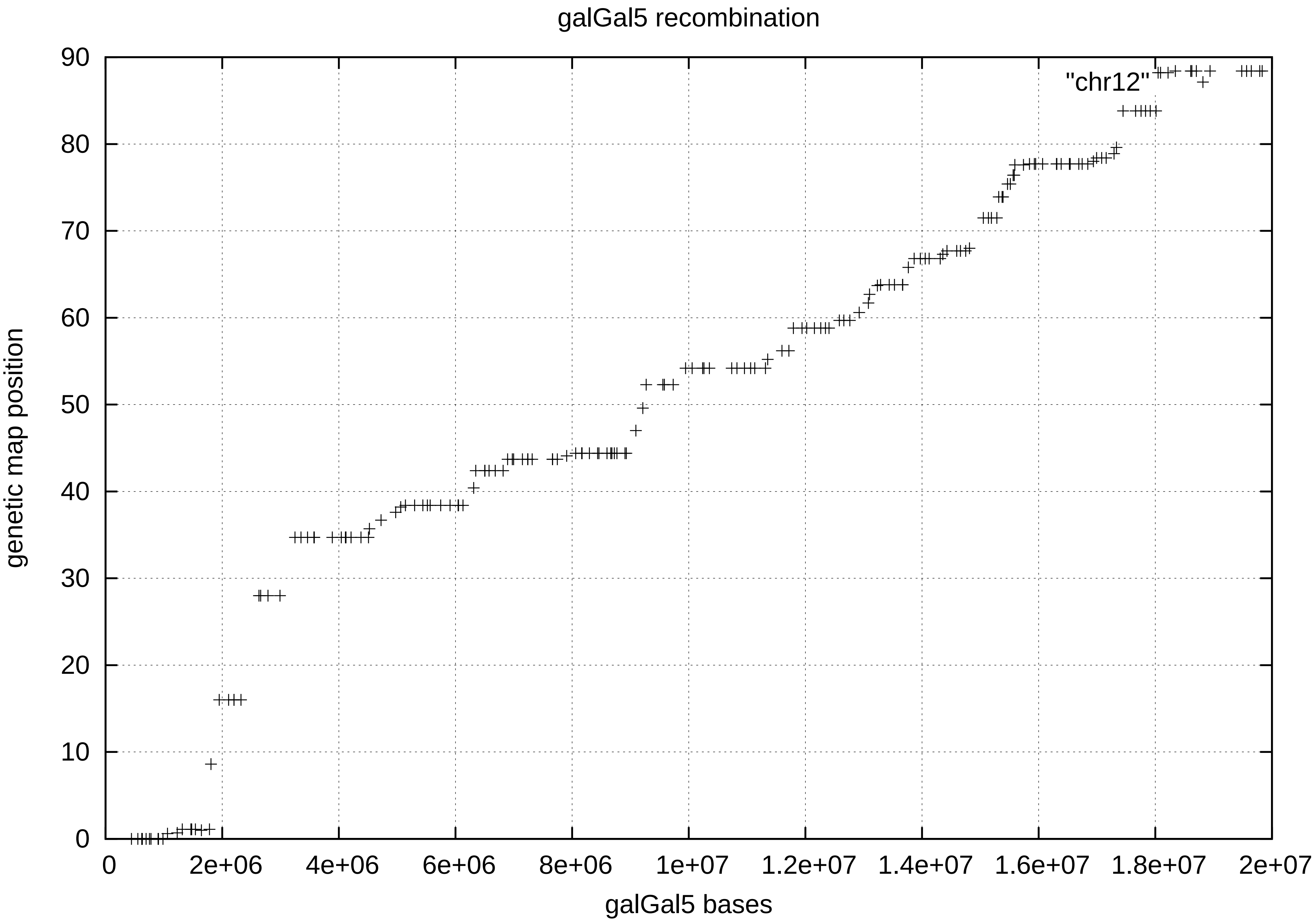

galGal5 recombination
"chr12"
genetic map position
galGal5 bases
0
10
20
30
40
50
60
70
80
90
2e+06
4e+06
6e+06
8e+06
1e+07
1.2e+07
1.4e+07
1.6e+07
1.8e+07
2e+07

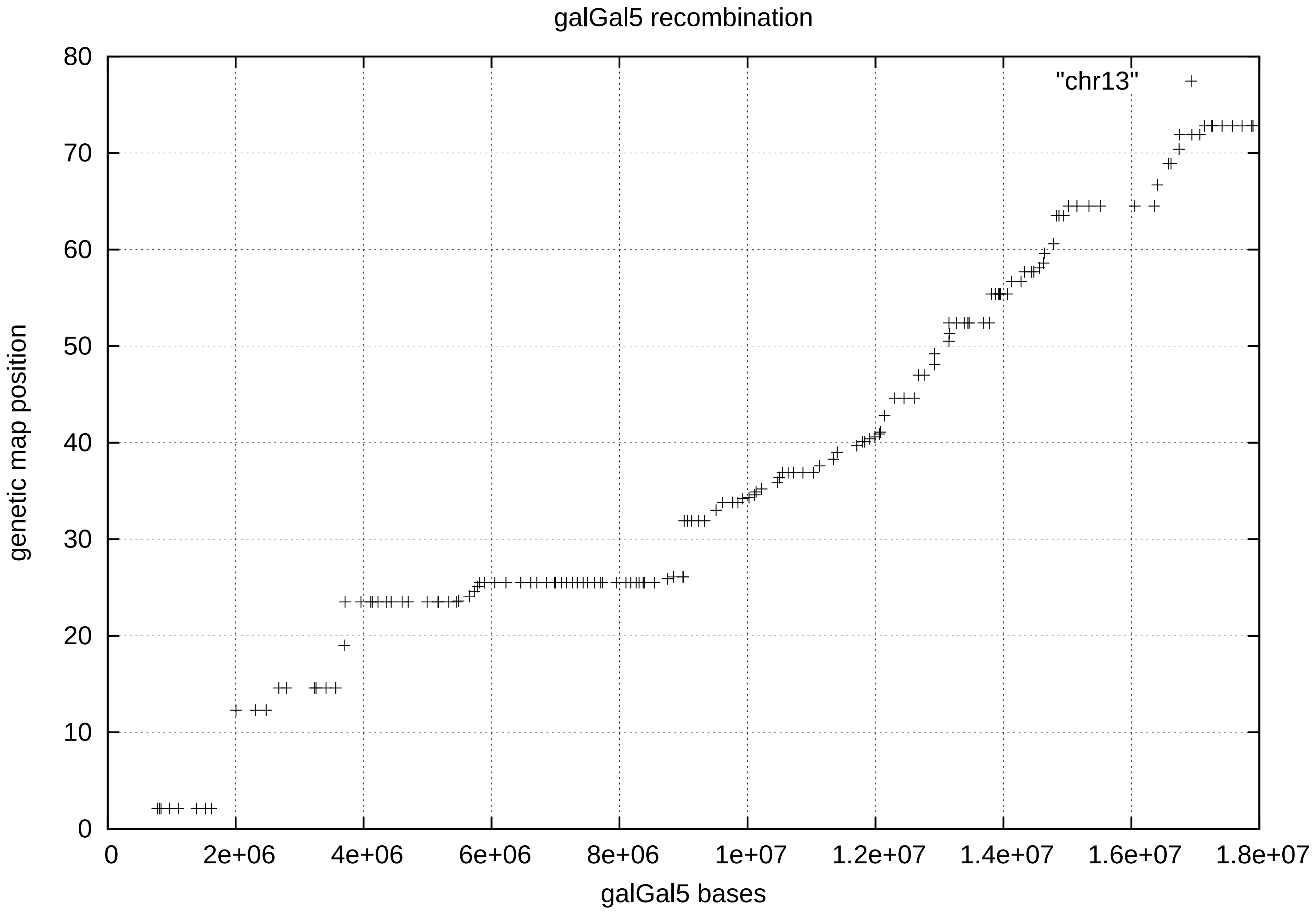

galGal5 recombination
"chr13"
genetic map position
galGal5 bases
0
10
20
30
40
50
60
70
80
0
2e+06
4e+06
6e+06
8e+06
1e+07
1.2e+07
1.4e+07
1.6e+07
1.8e+07

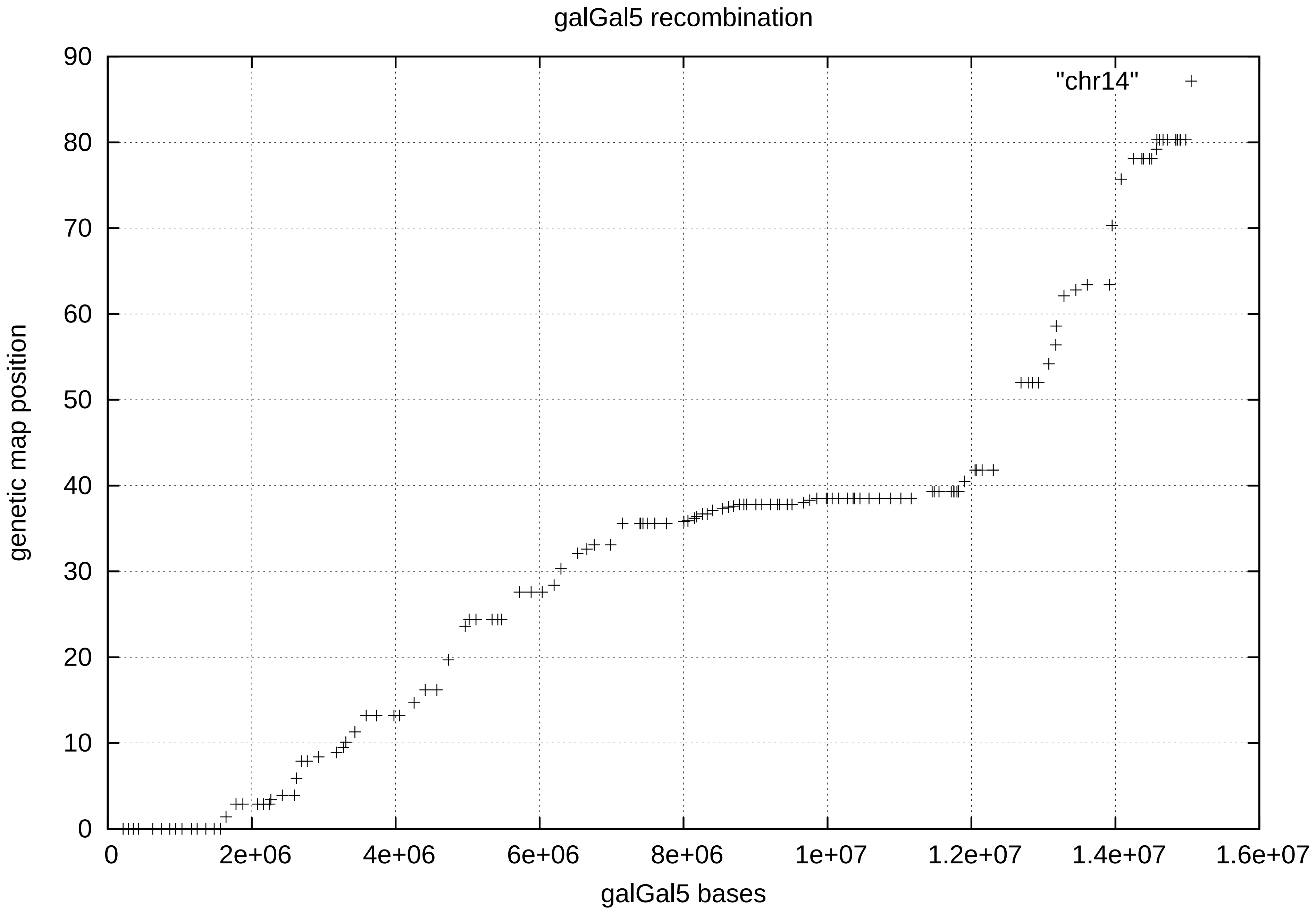
galGal5 recombination
"chr14"
genetic map position
galGal5 bases
0
10
20
30
40
50
60
70
80
90
0
2e+06
4e+06
6e+06
8e+06
1e+07
1.2e+07
1.4e+07
1.6e+07

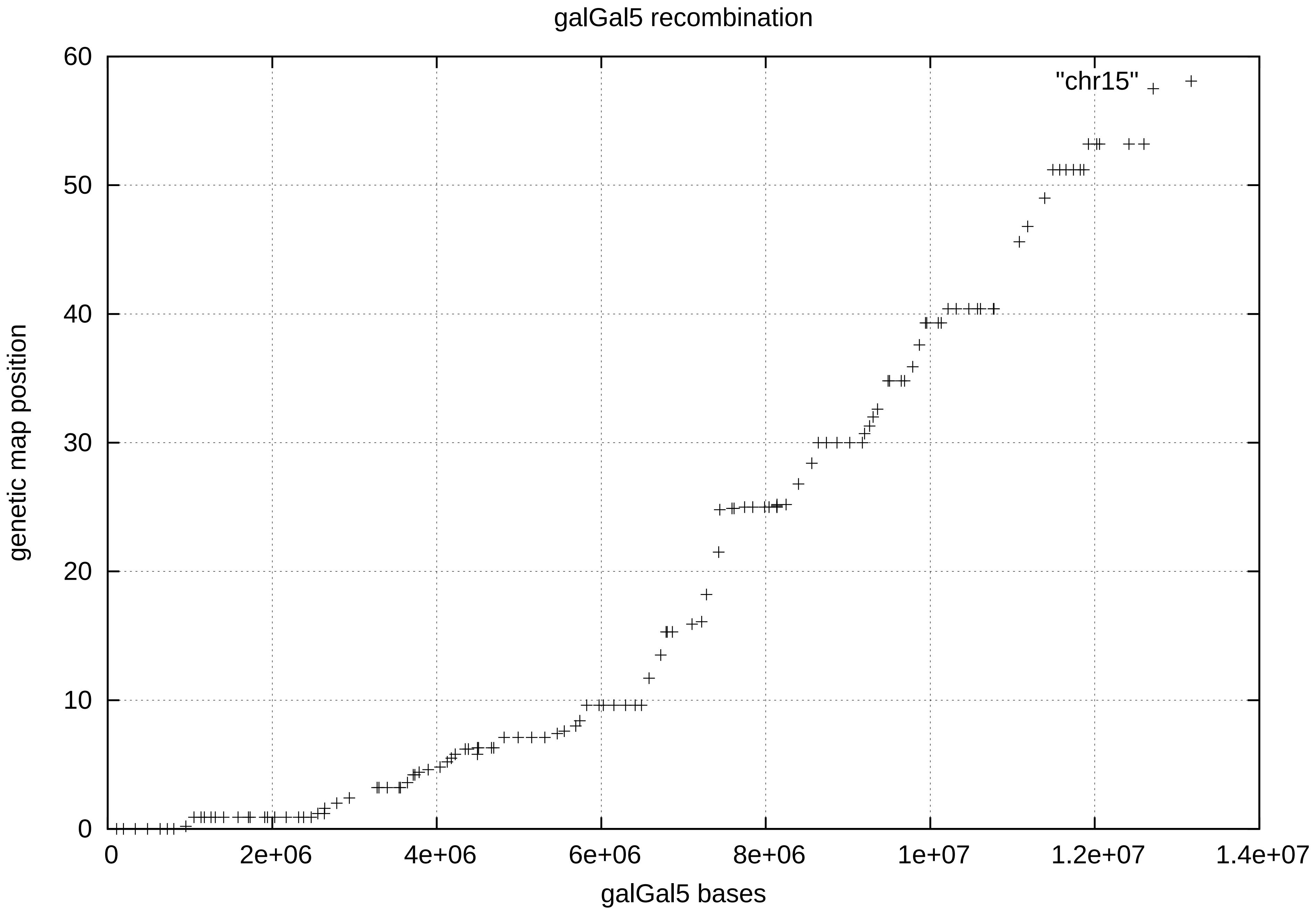
galGal5 recombination
"chr15"
genetic map position
0
10
20
30
40
50
60
0
2e+06
4e+06
6e+06
8e+06
1e+07
1.2e+07
1.4e+07
galGal5 bases

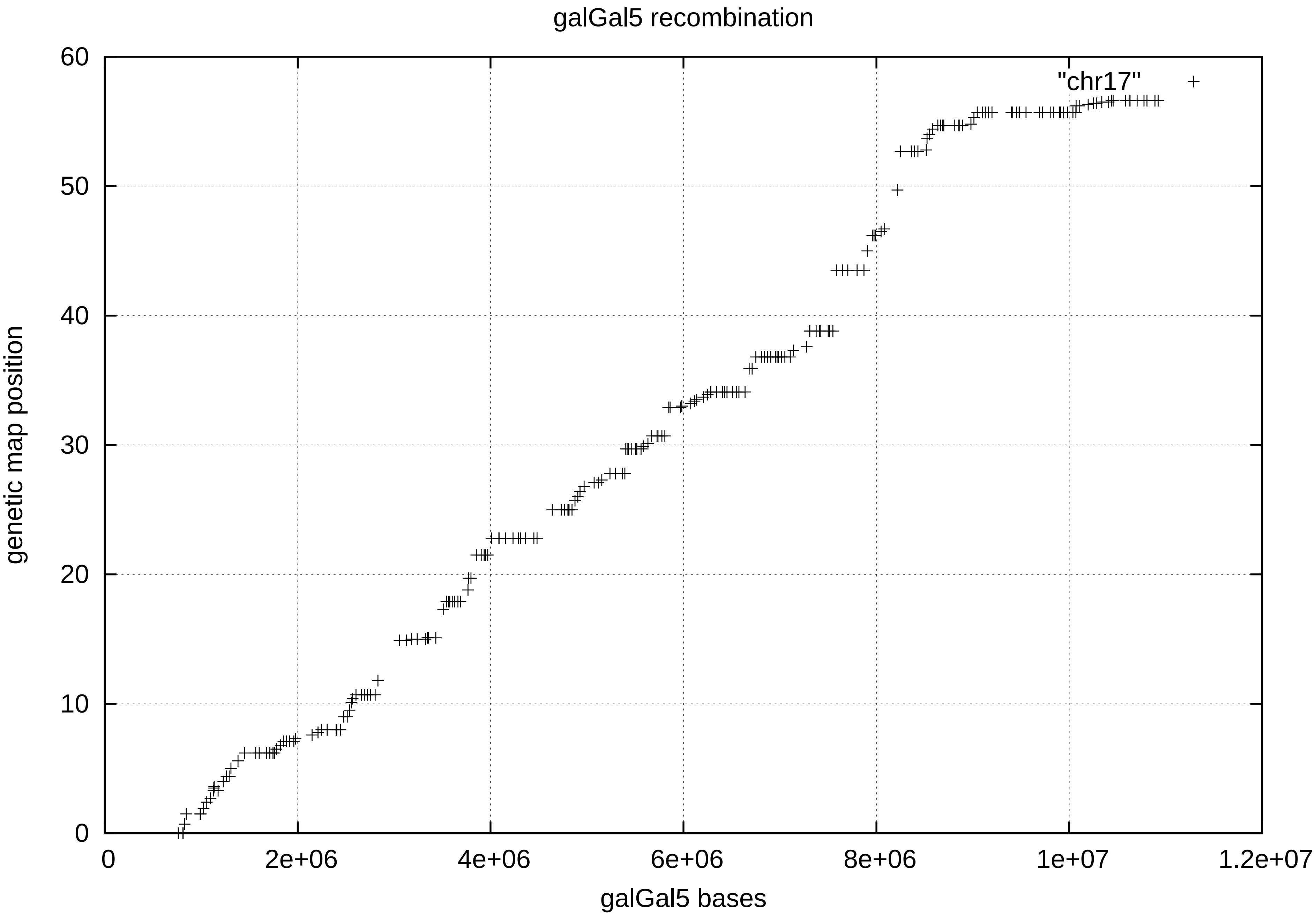

galGal5 recombination
genetic map position
galGal5 bases
"chr17"
0
10
20
30
40
50
60
0
2e+06
4e+06
6e+06
8e+06
1e+07
1.2e+07

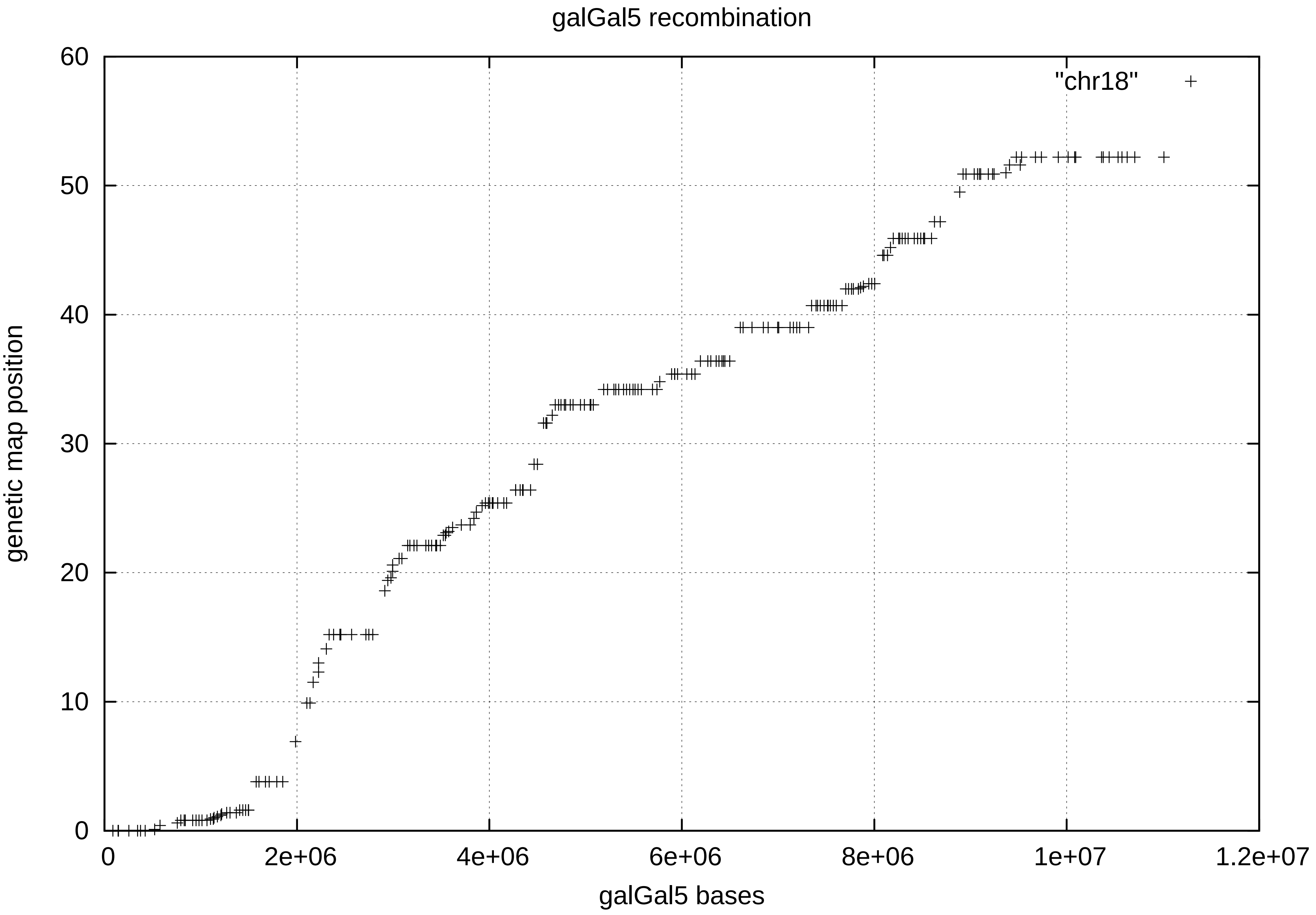
galGal5 recombination
"chr18"
genetic map position
galGal5 bases
0
10
20
30
40
50
60
0
2e+06
4e+06
6e+06
8e+06
1e+07
1.2e+07

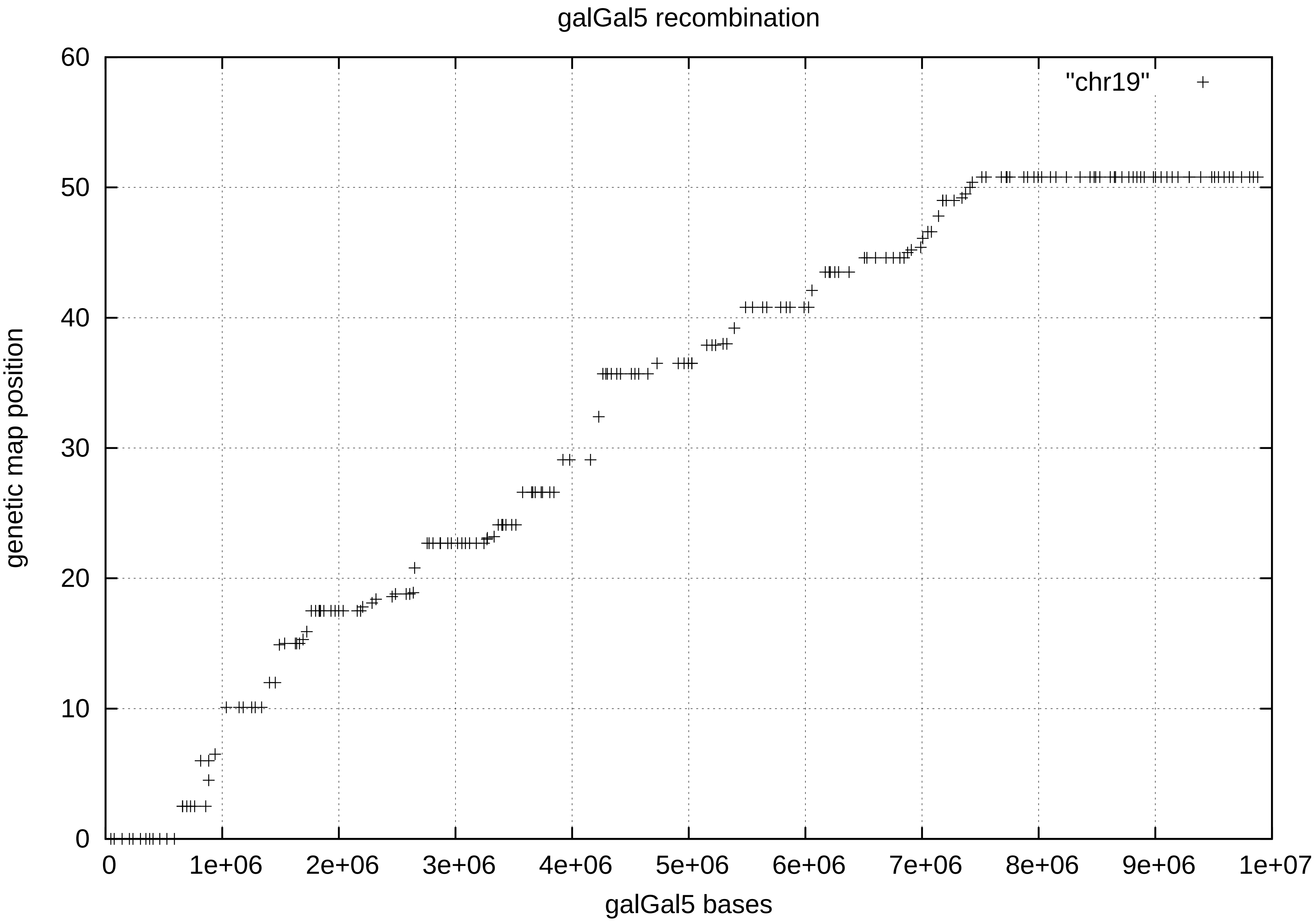

galGal5 recombination
"chr19"
genetic map position
galGal5 bases
0
10
20
30
40
50
60
0
1e+06
2e+06
3e+06
4e+06
5e+06
6e+06
7e+06
8e+06
9e+06
1e+07

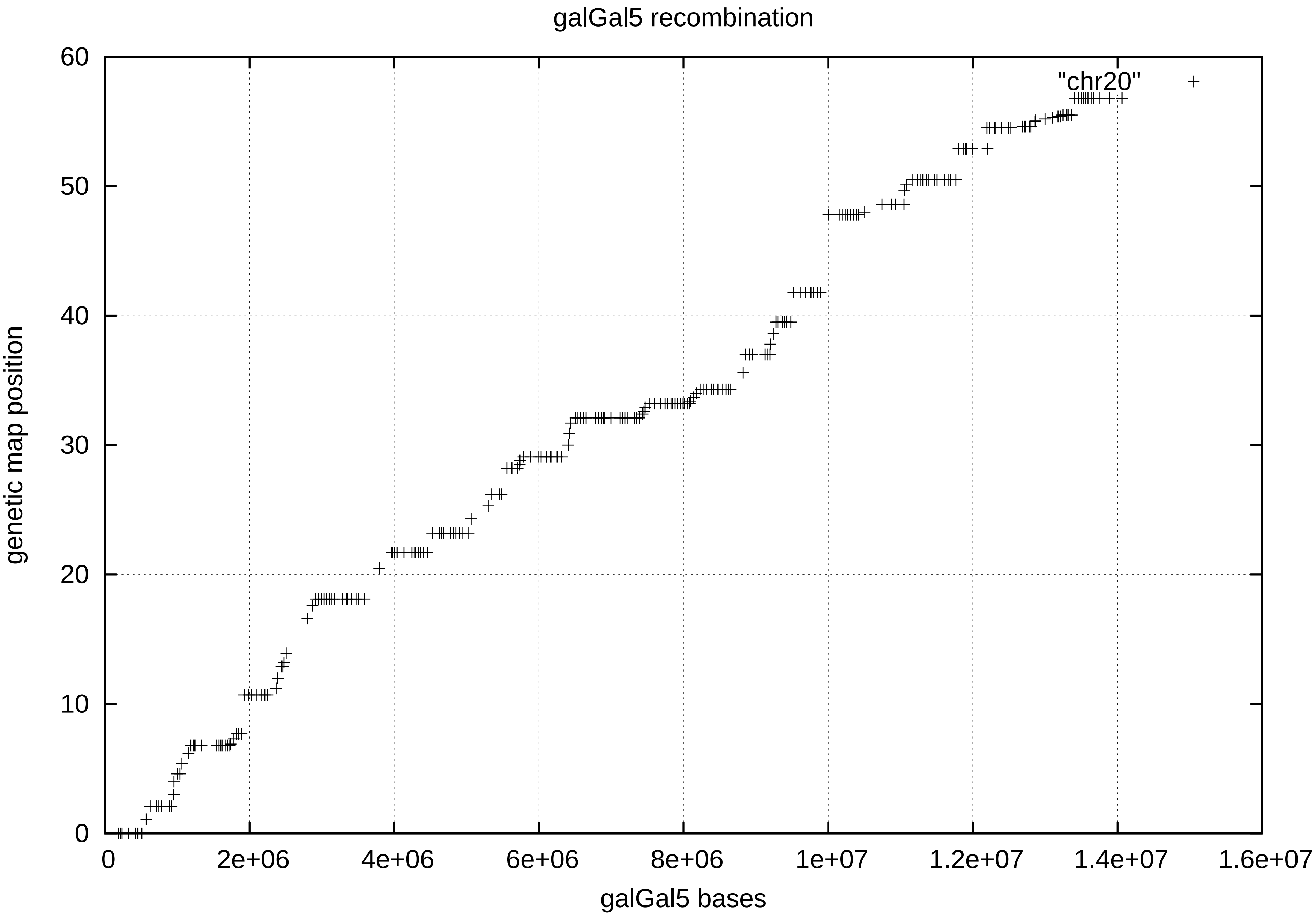
galGal5 recombination
"chr20"
genetic map position
galGal5 bases
0
10
20
30
40
50
60
0
2e+06
4e+06
6e+06
8e+06
1e+07
1.2e+07
1.4e+07
1.6e+07

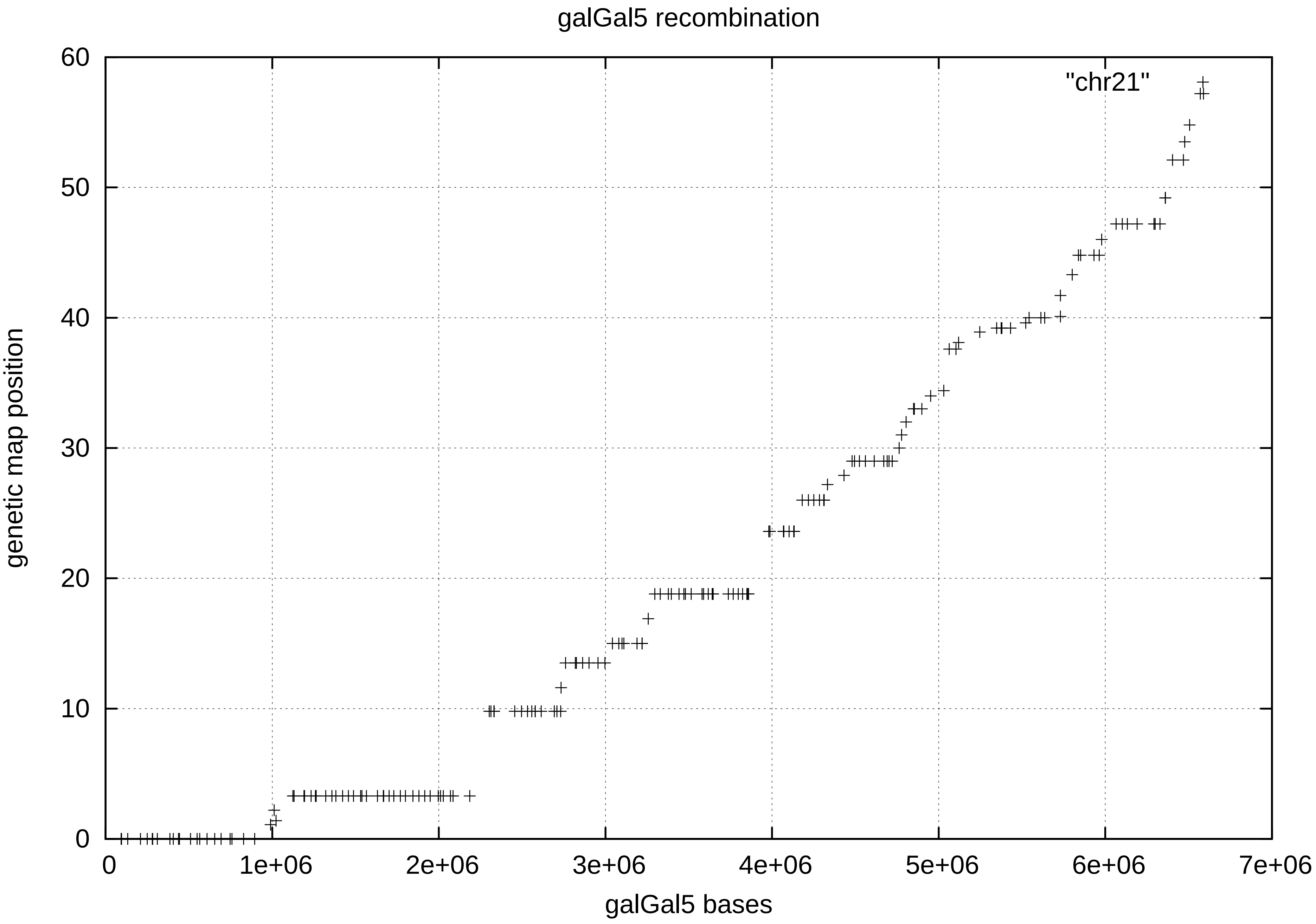

galGal5 recombination
"chr21"
genetic map position
galGal5 bases
0
10
20
30
40
50
60
0
1e+06
2e+06
3e+06
4e+06
5e+06
6e+06
7e+06

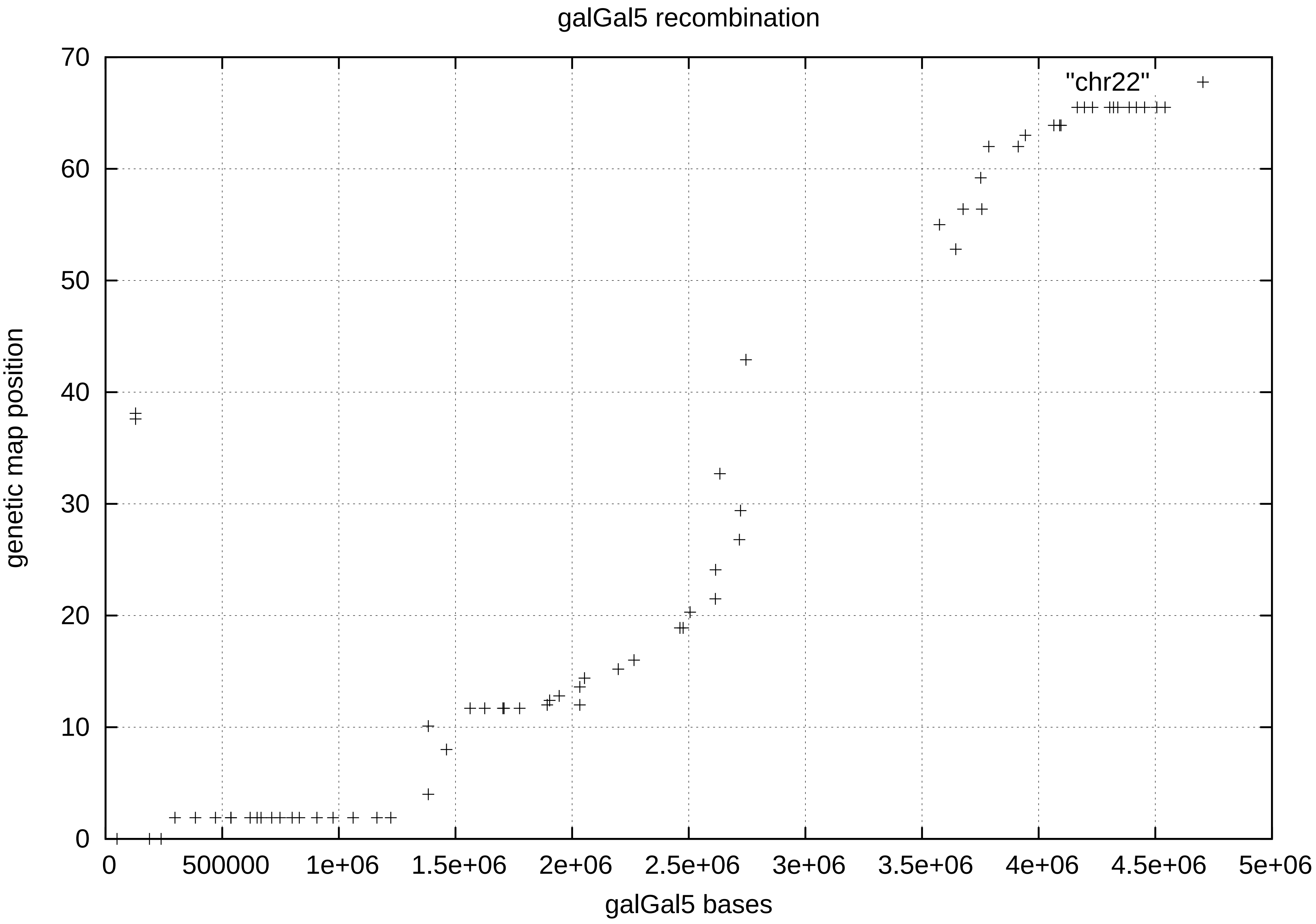

galGal5 recombination
genetic map position
galGal5 bases
"chr22"
0
10
20
30
40
50
60
70
0
500000
1e+06
1.5e+06
2e+06
2.5e+06
3e+06
3.5e+06
4e+06
4.5e+06
5e+06

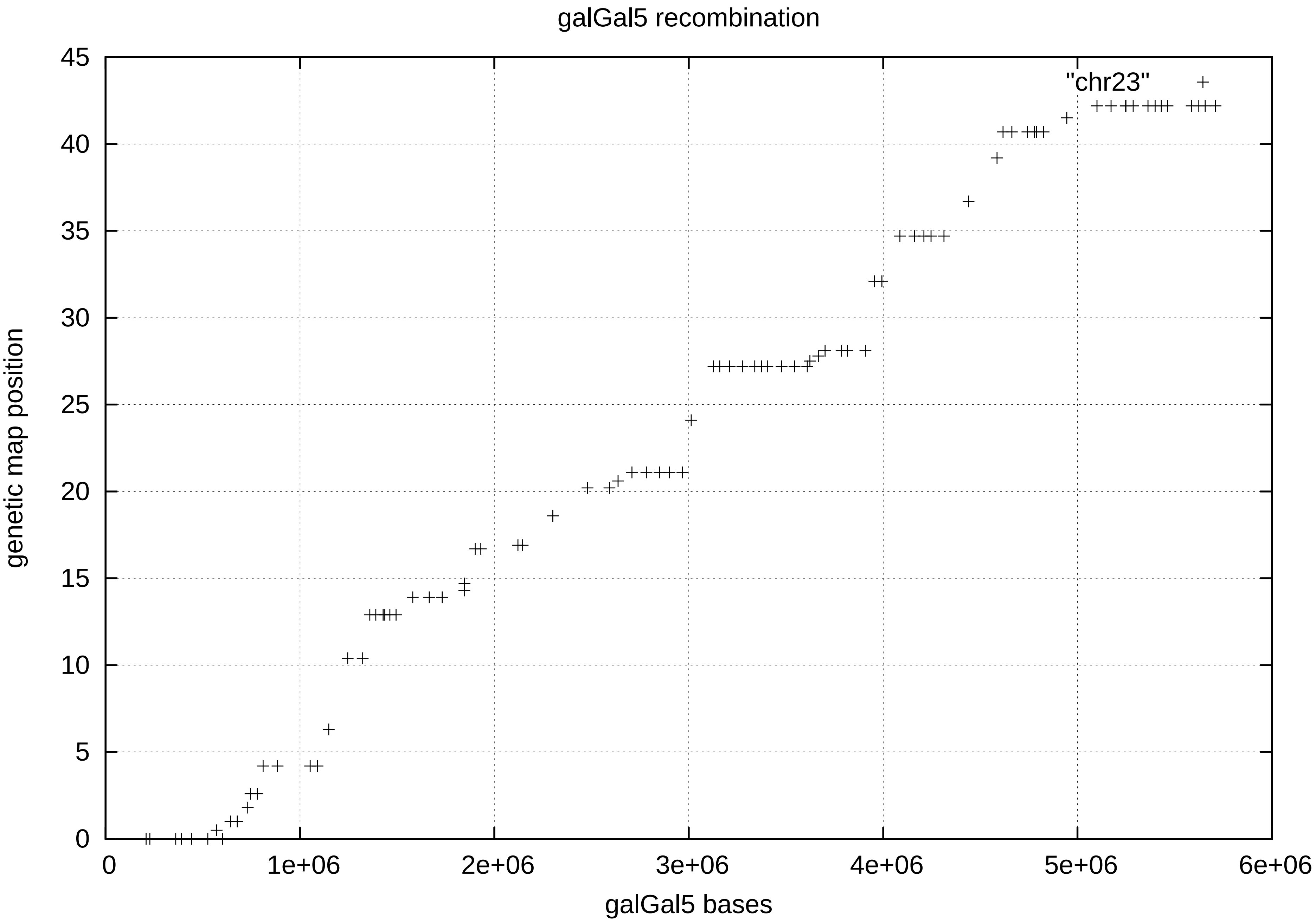

galGal5 recombination
"chr23"
genetic map position
galGal5 bases
0
5
10
15
20
25
30
35
40
45
0
1e+06
2e+06
3e+06
4e+06
5e+06
6e+06

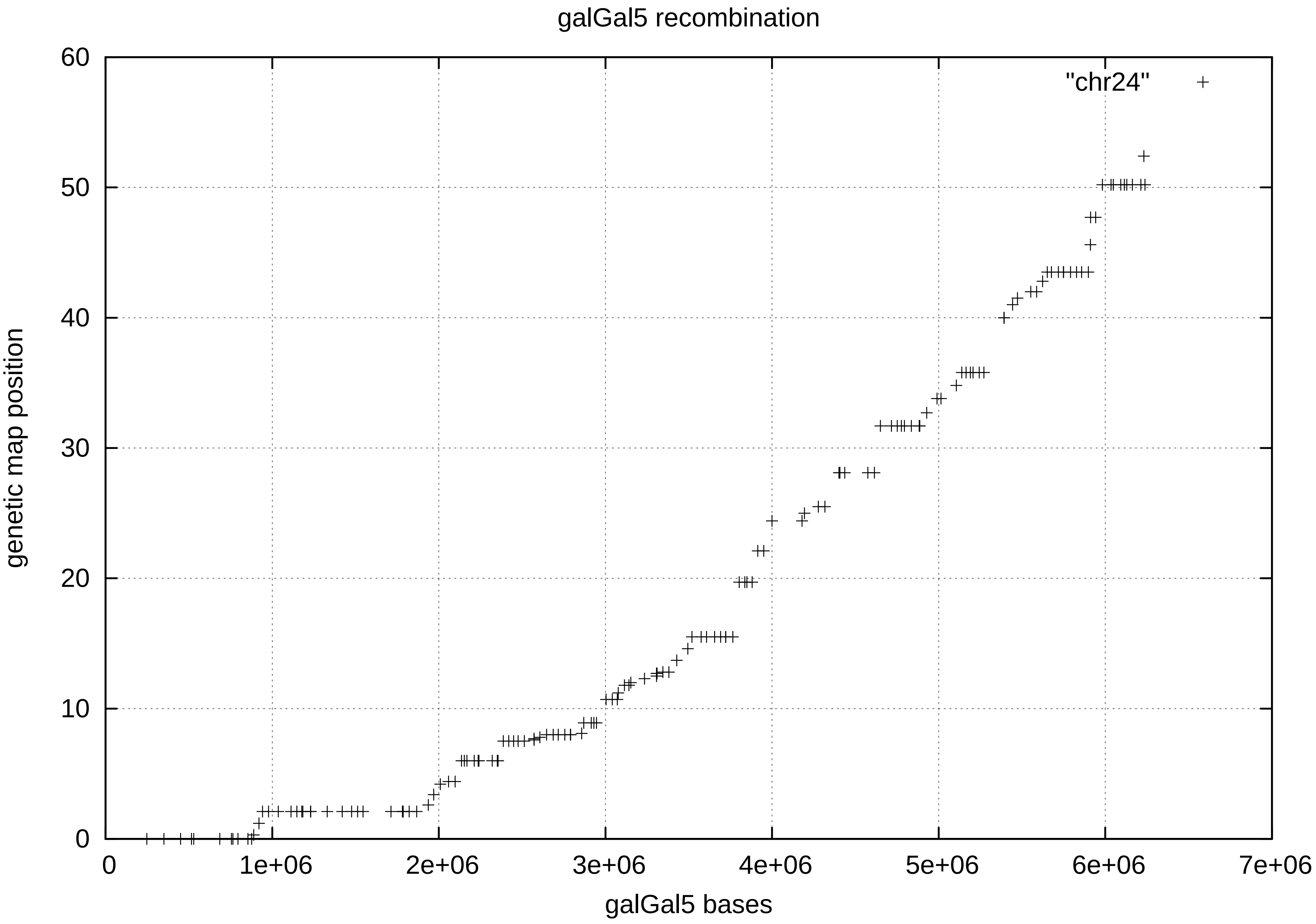
galGal5 recombination
"chr24"
genetic map position
galGal5 bases
0
10
20
30
40
50
60
0
1e+06
2e+06
3e+06
4e+06
5e+06
6e+06
7e+06

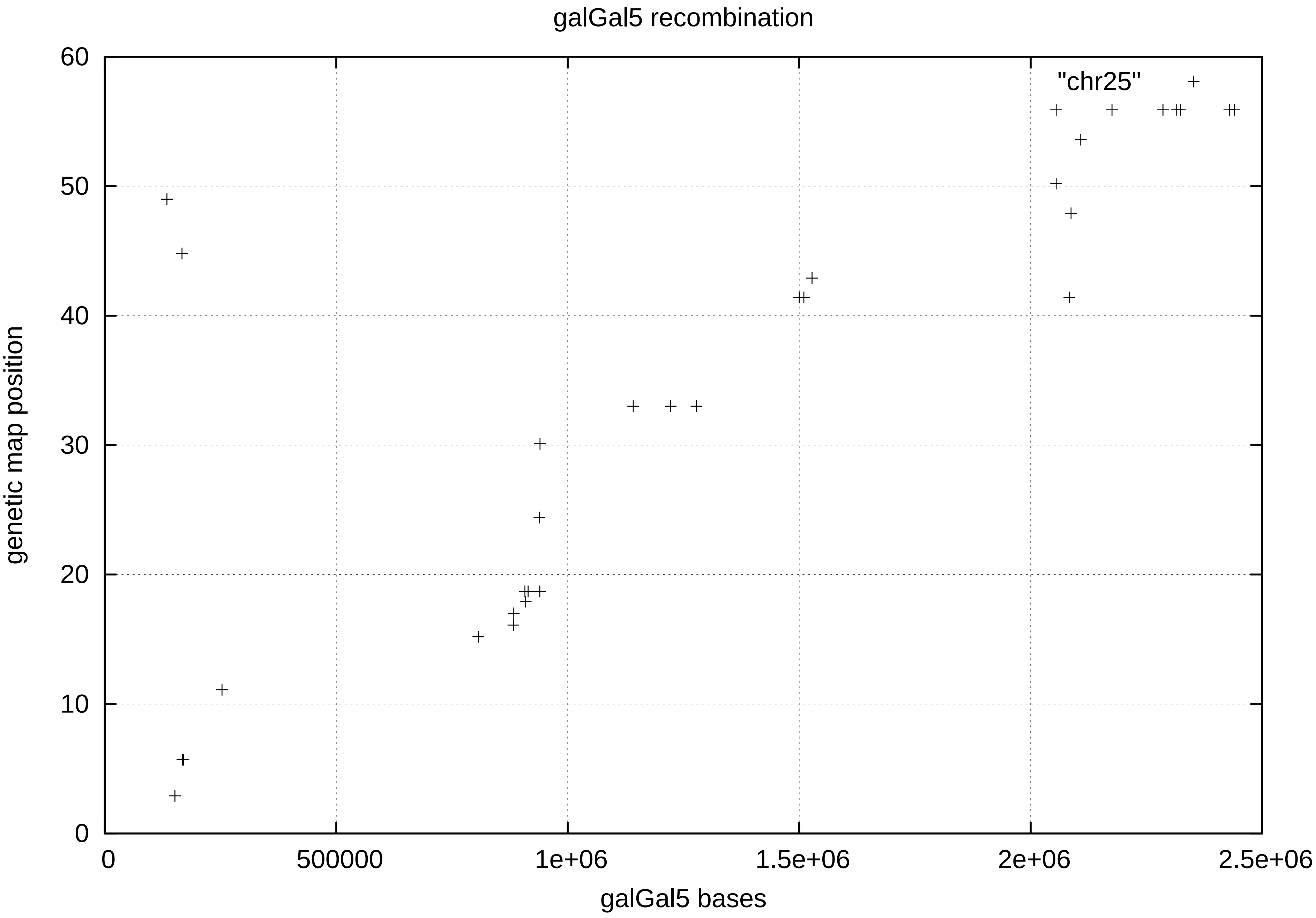
galGal5 recombination
"chr25"
genetic map position
galGal5 bases
0
10
20
30
40
50
60
0
500000
1e+06
1.5e+06
2e+06
2.5e+06

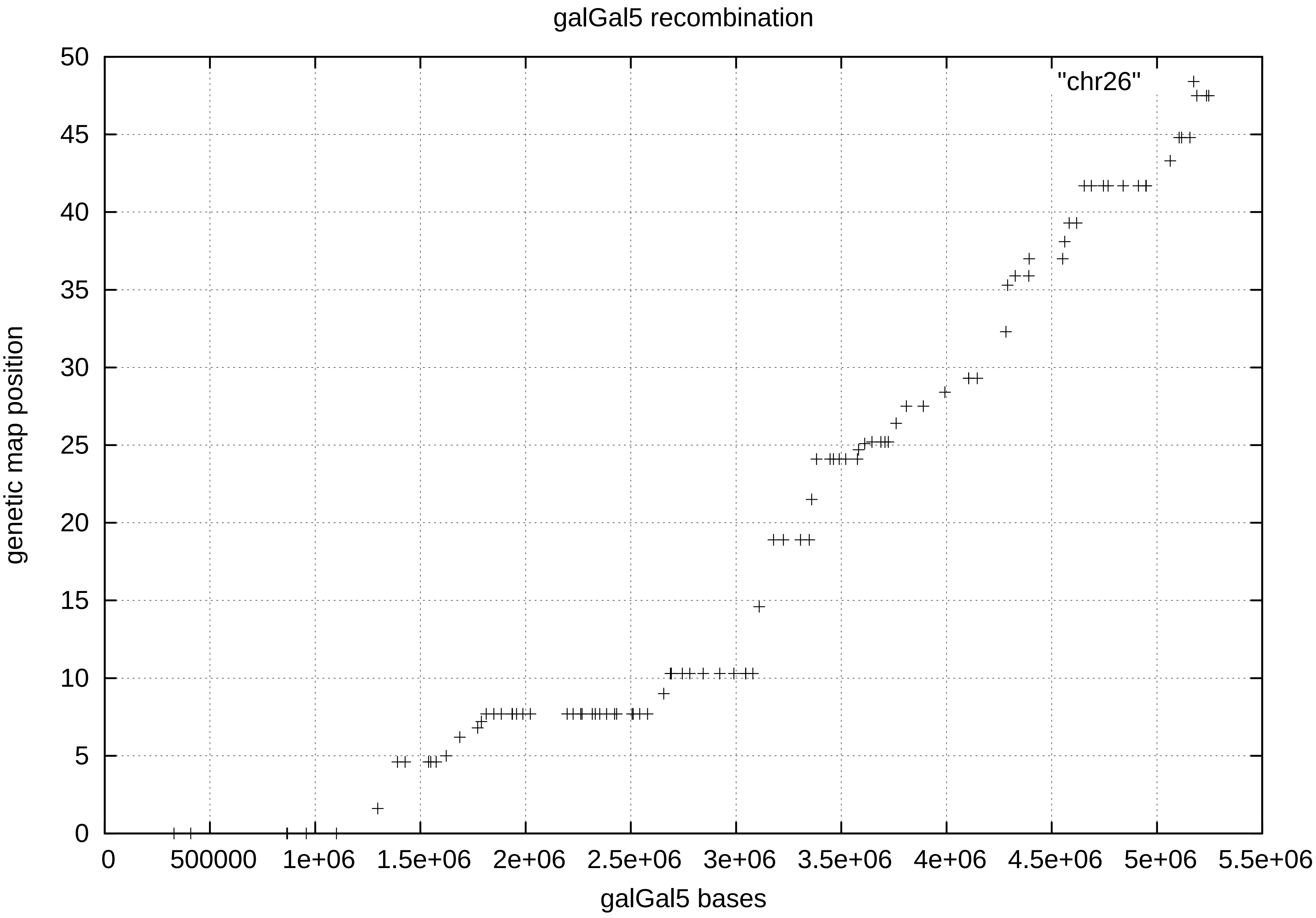

galGal5 recombination
"chr26"
genetic map position
galGal5 bases
0
5
10
15
20
25
30
35
40
45
50
0
500000
1e+06
1.5e+06
2e+06
2.5e+06
3e+06
3.5e+06
4e+06
4.5e+06
5e+06
5.5e+06

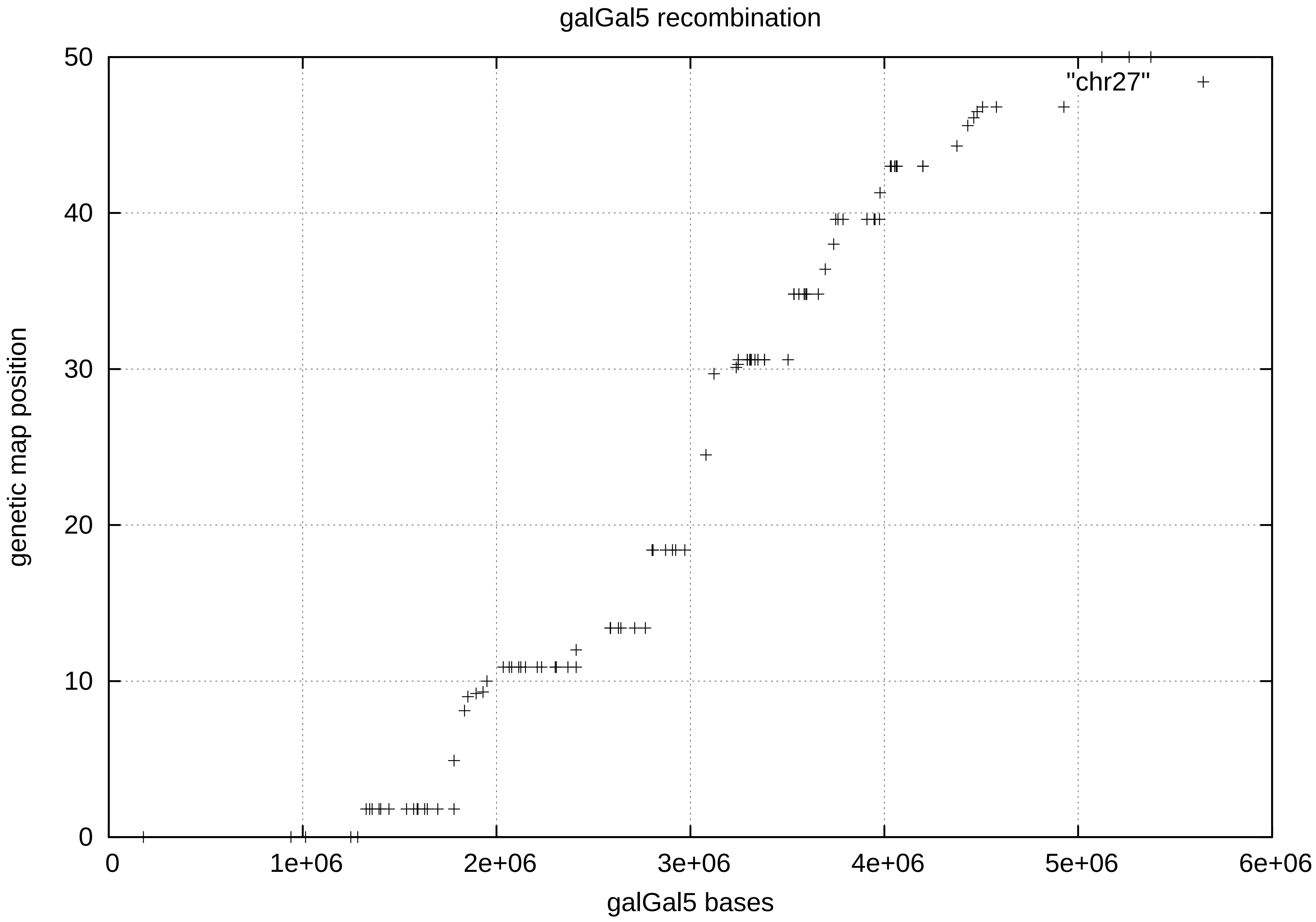
galGal5 recombination
"chr27"
genetic map position
galGal5 bases
0
10
20
30
40
50
0
1e+06
2e+06
3e+06
4e+06
5e+06
6e+06

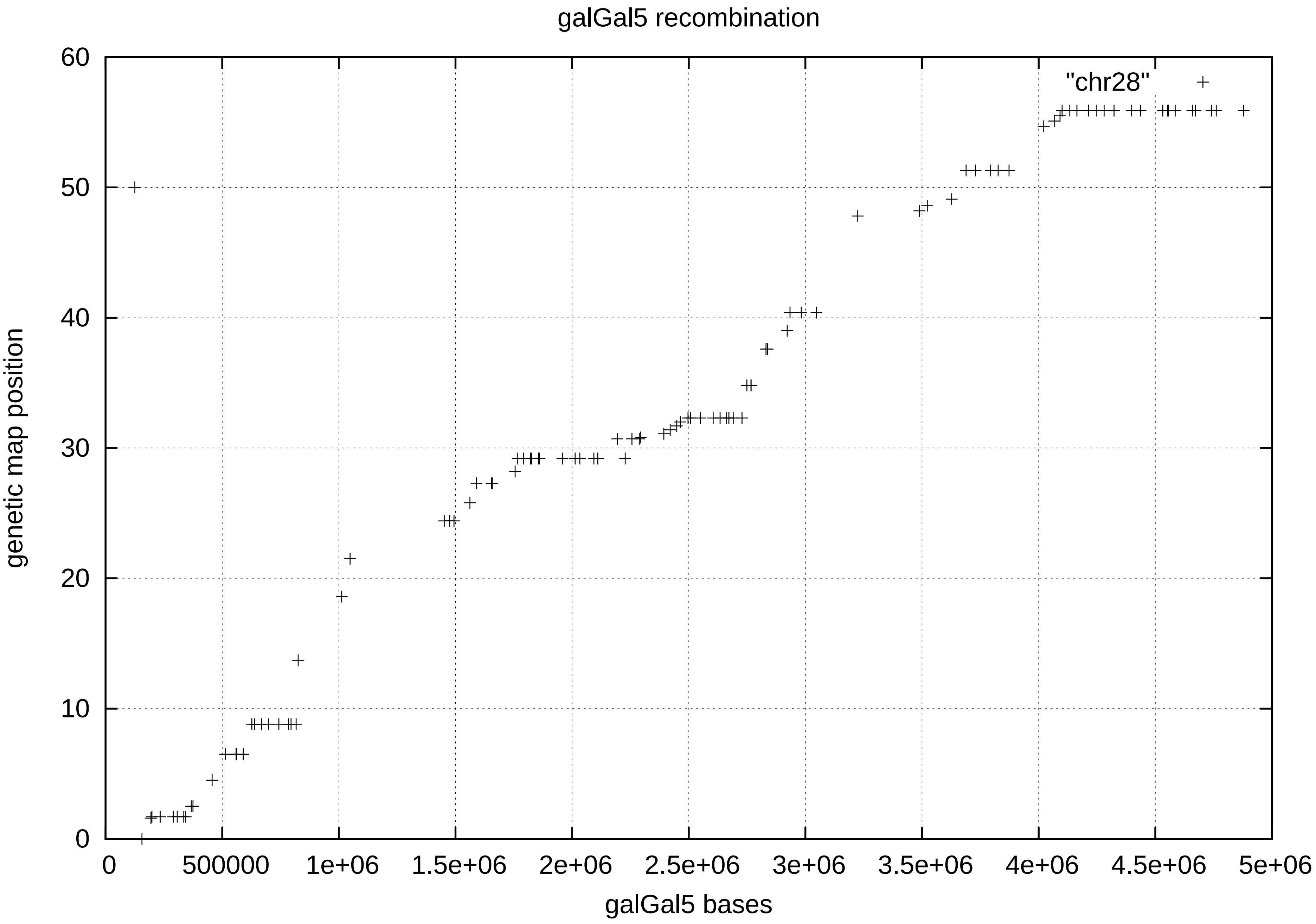

galGal5 recombination
"chr28"
genetic map position
galGal5 bases
0
10
20
30
40
50
60
0
500000
1e+06
1.5e+06
2e+06
2.5e+06
3e+06
3.5e+06
4e+06
4.5e+06
5e+06
