# Supplementary material for: A New Chicken Genome Assembly Provides Insight into Avian Genome Structure
Source: G3 (Bethesda). 2016 Nov 14;7(1):109–17. doi: 10.1534/g3.116.035923 (PMC5217101; doi:10.1534/g3.116.035923)
Supplement: Supplementary file 10 [file 109FileS4.docx]

File S4. The most common conserved domains among all categories of novel genes. (.xls, 381 KB)

<http://www.g3journal.org/lookup/suppl/doi:10.1534/g3.116.035923/-/DC1/FileS4.xls>
