# Supplementary material for: A New Chicken Genome Assembly Provides Insight into Avian Genome Structure
Source: G3 (Bethesda). 2016 Nov 14;7(1):109–17. doi: 10.1534/g3.116.035923 (PMC5217101; doi:10.1534/g3.116.035923)
Supplement: Supplementary file 12 [file 109FileS5.docx]

File S5. InterProScan (Mitchell et al. 2015) assignments to unique gene ontology (GO) annotation based on functional domain conservation. (.xls, 482 KB)

[http://www.g3journal.org/lookup/suppl/doi:10.1534/g3.116.035923/-/DC1/FileS5.xls](http://www.g3journal.org/lookup/suppl/doi:10.1534/g3.116.035923/-/DC1/FileS4.xls)
