# Supplementary material for: A New Chicken Genome Assembly Provides Insight into Avian Genome Structure
Source: G3 (Bethesda). 2016 Nov 14;7(1):109–17. doi: 10.1534/g3.116.035923 (PMC5217101; doi:10.1534/g3.116.035923)
Supplement: Supplementary file 20 [file 109FileS9.pdf]

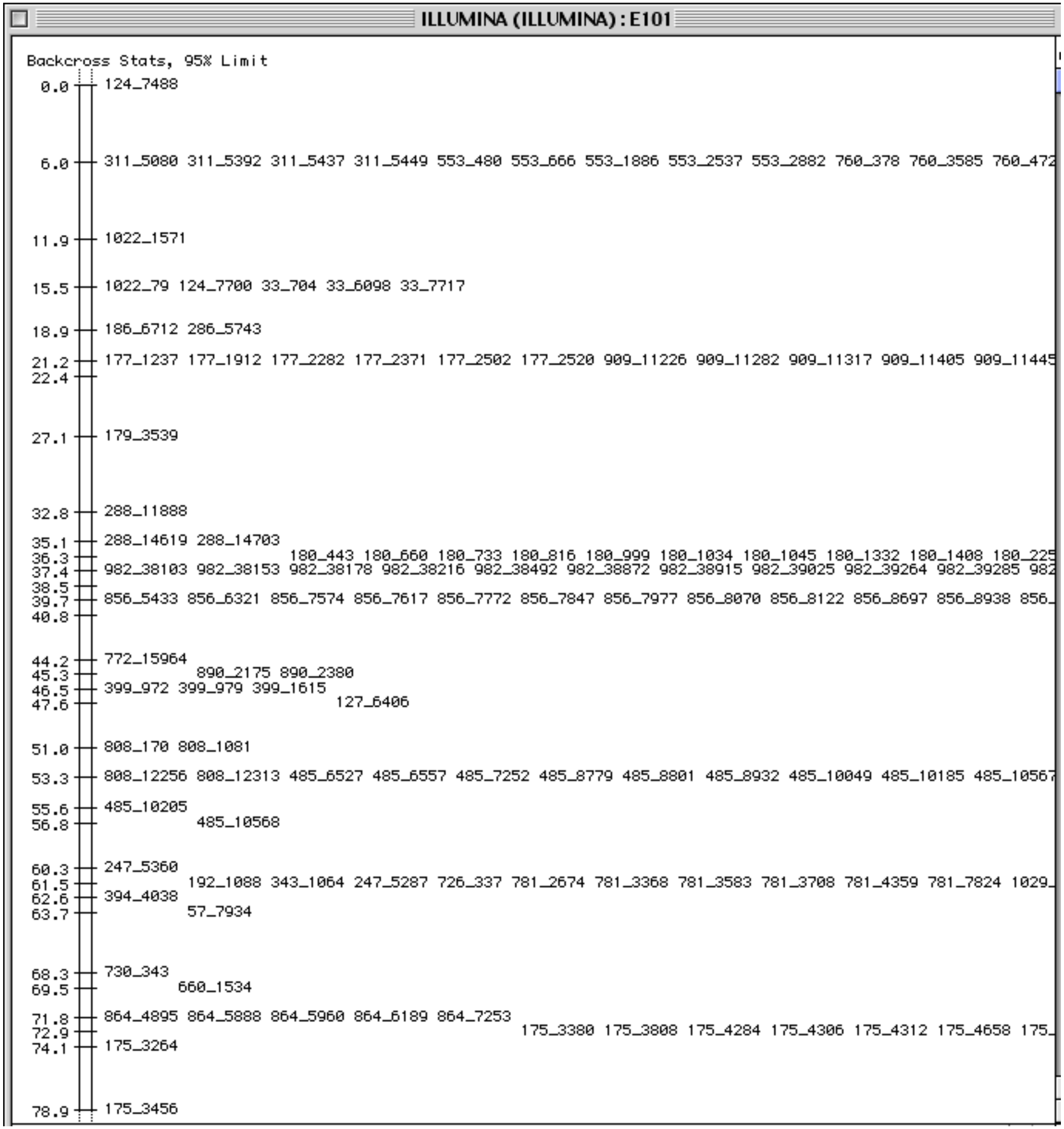
ILLUMINA (ILLUMINA) : E101
Backcross Stats, 95% Limit
0.0 124_7488
6.0 311_5080 311_5392 311_5437 311_5449 553_480 553_666 553_1886 553_2537 553_2882 760_378 760_3585 760_472
11.9 1022_1571
15.5 1022_79 124_7700 33_704 33_6098 33_7717
18.9 186_6712 286_5743
21.2 177_1237 177_1912 177_2282 177_2371 177_2502 177_2520 909_11226 909_11282 909_11317 909_11405 909_11445
22.4
27.1 179_3539
32.8 288_11888
35.1 288_14619 288_14703
36.3 180_443 180_660 180_733 180_816 180_999 180_1034 180_1045 180_1332 180_1408 180_225
37.4 982_38103 982_38153 982_38178 982_38216 982_38492 982_38872 982_38915 982_39025 982_39264 982_39285 982
38.5
39.7 856_5433 856_6321 856_7574 856_7617 856_7772 856_7847 856_7977 856_8070 856_8122 856_8697 856_8938 856_
40.8
44.2 772_15964
45.3 890_2175 890_2380
46.5 399_972 399_979 399_1615
47.6 127_6406
51.0 808_170 808_1081
53.3 808_12256 808_12313 485_6527 485_6557 485_7252 485_8779 485_8801 485_8932 485_10049 485_10185 485_10567
55.6 485_10205
56.8 485_10568
60.3 247_5360
61.5 192_1088 343_1064 247_5287 726_337 781_2674 781_3368 781_3583 781_3708 781_4359 781_7824 1029_
62.6 394_4038
63.7 57_7934
68.3 730_343
69.5 660_1534
71.8 864_4895 864_5888 864_5960 864_6189 864_7253
72.9 175_3380 175_3808 175_4284 175_4306 175_4312 175_4658 175_
74.1 175_3264
78.9 175_3456

ILLUMINA (ILLUMINA) : E102

Backcross Stats, 95% Limit

0.0 40_18861 40_19543 40_20800 40_22216 420_2449 727_11381 727_11469 727_11518 727_14866 742_16017 742_16135 742_16326 742_16705 89
2.3 452_408
3.4 536_1557 801_7889 801_12551
11.6 223_1256 397_3024 571_1953 571_1987 571_2581 571_2692
12.7 421_959
13.8 999_6422
15.0 903_11712 903_11715 793_3096 793_6563 793_6769
16.1 793_11184 793_11233 795_1227 795_1503 795_1533 795_3001 795_5117 795_5159 795_5312 795_5349 795_5682 795_5812 795_6829 795_6891
17.3 199_11343
18.4 218_6628
19.6 199_13275
20.7 199_12474 199_12616 199_12697 199_12916 199_13230 199_17030 199_19248 199_19407 199_21205 199_21506 199_22581 199_23776 199_238
21.9 199_12939
23.1 986_9904 986_12709 986_14745 986_14867 986_19628 986_19904 986_19934 986_19971 986_21283 986_21567 986_24129 986_24345 986_2434
24.3
34.5 529_1401 529_1491 529_1519 529_2126 529_2937
37.9 315_10459 315_18992
40.2 155_4391
41.3 924_8071
44.7 778_9791
47.0 594_19948
48.1 594_6511 594_12141 594_19030 594_19104 594_19660 594_19760 594_19769 594_19880 594_20007 898_5760 898_5867 898_6623 898_6894 89
50.4 87_25033
51.5 87_1495 87_16355 87_16474 87_21689 87_21844 87_23735 87_24922 87_25056 87_25130 87_25139 87_28961
53.8 904_1117 904_1137 904_1177 904_1201
57.2 391_2606 391_7640 391_19891 810_74305 810_74640 810_76357 810_82866 810_83566 810_85179 810_86368 810_86566 810_86451 810_86492
58.3 810_66238
59.5 810_12788 810_12945 810_13163 810_13246 810_13738 810_15122 810_20682 810_23330 810_24606 810_26728 810_26953 810_277
67.5 152_4869 152_5106 152_5150
68.7 43_6466
69.8 761_15269
71.0 992_8313

ILLUMINA (ILLUMINA) : E103

Backcross Stats, 95% Limit

0.0 972_884
12.8 424_475 424_476 424_633 424_668 424_685 424_719 424_820 424_848 424_1061
21.9 698_574
23.0 838_3894
24.2 374_2837 374_2931 374_3227 374_3231 374_3286
27.6 374_3253
31.0 374_2727
32.1 298_1735
33.2 295_658 295_1701 295_2225
35.5 68_7629 68_7641
38.9 110_5848
46.9 38_699 38_3555 38_3572 38_3652 38_3878 38_3888 38_4086 38_4211 38_4539
50.3 38_4456
57.4 144_4464 144_652
59.7 301_10853 301_13064 301_13268 301_15514 301_15574 301_15579 301_17091 301_17196 301_17200 301_17921 301_17984 301_18197 301_1[illegible]
66.6 334_342 334_1948 334_2377 334_2389 334_2607 334_3196 334_3231 334_3266 334_3357
67.8 109_7445 265_5356 473_3809

ILLUMINA (ILLUMINA) : E104

Backcross Stats, 95% Limit

0.0 — 712_767

2.3 — 86_4268

3.4 — 489_1324 41_3451 41_4060 41_4463 41_4558 41_5994 41_7383 41_7450 41_8754 41_8795 41_8808 41_8846 718_260 718_324 463_130 463_109

4.6 — 463_79

6.9 — 64_254 64_647 64_919 64_2284 64_2380 64_2586 64_2801 64_3565 64_3860 64_4121 64_4587 64_5290 64_5365 64_5425 64_5860 64_7339

8.0 — 64_8617 64_8729 64_8756 64_9131 64_10935 64_11012 64_11065 64_11147 64_11224 64_11290 64_11886 64_12391 64_12629 64_12643 64_127

9.2 — 895_1594 335_4515 895_7955 895_8058 895_8334 895_8341 895_8468 895_7350

ILLUMINA (ILLUMINA) : E105

Backcross Stats, 95% Limit

0.0 545_1633 545_1645 545_1766

5.7 194_5154 194_5699 194_6568 194_6835

9.1 98_2325 148_2361 148_2378 148_2443 148_2489 148_2672 148_2777 148_3707 148_3815 148_3917 148_3943 148_3976 148_4021 14

12.6 840_8764

13.8 363_493 363_526

16.1 564_9371 165_934

18.4 165_924

23.2 42_3755 42_3932 42_3940 42_4151 42_7587

24.3 353_4257

26.6 204_5646

31.3 204_4312

34.8 1001_813 1001_1023 1001_1131 1001_1162 1001_1170

39.3 443_1709 443_2847 688_154 688_942 688_1111 688_1263

66.4 662_451 987_9848

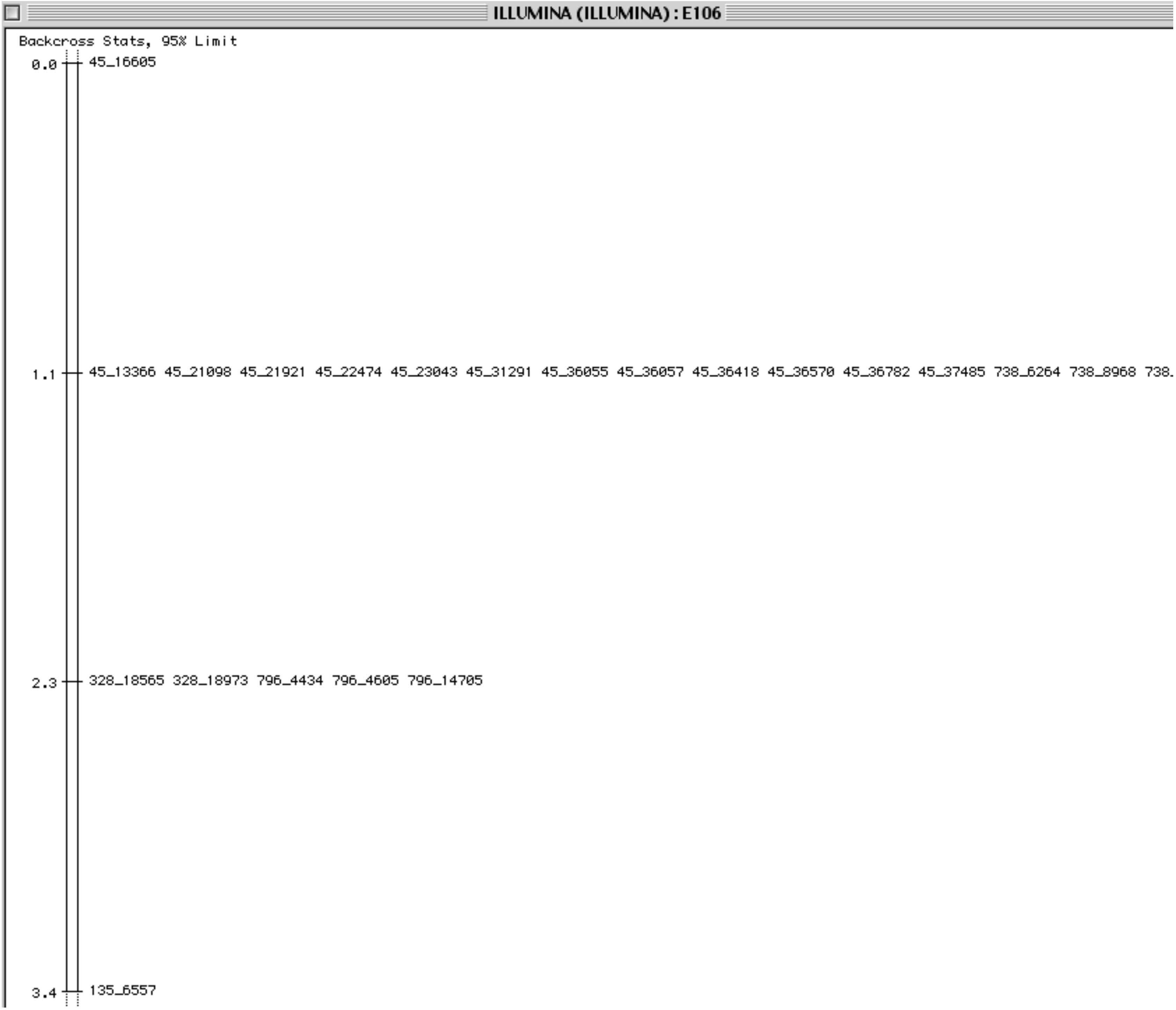

ILLUMINA (ILLUMINA) : E106
Backcross Stats, 95% Limit
0.0
45_16605
1.1
45_13366 45_21098 45_21921 45_22474 45_23043 45_31291 45_36055 45_36057 45_36418 45_36570 45_36782 45_37485 738_6264 738_8968 738.
2.3
328_18565 328_18973 796_4434 796_4605 796_14705
3.4
135_6557

ILLUMINA (ILLUMINA) : E107

Backcross Stats, 95% Limit

0.0 367_4617 367_4646 367_4835 547_1246
1.1 185_832
2.3 118_5116 556_413 556_481 556_635 787_4629
3.4 592_1107
9.1 305_2617 305_2829 462_684 492_1113 492_1711 492_1909
28.4 164_6023 415_1817
33.0 229_9053
35.2 229_9571 229_19453 229_19472 388_3371 388_3440 388_3513 388_3579 753_2962 753_9020 753_9095 863_5298 863_5299
40.9 84_6791 84_7519 506_1009
43.2 51_12268
44.3 51_14437 51_11050 51_11457 51_11471 51_11575 51_11608 51_11744 51_12294 51_12896 51_12921 51_12939 51_13024 51_13126 51_13202 5
45.5 51_36318
47.7 515_2484 515_2725 515_2777
50.0 74_8675 283_214 1028_13308 1028_13575 1028_14232 1028_14268 1028_14759 1028_35168 1028_39011 1028_39474 1028_39718
51.2 601_1394 601_1713
52.3 58_5420 114_5532 832_6756 832_6877
53.5 832_2289 832_2370 832_2424 832_2561 832_3105 832_3336 966_2410 966_5793 966_7766 966_7895 966_8016 966_8225
54.6 966_5049

ILLUMINA (ILLUMINA) : E108

Backcross Stats, 95% Limit

0.0	535_10920
2.3	535_88 535_5904 535_10110 535_10248 535_10409 535_10531 535_10776 535_10829 535_10897 535_11237 535_11398 535_11515 535_11[illegible]
5.7	408_10676
10.4	408_3340
13.9	408_2716 408_2766 408_3128 408_3136 408_3164 408_3189 408_3250 408_3298 408_3343 408_3540 408_3702 408_7035 408_7567 408_7[illegible]
15.1	
16.2	713_725 130_315 130_737 130_761 130_2321 130_2349 130_2352 130_2461 130_3281 130_4686 130_4921 130_5694 130_7071 52_486 52[illegible]
17.3	981_8874 96_3870 423_2019 460_181 460_1357 587_123 587_366 587_437 691_354 870_2341 870_4131 870_4313 870_6284
18.5	981_1609 96_1761 96_1738 396_289
32.1	577_695 577_708
33.3	115_416 115_460 115_614 115_803 115_4812 115_4815 115_5704 115_6124 115_7199 115_7415 115_7428 115_7916
34.4	115_8066
37.9	216_2485
40.1	906_4157 906_964 906_3023 906_3350 906_3731
41.3	906_6984
46.0	857_9326
47.1	316_3120
52.8	609_719 609_736 609_991
65.3	212_3995 242_1833
68.8	242_1739

ILLUMINA (ILLUMINA) : E109

Backcross Stats, 95% Limit

Position	Markers
0.0	546_2070
4.6	213_426 213_1777 213_4456 213_5187 213_6437 213_6460 379_2241
5.8	252_5249 704_7343 704_7448 704_7527
8.1	333_1712
9.2	333_3763 333_4551 333_4552 333_4572 333_4830 333_4848 333_4872 333_4892 333_5276
10.3	441_703
12.6	378_358 378_1015
14.9	792_8883 792_8909 792_8915
16.0	792_13459
18.3	811_3375 811_3400 811_3632
19.4	231_2985 303_3014 321_681 321_690 321_737 321_937 321_975 321_1019 390_2798 390_2845 390_2896 390_3110 390_3889 390_3961
20.6	390_3722
22.9	548_1848
24.1	548_1867 548_1988 548_1990 548_2183 548_2208 548_2541 548_2547 548_2668
25.2	1015_843 1015_2076 1015_2153 1015_2587 1015_4291 1015_5736 1015_5771 1015_5964 1015_5965 1015_6787 1015_6824 1015_8171 1015_8198
26.3	1015_2539
27.5	991_1599
28.6	991_1488 132_113 525_1050 616_1105 616_1112 651_1581
32.1	54_702 54_1389 54_1410 309_277 310_2510
33.2	312_1411 888_8053 888_8557 888_9954
34.4	456_2886 456_2984 456_3175 456_3759 1016_6801 1016_8554 1016_8836
36.6	169_11395
37.8	169_10461 581_540 751_817 751_1130 751_1146 751_2572 751_4446 751_4561 751_4633 751_4635 751_5093 751_5555 751_5586 751_8595 751_101[illegible]
38.9	169_6502
40.1	169_1533 169_1566 169_1689 169_1693 169_1752 169_1776 169_1799 169_2175 169_2197 169_3739 169_3748 169_3889 169_3931 169_4008 169_43[illegible]
41.2	169_11415 169_12379 169_16247 169_17210 169_17298
42.4	169_15088
44.6	497_3145 497_3183 497_3411 497_4081 497_5697 497_9779 497_11078 497_12274 497_13251 497_13260 497_15676 497_16067 497_16545 497_1659[illegible]
51.7	617_710

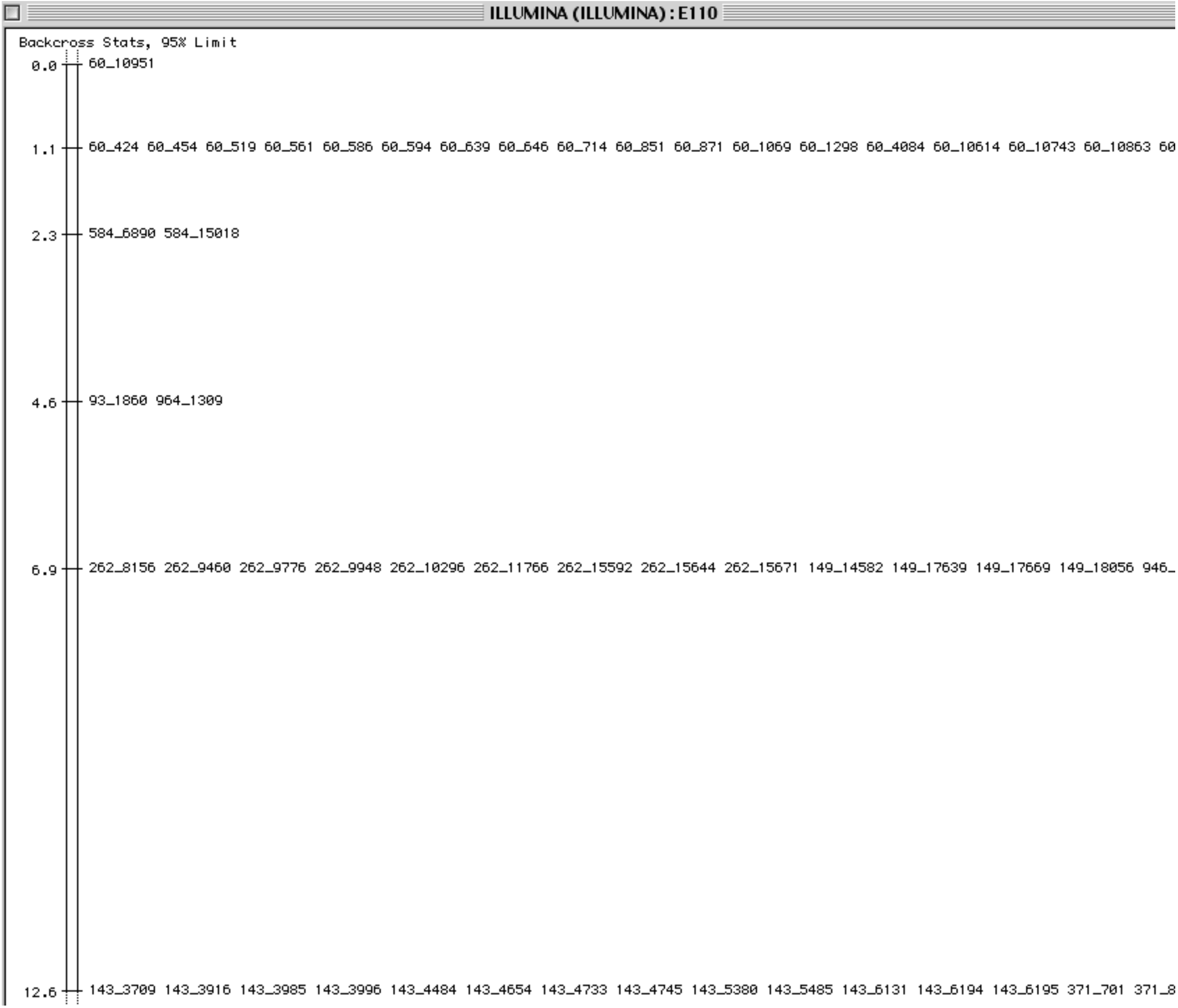

ILLUMINA (ILLUMINA) : E110
Backcross Stats, 95% Limit
0.0 60_10951
1.1 60_424 60_454 60_519 60_561 60_586 60_594 60_639 60_646 60_714 60_851 60_871 60_1069 60_1298 60_4084 60_10614 60_10743 60_10863 60
2.3 584_6890 584_15018
4.6 93_1860 964_1309
6.9 262_8156 262_9460 262_9776 262_9948 262_10296 262_11766 262_15592 262_15644 262_15671 149_14582 149_17639 149_17669 149_18056 946_
12.6 143_3709 143_3916 143_3985 143_3996 143_4484 143_4654 143_4733 143_4745 143_5380 143_5485 143_6131 143_6194 143_6195 371_701 371_8

ILLUMINA (ILLUMINA) : E111

Backcross Stats, 95% Limit

0.0 — 829_8910 829_9190

21.6 — 829_593 829_608 829_648 829_1137 829_1419 829_2017 829_2047 829_2431 829_2504 829_3016 829_3404 829_5040 829_5733 829_6434 829_6583 82[illegible]

34.2 — 782_722 782_974 782_1577 782_3712 782_3972 782_4725

53.8 — 85_3118 85_5296 296_636 296_903 296_1591 735_640 735_1494 735_1846 735_3299 735_4872 735_5529 735_5549 735_6161 735_6529 735_7451 735_[illegible]

ILLUMINA (ILLUMINA) : E112

Backcross Stats, 95% Limit

0.0 104_7898 104_7298 145_4758 145_7307 145_9698 145_10435 145_11637 145_11645 145_11706 145_11713 145_11764 145_11770 145_11954 145_119
1.1 277_3302 277_3352 277_3375 277_18636 277_22975 277_23281 277_24334 277_26216 277_26509 277_26675 965_4778 965_5767 965_5802
2.3 962_7227 962_7496 962_8223 962_8264 962_8355 962_10184 962_10714 962_10730 962_10911 962_11300 962_11367 962_11558 962_11586 962_117
3.4 919_57561
6.8 919_716 919_719 919_2157 919_2200 919_2221 919_6321 919_6525 919_6590 919_8862 919_20404 919_30450 919_30594 919_30647 919_30684 919
7.9 66_8189 66_8192 66_8235 66_8761
9.1 458_1992 815_8387 815_13052 815_13090 815_14304
13.7 797_12214 797_12272 797_12296
14.8 184_21182 1012_1605 1012_10984 1012_12112 1012_12202 1012_12386 1012_12845 1012_12907 1012_13123 1012_13352 1012_14104 1012_14767 10
21.7 823_10848
23.9 809_5859 809_5926 809_6376 809_7309 809_7320 809_9780 809_12122 809_13933 809_14083 809_16150 809_17505 809_18869 809_18989 809_1901
26.2 809_34554 809_41652 809_47633 809_47669 809_47700 809_47735 809_47739 809_47785 809_48931 809_50483 809_50515 809_52833 809_53706 80
28.6 809_90785 809_97218 809_97238 809_97669 809_97146
29.7 136_4047 402_167
30.9 618_695
33.2 383_984
34.4 383_2009
47.4 116_2250 237_4866 237_5563 237_6234 237_6292 237_6295 237_6411 477_963 674_787
48.5 237_5851
50.8 237_4778

ILLUMINA (ILLUMINA) : E113

Backcross Stats, 95% Limit

0.0	387_601 387_941 387_1113
1.1	570_1110 570_1265
11.4	614_703 614_755 614_768
12.5	550_682 788_1620 788_1765 788_5013
35.4	99_4695
36.6	99_27447
37.7	99_3023 99_24691 99_24725 99_27487 99_27612 191_7216 191_7923 191_12266 191_13078 191_13572 191_13696 191_14721 191_27601 191_27656 191_
38.8	740_2975 740_3149 740_3163 740_3233 740_3556 740_3605 740_4001 740_4992 740_6087 740_6290 740_6546 799_7519
40.0	799_1918 799_1970
41.2	799_7587 799_8307 799_10176 799_10311 799_10371 799_10410 799_10538 799_10585 799_11437 799_11446 799_14956
42.3	743_5690 743_5738 743_10771 743_11135 743_11237 717_316 967_3522
57.1	551_1463 693_268 866_2581 866_7847 866_7975 866_10377

ILLUMINA (ILLUMINA) : E114

Backcross Stats, 95% Limit

0.0	313_719 276_1983 161_7089 44_8065 266_293 266_330 266_397 266_565 266_835 266_2282 266_4219 266_4799 266_5316 266_5345 266_5450 266_5
6.8	245_5433 245_5956 270_957 471_2739 988_26826 988_26861 988_27331 988_27610 988_29020 988_29529 988_29545 988_29576 988_30499 988_3055
14.8	484_2194
19.4	139_1582 163_1827 163_2964 163_3241 163_3349 163_3765
26.2	899_1462
31.9	921_1098 921_1435 921_1747 921_1784 921_2495 921_2499
34.2	699_20127 699_20140
35.3	871_1776 871_1781 871_3566 871_3638
37.6	658_1435
39.9	111_2826 256_2898 256_2929 659_607 704_2488 704_3106 404_173 404_199 404_367 404_370 404_467 404_490 404_594
41.1	404_210
44.5	318_949
46.8	405_2840

ILLUMINA (ILLUMINA) : E115

Backcross Stats, 95% Limit

0.0	107_11943
1.1	107_1811 107_8808 107_9321 107_9570 107_10735 107_11061 107_11951 107_19242 107_19266 107_19286 107_19475 107_25759 107_27475 790_12[illegible]
3.4	49_111 933_7483 933_7509
4.5	95_1721
5.7	278_1970 428_2501 765_369 765_408
6.8	765_971 765_1134 765_1626 765_1703 765_2844 765_7288 765_11051 765_11071 765_11080 765_12097 765_12196
9.1	255_5517 255_5629 255_5714 255_5726 255_5777 849_5999 849_6064 849_6264 849_7115 849_7450 849_9002 849_10310 849_10338 849_10622 849_[illegible]
10.2	849_973 849_1087 849_1125 849_1155 849_5742 849_5924 849_5934 849_6009 1026_4289 1026_4502 1026_4657 1026_4721 1026_4783 1026_4884 1[illegible]
11.4	739_573 739_383 739_576 739_735 739_755 739_5478 739_5671 739_5691 739_5712 739_5739 739_5989 739_6043 739_6304 739_6316 739_6425
12.5	308_2073 308_4516 308_4596 308_4801 308_4909 308_5103 322_1675 322_3860
13.7	156_3473 442_2908 442_4579 442_4590 442_4804 442_4940 442_4997 442_5147
14.8	141_312 141_354 141_456 141_655 141_1672 141_1739
16.0	320_3904 320_3919 320_4110 341_2772 341_2999
19.4	330_2832 344_2691
25.1	590_771 590_812
35.5	918_1753 918_2407 983_7892
44.5	619_9084 619_10448
49.3	710_608
57.6	496_6855 496_9544 496_9672 496_9696 496_9924 496_11052 496_14064 496_15995 496_18304
62.2	680_1650
66.7	377_635
69.0	411_3520 416_1339 650_1339 989_9440
70.1	90_1980

ILLUMINA (ILLUMINA) : E116

Backcross Stats, 95% Limit

0.0 380_80 380_663 380_695 380_783 380_1457 380_1774 380_2468 380_2545 380_2613 380_2779 380_3689 380_3713 380_3735 380_3858 [illegible]

1.1 855_680 855_732 855_1109 855_1145 855_1171 855_1224 855_1927 855_2226 855_2454 855_2912 241_353 241_628 241_733 241_1053 [illegible]

2.3 113_4206

ILLUMINA (ILLUMINA) : E117

Backcross Stats, 95% Limit

0.0 122_3038 122_3074

1.1 400_3263 426_3181 426_3229 426_3349 748_9032 748_9033 748_10510 748_11150 785_4193 785_4436 785_4458 785_4563 785_4632 785_4752 785

2.3 816_2366

4.6 1018_10110 1018_19036 1018_19554 1018_20179 1018_20673 1018_21098 1018_21141 1018_23772 1018_25409 1018_33337 1018_36378 779_235 77

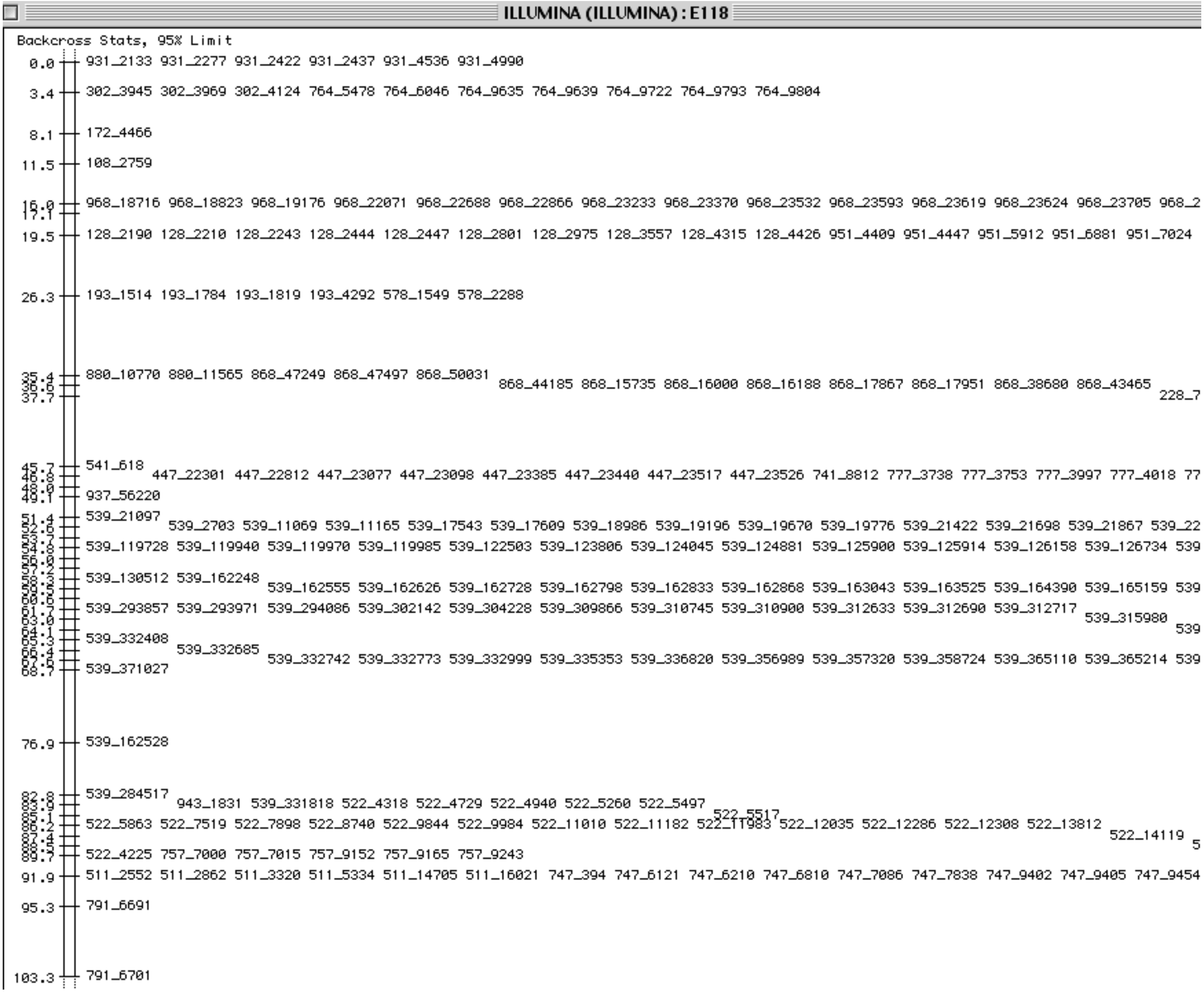
ILLUMINA (ILLUMINA) : E118
Backcross Stats, 95% Limit
0.0 931_2133 931_2277 931_2422 931_2437 931_4536 931_4990
3.4 302_3945 302_3969 302_4124 764_5478 764_6046 764_9635 764_9639 764_9722 764_9793 764_9804
8.1 172_4466
11.5 108_2759
16.0
17.1 968_18716 968_18823 968_19176 968_22071 968_22688 968_22866 968_23233 968_23370 968_23532 968_23593 968_23619 968_23624 968_23705 968_2
19.5 128_2190 128_2210 128_2243 128_2444 128_2447 128_2801 128_2975 128_3557 128_4315 128_4426 951_4409 951_4447 951_5912 951_6881 951_7024
26.3 193_1514 193_1784 193_1819 193_4292 578_1549 578_2288
35.4 880_10770 880_11565 868_47249 868_47497 868_50031
36.6 868_44185 868_15735 868_16000 868_16188 868_17867 868_17951 868_38680 868_43465
37.7 228_7
45.7 541_618
46.8 447_22301 447_22812 447_23077 447_23098 447_23385 447_23440 447_23517 447_23526 741_8812 777_3738 777_3753 777_3997 777_4018 77
48.0
49.1 937_56220
51.4 539_21097
52.6 539_2703 539_11069 539_11165 539_17543 539_17609 539_18986 539_19196 539_19670 539_19776 539_21422 539_21698 539_21867 539_22
53.7
54.8 539_119728 539_119940 539_119970 539_119985 539_122503 539_123806 539_124045 539_124881 539_125900 539_125914 539_126158 539_126734 539
56.0
57.2
58.3 539_130512 539_162248
59.5 539_162555 539_162626 539_162728 539_162798 539_162833 539_162868 539_163043 539_163525 539_164390 539_165159 539
60.6
61.7 539_293857 539_293971 539_294086 539_302142 539_304228 539_309866 539_310745 539_310900 539_312633 539_312690 539_312717
63.0 539_315980
539
64.1
65.3 539_332408
66.4 539_332685
67.6 539_332742 539_332773 539_332999 539_335353 539_336820 539_356989 539_357320 539_358724 539_365110 539_365214 539
68.7 539_371027
76.9 539_162528
82.8 539_284517
83.9 943_1831 539_331818 522_4318 522_4729 522_4940 522_5260 522_5497
522_5517
85.1
86.2 522_5863 522_7519 522_7898 522_8740 522_9844 522_9984 522_11010 522_11182 522_11983 522_12035 522_12286 522_12308 522_13812
522_14119
5
87.4
88.5
89.7 522_4225 757_7000 757_7015 757_9152 757_9165 757_9243
91.9 511_2552 511_2862 511_3320 511_5334 511_14705 511_16021 747_394 747_6121 747_6210 747_6810 747_7086 747_7838 747_9402 747_9405 747_9454
95.3 791_6691
103.3 791_6701

ILLUMINA (ILLUMINA) : E119

Backcross Stats, 95% Limit

0.0 140_5183 140_5605 226_402 226_3136 226_3175 479_17312 479_17317 479_17503 479_17538 719_5065 746_297 746_703 746_1136 746_2932 746_[illegible]

3.4 162_2631 162_4381 162_5816 162_5955 162_5980 162_6016 162_6077 162_6098 162_6360 977_9650

4.5 392_3210

9.1 1011_5108

13.9 606_1064 606_1186 606_1420 606_1487 606_1517 606_1595

15.1 606_1593

23.1 692_2172 692_2196 692_3121 692_3179 692_4371 692_4407 692_4492 692_4716 692_5046 692_5082 692_6724 692_8663 692_13768 692_14167 692_[illegible]

24.3 692_14269

27.9 700_825

29.1 700_1085 1031_7889

37.1 752_6514 752_6633 752_6634 752_6677 752_7741 752_8677 752_8859 752_8910

39.3 207_1006 207_1009 207_1102 207_3796 211_4183 211_5420 567_2112

48.4 591_1882

49.6 274_5069

51.9 836_3445

54.1 593_2024 675_544 675_552 675_576 675_580 675_1100

55.3 475_1929 475_1991 915_7787 915_4855 915_5195 915_5215

61.0 915_7105

ILLUMINA (ILLUMINA) : E120

Backcross Stats, 95% Limit

0.0 — 337_5299 459_1087
1.1 — 459_378 459_744 459_781 459_812
2.3 — 953_295 953_378 953_493
3.4 — 337_3724 337_3726 297_5183 889_1407 189_2043 889_4122 889_4148 889_4491 889_4516 889_6964 889_7017 889_7182 889_7618 889_7692 [illegible]
4.6 — 189_5895 189_6107
9.1 — 386_3807
11.4 — 382_2809
13.7 — 560_618
14.9 — 395_584 395_1876 395_1879 395_2480 395_2503 395_2558 395_2608 395_2791 395_3115 395_3253 395_3329 418_1810
18.3 — 560_577
20.6 — 560_627
26.3 — 251_6001 251_6002 595_1675 687_626 932_6530 932_6756
28.6 — 841_11517 841_725 841_794 841_1605 841_5336 841_6290 841_6296 841_7343 841_7806 841_8090 841_8487 841_8496 841_9231 841_9356 [illegible]
30.9 — 841_10167
32.1 — 841_10182
33.3 — 845_2439 845_2537 845_2543 845_2567 845_2577 845_3515 845_3607 845_3755 845_3916 845_3959 845_3985 845_3988 845_4040 845_4106 [illegible]
34.4 — 845_16783 845_17477 845_18502 845_23795 845_24400 845_28450 845_28477 845_28623 845_30123 845_30129 845_54416 845_55789
35.6 — 845_70429 [illegible]
36.7 — 365_4472 454_2460 892_3982 892_9961 892_10262 892_10372 892_10499
50.3 — 516_2050
54.9 — 258_2160
56.0 — 258_2136 258_2164
58.3 — 667_768 822_12903 822_13063
59.5 — 775_1217 775_1719 775_1953 775_1984 775_2562 775_4769
61.8 — 167_2880 167_2910 167_3002 167_3361 167_3396 425_1018 425_1021 425_1044
62.9 — 708_831
64.1 — 514_2420 948_426 948_1620
70.9 — 47_8509 627_1186 627_1380 627_1534 627_2139

ILLUMINA (ILLUMINA) : E121

Backcross Stats, 95% Limit

0.0 — 407_1489

1.1 — 407_498 407_610 407_845 407_880 407_1014 407_1157 407_1189 407_1221 407_1241 407_1462 407_1491 182_423 182_436 182_1120 182_1151 182[illegible]

2.3 — 67_7663 203_4098 203_4151 203_4234 203_4283 360_309 360_3946 361_3103 734_6826 734_6834 847_12396 438_623 438_846 438_868 543_626 54[illegible]

3.4 — 644_842

4.5 — 644_852 644_1009 644_1046 644_1089 644_1090 644_1227 644_1310

5.7 — 644_1365

6.9 — 644_1374 644_1417 644_1447 644_1450 644_1480 644_1522

8.0 — 644_1573

9.2 — 644_1747 644_1757 925_1618 925_1664 1005_510 1024_8016 1030_8494 1030_9213

10.3 — 1030_2041

ILLUMINA (ILLUMINA) : E122

Backcross Stats, 95% Limit

0.0 — 39_1430 196_1334 196_1598 346_134 346_154 346_165 357_2613 444_295 598_703 603_405 694_757 755_2029 755_2329 755_2437 755_3559 [illegible]

1.1 — 860_10686 600_1415 860_23497

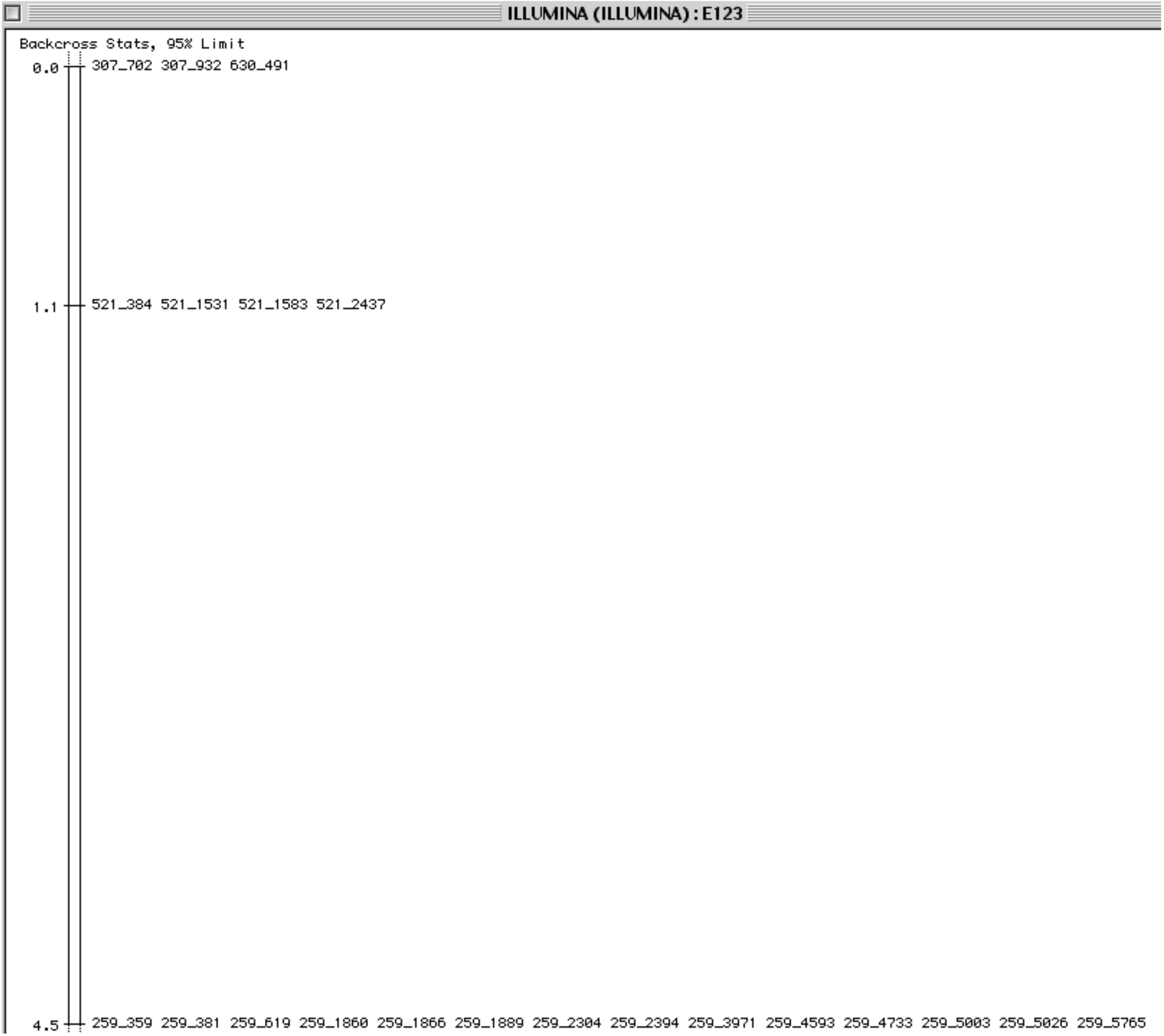
ILLUMINA (ILLUMINA) : E123
Backcross Stats, 95% Limit
0.0
307_702 307_932 630_491
1.1
521_384 521_1531 521_1583 521_2437
4.5
259_359 259_381 259_619 259_1860 259_1866 259_1889 259_2304 259_2394 259_3971 259_4593 259_4733 259_5003 259_5026 259_5765

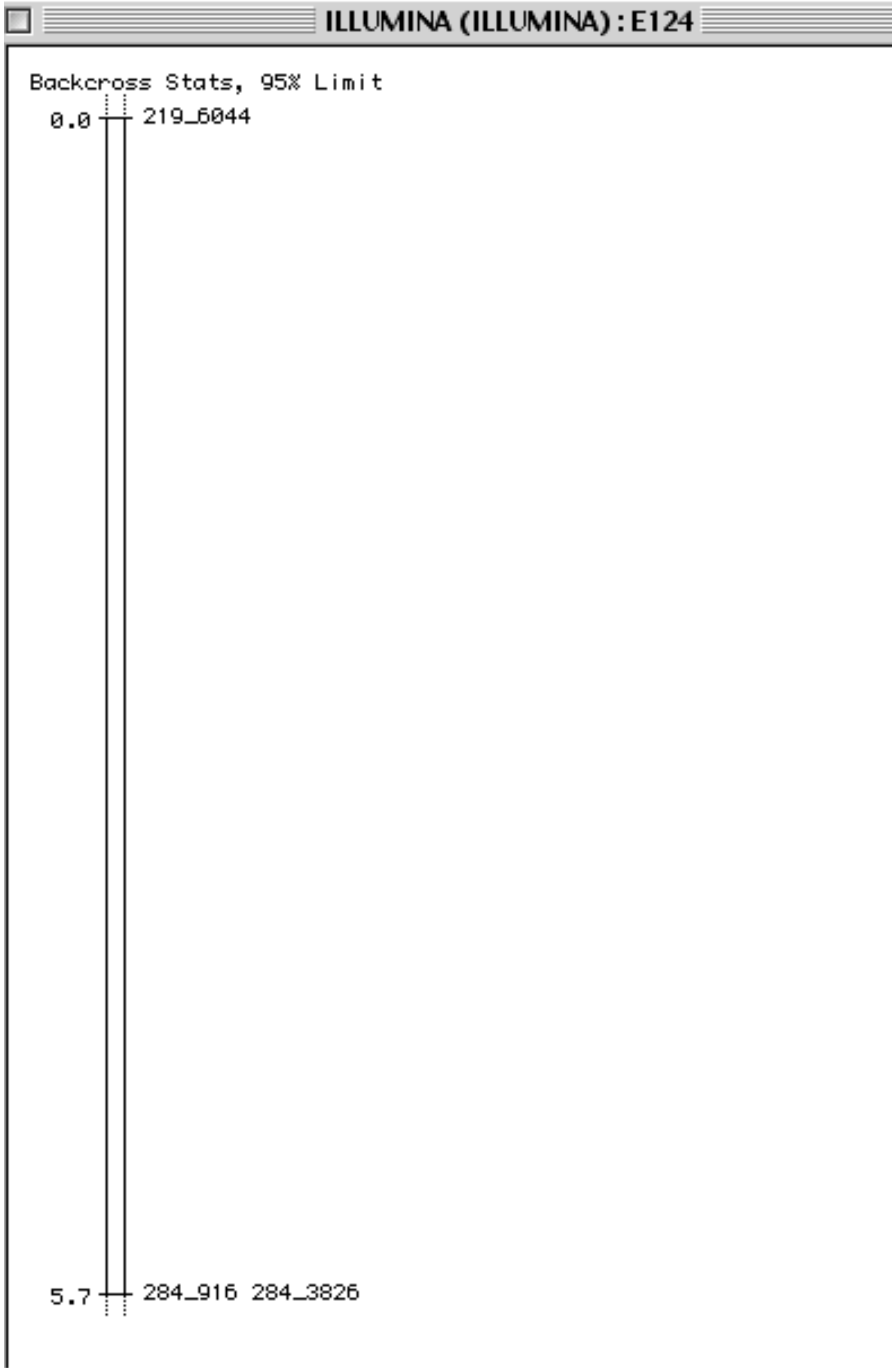
ILLUMINA (ILLUMINA) : E124
Backcross Stats, 95% Limit
0.0
219_6044
5.7
284_916 284_3826

ILLUMINA (ILLUMINA) : E125

Backcross Stats, 95% Limit

0.0 293_410 293_449 293_868 293_2151 293_2332 293_3925 293_4354 293_4369 293_4723 293_4788 293_5341 293_5411 293_5651

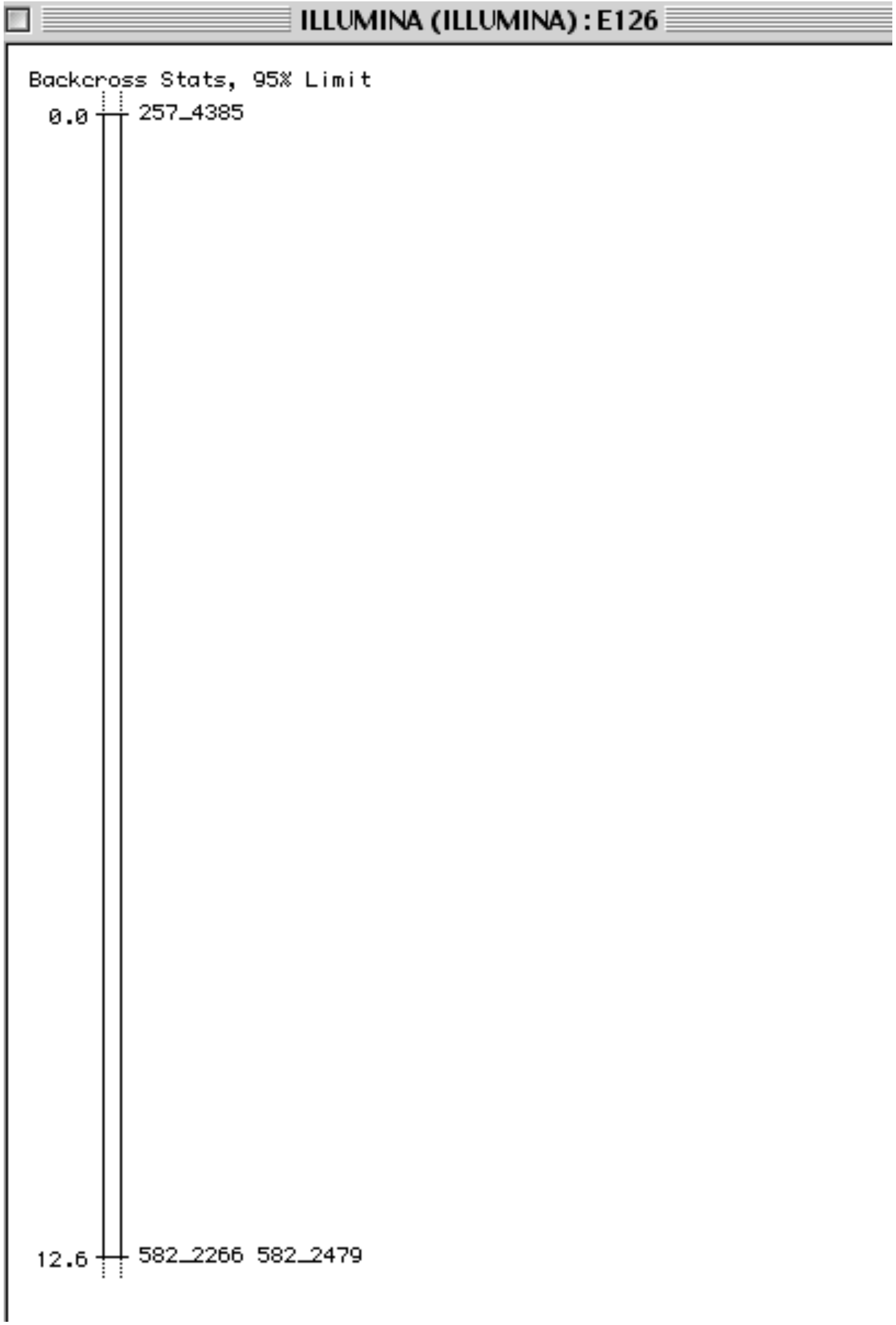
ILLUMINA (ILLUMINA) : E126
Backcross Stats, 95% Limit
0.0
257_4385
12.6
582_2266 582_2479

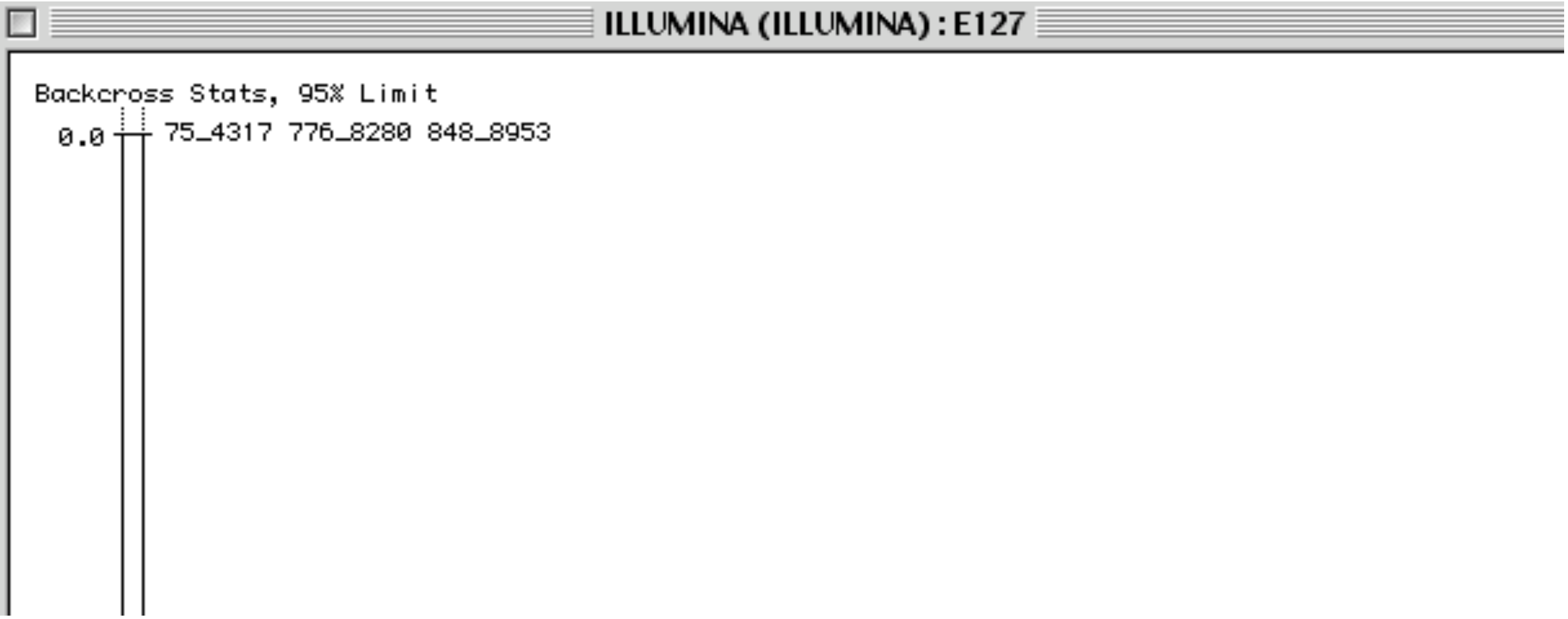
ILLUMINA (ILLUMINA) : E127
Backcross Stats, 95% Limit
0.0
75_4317 776_8280 848_8953

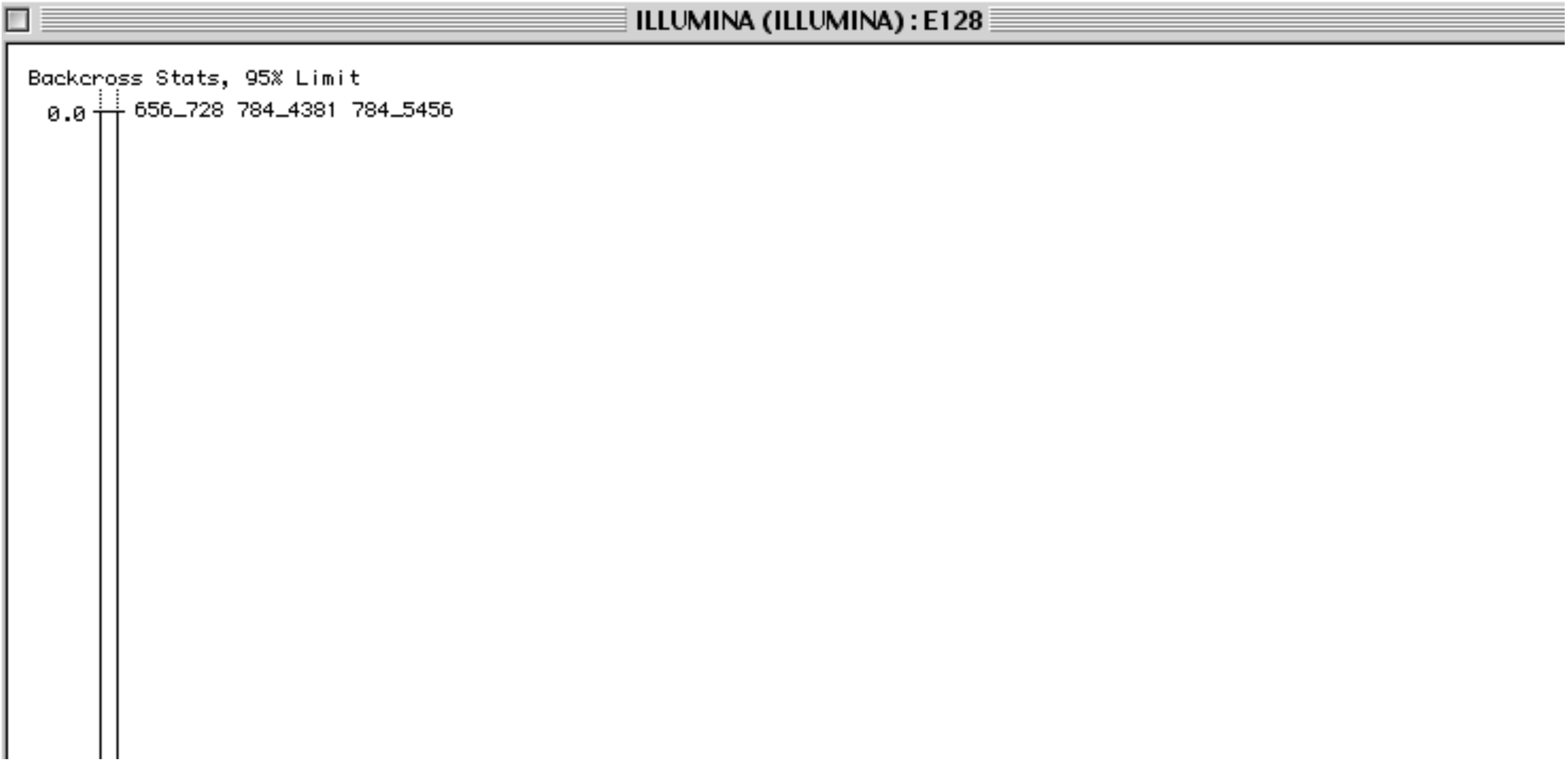
ILLUMINA (ILLUMINA) : E128
Backcross Stats, 95% Limit
0.0
656_728 784_4381 784_5456

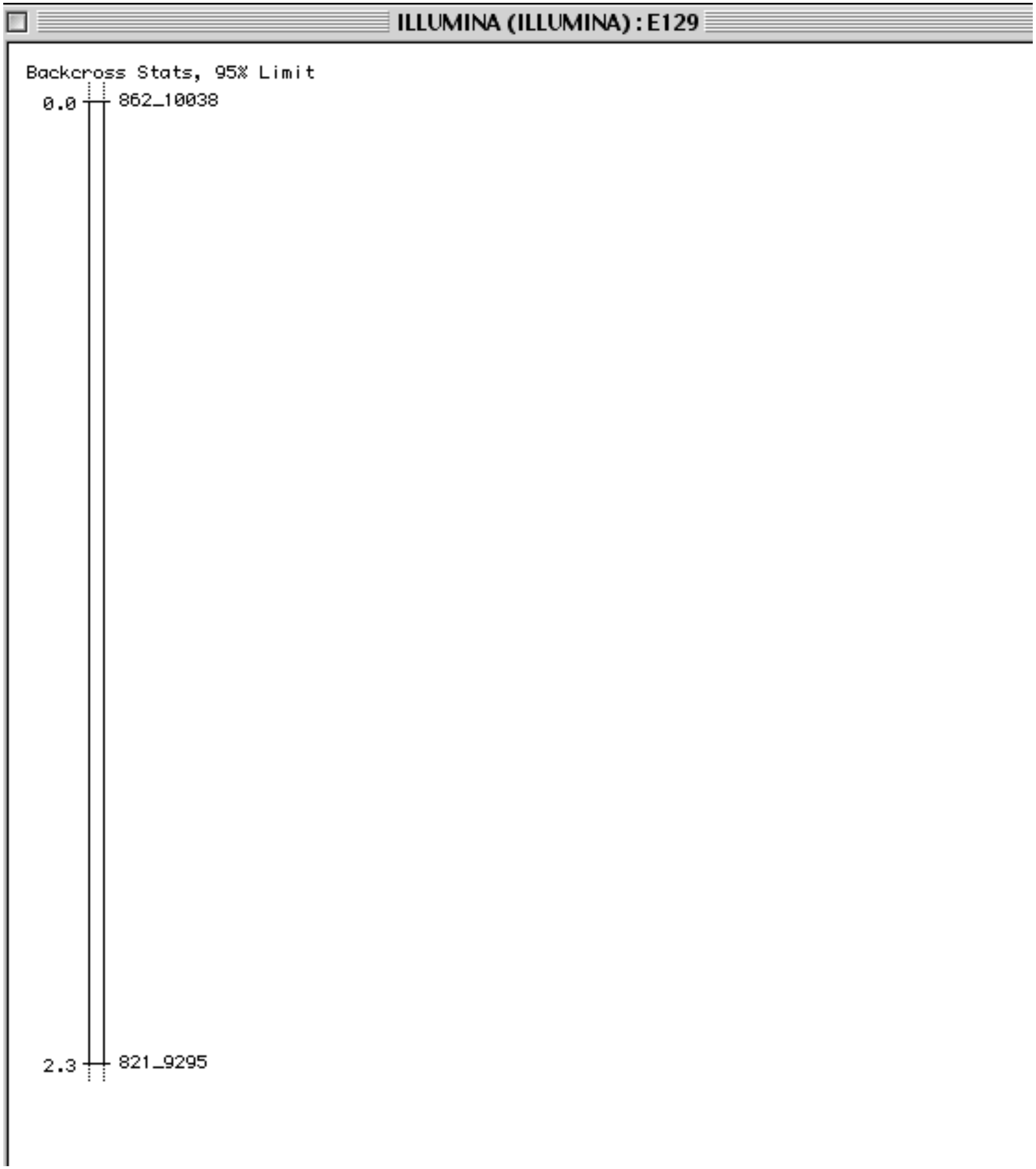
ILLUMINA (ILLUMINA) : E129
Backcross Stats, 95% Limit
0.0
862_10038
2.3
821_9295
