# Supplementary material for: A New Chicken Genome Assembly Provides Insight into Avian Genome Structure
Source: G3 (Bethesda). 2016 Nov 14;7(1):109–17. doi: 10.1534/g3.116.035923 (PMC5217101; doi:10.1534/g3.116.035923)
Supplement: Supplementary file 21 [file 109FileS10.docx]

File S10. A summary of newly developed SNP markers with Affymetrix SNP indentification, assigned linkage group, associated flanking sequence of each SNP and position with each Gallus_gallus-5.0 unplaced assembly scaffold. (.xlsx, 303 KB)

<http://www.g3journal.org/lookup/suppl/doi:10.1534/g3.116.035923/-/DC1/FileS10.xls>
