# Supplementary material for: A New Chicken Genome Assembly Provides Insight into Avian Genome Structure
Source: G3 (Bethesda). 2016 Nov 14;7(1):109–17. doi: 10.1534/g3.116.035923 (PMC5217101; doi:10.1534/g3.116.035923)
Supplement: Supplementary file 23 [file 109TableS1.docx]

**Table S1.** Assembly contiguity metrics of iterative builds of the Gallus_gallus-5.0 reference

| Metrics | First Draft^1^ | Second Draft^2^ | Gallus_gallus-5.0^3^ |
| --- | --- | --- | --- |
| Total base length (bp) | 1,214,726,541 | 1,217,777,931 | 1,230,258,557 |
| Total contigs | 28,516 | 26,784 | 24,693 |
| N50 contig length (bp) | 1,071,268 | 2,382,199 | 2,894,815 |
| Total scaffolds | NA | 25,309 | 23,870 |
| N50 scaffold length (bp) | NA | 5,580,786 | 6,379,610 |

NA – not applicable

^1^ De novo assembly of all error corrected PacBio reads only produces contigs.

^2^ Statistics after SSPACE scaffolding of the first draft, PBJelly gap filling, icorn2 correction

^3^ Final metrics after merge with the Gallus_gallus-4.0 assembly.
