# Supplementary material for: A New Chicken Genome Assembly Provides Insight into Avian Genome Structure
Source: G3 (Bethesda). 2016 Nov 14;7(1):109–17. doi: 10.1534/g3.116.035923 (PMC5217101; doi:10.1534/g3.116.035923)
Supplement: Supplementary file 24 [file 109TableS2.docx]

**Table S2**. Classification of chicken protein coding genes in Gallus_gallus-5.0. The transcripts and genes in annotation release 103, Gallus_gallus-5.0, may have a new identifier but may map very well to genes and transcripts in annotation release 102, Gallus_gallus-4.0, hence are not in Current-novel, Current-other, or Current-unmapped category. We classify changes in genes and transcript based on exonic overlap between the two gene annotation releases (with some adjustment for exon boundaries).

|  | Current-novel | Current-unmapped | Current-other | All |
| --- | --- | --- | --- | --- |
| No of new protein coding genes | 980 | 2129 | 350 | 3459 |
| No of corresponding transcripts | 1436 | 2675 | 521 | 4632 |
| No of corresponding proteins | 1357 | 2561 | 478 | 4396 |
| Proteins with at least one conserved profile | 1016 (74.9%) | 1902 (74.3%) | 386 (80.8%) | 3304 (75.2%) |
| Unique Pfam domains | 361 | 793 | 189 | 1075 |
| Unique SUPERFAMILY | 178 | 345 | 108 | 421 |
| Unique Gene3D models | 173 | 337 | 106 | 419 |
| Unique ProSiteProfiles | 128 | 260 | 79 | 309 |
| Unique SMART domains | 132 | 224 | 73 | 282 |
| Unique PRINTS fingerprints | 69 | 179 | 38 | 227 |
| Unique ProSitePatterns | 57 | 143 | 46 | 178 |
| Unique TIGRFAM families | 12 | 31 | 3 | 42 |
| Unique PIRSF families | 12 | 27 | 6 | 42 |
| Unique ProDom domains | 10 | 16 | 3 | 27 |
| Unique Hamap profiles | 5 | 7 | 2 | 14 |
| **Unique Interpro profiles** | 611 | 1297 | 360 | 1705 |
| Proteins with Interpro GO terms | 657 (48.4%) | 1226 (47.9%) | 287 (60.0%) | 2170 (49.4%) |
| Unique GO terms assigned by interpro | 238 | 504 | 148 | 615 |
| Proteins with Blast2GO GO terms | 773 (57.0%) | 1562 (61.0%) | 332 (69.5%) | 2665 (60.0%) |
| Proteins with Blast2GO GO terms | 747 | 1853 | 447 | 2275 |
