# Supplementary material for: A New Chicken Genome Assembly Provides Insight into Avian Genome Structure
Source: G3 (Bethesda). 2016 Nov 14;7(1):109–17. doi: 10.1534/g3.116.035923 (PMC5217101; doi:10.1534/g3.116.035923)
Supplement: Supplementary file 25 [file 109TableS3.docx]

**Table S3**. Mammalian or lizard genes that are not present in other sequenced birds with annotated gene sets. See methods for a description of search details.

| **Gene** | **Annotation classification** |
| --- | --- |
| ABCD1 | none |
| ADCY4 | None |
| ADGRE5 | None |
| AIF1 | None |
| ANKRD23 | None |
| AP1G2 | Chicken locus chrUn_AADN03013594:1098-1347 is misannotated; this is AP1M2 |
| AP2A1 | None |
| APEX2 | None |
| APLP1 | None |
| APOM | None |
| ARAF | None |
| ARHGEF25 | Prediction 104563476 in speckled mousebird and 104034032 in pellican are misannotated; they are TRIO |
| ASF1B | None |
| ATL3 | None |
| ATP6AP1 | Prediction 427854 is a previously described ATP6AP1-like paralog (Lovell et al., 2015; Table S2) |
| AVPR2 | Prediction 427860 is a previously described AVPR2-like paralog (Lovell et al., 2015; Table S2) |
| BCL7C | None |
| BGN | None |
| BRSK1 | None |
| C14orf119 | None |
| C14orf93 | None |
| C19orf81 | None |
| C2CD4D | None |
| CA11 | None |
| CACNG6 | None |
| CCDC114 | None |
| CCDC151 | none |
| CCDC159 | None |
| CCDC64B | None |
| CCDC88B | None |
| CCHCR1 | None |
| CCNB1IP1 | None |
| CCT8L2 | No evidence of HUGO or Gene Description in birds; lizard model blast to nucleotide does not hit to birds. |
| CDC42EP5 | None |
| CDH24 | None |
| CDKN2D | None |
| CEBPE | None |
| CHMP4A | None |
| CLEC11A | None |
| CNNM3 | None |
| CNTD2 | None |
| CNTROB | None |
| CORO1A | None |
| CPT1C | None |
| CRB3 | Predictions 108639966 in manakin and 104559394 in mousebird (and in other birds) are misannotated; this is CRB2 |
| CYB5D1 | None |
| CYP2F1 | None |
| DENND1C | Prediction 104559395 in mousebird is misannotated; this is DENND1B |
| DNAH2 | None |
| DNAJC4 | None |
| DOC2A | None |
| DOCK6 | None |
| DPP3 | none |
| DUSP9 | None |
| EFS | none |
| EGLN2 | None |
| ELK1 | None |
| EPN1 | none |
| EPS8L1 | none |
| ESRRA | None |
| FAM120C | None |
| FAM71E1 | None |
| FAM98C | None |
| FBRS | None |
| FBXL19 | None |
| FERMT3 | None |
| FFAR1 | no evidence of HUGO or Gene Description in birds; lizard model blast to nucleotide does not hit to FFAR1 in birds. |
| FGD1 | None |
| FGF21 | None |
| FOXA3 | None |
| GAPDHS | None |
| GDI1 | None |
| GLS2 | Sandgrouse prediction 104460934 is GLS based on synteny and BLAT alignments in lizard. |
| GMPR2 | None |
| GNG8 | Prediction 106487999 from kiwi is misannotated, this is GNG2 or 4 based on BLAT and BLAST; no synteny information |
| GPR173 | None |
| GPR84 | None |
| GRAMD1A | None |
| GSK3A | None |
| HAS1 | None |
| HAUS7 | None |
| HIGD1C | None |
| HOMEZ | None |
| HSD17B14 | None |
| HSPB6 | None |
| IGLON5 | None |
| IL27RA | None |
| IRAK1 | None |
| IRF3 | None |
| IRF9 | Prediction 395243 is likely IRF10, or related paralog of IRF9 (e.g. LOC102561447 in Alligator) |
| ITPKC | None |
| IZUMO2 | None |
| JPH4 | None |
| KANK2 | None |
| KCNAB3 | None |
| KCND1 | None |
| KCNJ14 | Prediction 428244 is likely a KCNJ14-Like paralog also present in Alligator (LOC102575715) |
| KCNK4 | Prediction 423536 is likely a KCNK6-Like paralog as in other sauropsids, or possibly KCNK16. |
| KCNN4 | None |
| KDELR1 | None |
| KIFC2 | None |
| KIRREL2 | None |
| KLC2 | None |
| KMT2B | None |
| KREMEN2 | None |
| LAGE3 | None |
| LENG9 | None |
| LRP10 | None |
| LRRC8E | None |
| MACROD1 | None |
| MAGED2 | Previously reported Chicken locus in chrUn_JH376078:8302-11267 is MARS, not MAGED2 |
| MAMSTR | None |
| MAPK3 | None |
| MARCH9 | None |
| MDP1 | None |
| MFSD3 | None |
| MIA | None |
| MMP25 | None |
| MYADM | Predictions in other birds (e.g. 104913348) are actually MYADML |
| MYH14 | None |
| MYL6B | None |
| MYPOP | Prediction 107050911 is incorrectly annotated and BLAT aligns to SF1 in human and alligator. |
| NFATC4 | None |
| NR1H2 | None |
| NTF4 | None |
| NTN5 | None |
| NUCB1 | None |
| NUDT22 | None |
| ORAI3 | None |
| PABPN1 | Previously reported chicken locus in chrUn_AADN03018734:270-282 is PABPN1-Like, not PABPN1 |
| PACS1 | Prediction 421254 (and others in birds) is a previously described PACS1-like paralog (Lovell et al., 2015 Tables S2 and S3) |
| PAFAH1B3 | None |
| PALM3 | None |
| PAQR4 | None |
| PARP2 | Prediction 104541240 in brown roatelo is most likely PARP3 |
| PELI3 | None |
| PHKG2 | None |
| PIM2 | None |
| PLEKHA4 | Prediction 104911032 in turkey is incorrectly annotated, this is PLEKHA7 |
| PLXNA3 | None |
| PLXNB3 | None |
| PNCK | None |
| PODNL1 | None |
| POU5F1 | None |
| PPFIA3 | None |
| PPP1R3E | Predictions 395787 and 107054319 are incorrectly annotated. 107054319 is a novel paralog of PPP1R3E, and 395787 is a novel paralog of PPP1R3C. Both are in lizard at their respective syntenic positions, neither is PPP1R3E. |
| PPP1R3F | None |
| PPP6R1 | None |
| PRDX2 | None |
| PRDX5 | None |
| PRKACA | Chicken prediction 100859181 is either a novel paralog of PRKACA, or a gene with conserved synteny in alligator that is unrelated to PRKACA |
| PROSER3 | None |
| PRR13 | Chicken model ENSGALG00000023715 is discontinued and is not PRR13; other bird predictions are also not PRR13 but might be FTCD based on alignments to alligator. |
| PRR14 | None |
| PRRG2 | None |
| PRSS53 | None |
| PSMB11 | None |
| PSME1 | None |
| PSME2 | None |
| PTGIR | None |
| PTPRH | None |
| RAB1B | None |
| RAB3D | None |
| RASGRP4 | None |
| RBCK1 | Prediction 108491987 in manakin is misannotated; it is most likely SHARPIN |
| RCN3 | None |
| REM2 | None |
| RETN | None |
| RGL3 | None |
| RIPK3 | Predictions 415708, 104687489, and 103620177 are a previously reported RIPK3-like paralog (Lovell et al., 2015; Table S2 and S3). |
| RLN3 | Prediction 104695129 in crow is misannotated; it is flanked by WDR78 and TCTEXD1; in human, these genes flank INSL5; crow prediction is INSL5 as supported by alignments in alligator. |
| RNF181 | Prediction 104374778 in turaco is misannotated; this is RNF126 |
| RPGRIP1 | None |
| RPS6KA4 | None |
| RRAS | None |
| SAC3D1 | None |
| SAPCD1 | None |
| SERPINI2 | None |
| SLC10A3 | None |
| SLC22A17 | None |
| SLC25A23 | None |
| SLC26A10 | None |
| SLC44A4 | None |
| SLC6A16 | None |
| SLC6A8 | Prediction 100861584 (and many other predictions in birds) is a previously described SLC6A8-like paralog (see Lovell et al., 2015 Table S2, S3) |
| SLC7A7 | Prediction 420216 is a previously described SLC7A7-like paralog (see Lovell et al., 2015 Table S2,S3) |
| SLC7A8 | None |
| SNX15 | None |
| SPHK2 | None |
| SPIB | None |
| SPRED3 | None |
| SPRYD4 | Prediction 104634119 in crane is incorrectly annotated; this is CMYA5 |
| STX10 | None |
| STXBP2 | None |
| SYN1 | None |
| SYNGR4 | None |
| SYP | Prediction 395815 is PTPN11, which is also known as SYP. However, this is not the ortholog of human SYP |
| SYT5 | None |
| TBC1D10B | None |
| TBX6 | Prediction 395792 is a previously reported TBX6-Like paralog (see Lovell et al., 2015 Table S2, S3) |
| TCF19 | None |
| TEAD2 | None |
| TEP1 | None |
| THEM5 | Turkey prediction ENSMGAG00000007160 and chicken prediction ENSGALG00000026039 are both misannotated and are THEM4. |
| TIMP1 | Prediction 399507 appears to be a record error |
| TM9SF1 | None |
| TMC4 | None |
| TMEM143 | None |
| TMEM150A | Prediction 423759 is previously reported TMEM150A-like paralog (see Lovell et al., 2015 Tables S2 and S3). |
| TMEM150B | None |
| TMEM187 | None |
| TMEM205 | Prediction 104354799 in Cuckoo (and other birds) is flanked by ACOT11 and TTC4 and misannotated as TMEM205; it is a new paralog or an unrelated gene |
| TMEM86B | None |
| TMEM88 | None |
| TMEM91 | None |
| TNFAIP8L2 | None |
| TRIB3 | Prediction 428634 is the ortholog of a kinase related to a likely ortholog in gator LOC102573442 |
| TRPM4 | None |
| TSKS | None |
| TSPAN16 | None |
| TTC9B | None |
| TTYH1 | None |
| TUBB4A | None |
| TULP2 | None |
| USP11 | None |
| VSIG10L | Prediction 104490723 in cuckoo roller seems misannotated, it does not BLAST align to this gene in any other species; may be SLAM family member |
| VWA7 | Prediction 104344543 is misannotated; it is HCMN1 based on BLAST alignments |
| WDR87 | None |
| YIF1A | Prediction 107307713 in quail is misanotated; it is YIF1B |
| YPEL3 | None |
| ZNF784 | None |
| ZSWIM4 | None |
