# Supplementary material for: A New Chicken Genome Assembly Provides Insight into Avian Genome Structure
Source: G3 (Bethesda). 2016 Nov 14;7(1):109–17. doi: 10.1534/g3.116.035923 (PMC5217101; doi:10.1534/g3.116.035923)
Supplement: Supplementary file 26 [file 109TableS4.docx]

**Table S4**. Mammalian or lizard genes that are found in sequenced birds but not Gallus_gallus-5.0. See methods for a description of search details.

| **Gene** | **Other avian gene prediction** |
| --- | --- |
| ABCF1 | Tit prediction does not BLAST align to chicken |
| ADCK5 | Tit prediction 102113534 does not BLAST align to chicken |
| ALDH16A1 | Tit prediction does not BLAST align to chicken |
| ALKBH7 | Tit prediction 102113600 and sequence from Hron et al., 2015 do no BLAST align to chicken. |
| ARHGAP9 | Goose prediction does not BLAST align to chicken. |
| ATF6B | Tit prediction does not BLAST align to chicken |
| ATG9B | Tit prediction does not BLAST align to chicken |
| ATP2B3 | Starling prediction with correct synteny falsely BLAST aligns to ATP2B2 in chicken |
| B4GALNT1 | Tit prediction 102104849 does not BLAST align to chicken |
| B9D2 | Tit prediction 102112591 does not BLAST align to chicken |
| BAG6 | Tit prediction does not BLAST align to chicken |
| BAX | Tit prediction does not BLAST align to chicken |
| BCAT2 | Prediction 107199047 in great tit and 102104743 in Tibetan tit do not BLAST align to chicken |
| BEST2 | Tit prediction does not BLAST align to chicken. 101751224 is correctly annotated, but is not in the current release |
| BOLA2B | Tit prediction does not BLAST align to chicken |
| BSCL2 | Tit prediction does not BLAST align to chicken |
| C18orf54 | Goose prediction does not BLAST align to chicken. |
| C19ORF54 | Tit prediction 102112213 does not BLAST align to chicken |
| C2orf68 | Tit prediction does not BLAST align to chicken |
| CALM3 | Prediction 105414414 in eagle does not BLAST align to chicken |
| CARM1 | Eagle prediction 105415255 with correct synteny does not BLAST align to chicken. |
| CCAR2 | Goose prediction does not BLAST align to chicken. |
| CCDC106 | Falcon prediction (partial synteny with ZFN851) does not BLAST align to chicken |
| CCDC115 | Tit prediction 102103281 does not BLAST align to chicken |
| CCDC155 | Tit prediction (partial synteny with CD37 some distance away) does not BLAST align to chicken. |
| CCDC22 | Tit prediction 102110932 does not BLAST align to chicken |
| CCDC68 | Pigeon prediction (partial synteny with DYNAP and RAB27B) does not BLAST align to chicken |
| CD22 | Goose prediction (FFAR3 established synteny) does not BLAST align to chicken. |
| CD2BP2 | Tit prediction (synteny not established) has short BLAST alignment in chicken that does not reciprocally BLAST back to correct locus. |
| CD37 | Prediction 102105837 in tit does not BLAST align to chicken |
| CDK4 | Tit prediction 102103942 does not BLAST align to chicken |
| CFP | Tit prediction 102102913 does not BLAST align to chicken. Chicken prediction ENSGALG00000003236 is BAI2, not CFP. |
| CLIC1 | Kiwi prediction does not BLAST align to chicken. Chicken prediction ENSGALG00000021233 is CLIC3, not CLIC1 |
| COL5A3 | Tinamou prediction (synteny partial with OLFM2) does not BLAST align to chicken. |
| CTDSP2 | Ostrich prediction (with different synteny than human, but 95% protein alignment with CTDSP2 in lizard) has best BLAST alignments to CTDSP1 and CTDSP1L in chicken. |
| CYC1 | Tit and medium ground finch predictions do not BLAST align to chicken |
| DBP | Tit prediction 102103469 does not BLAST align to chicken |
| DCAF15 | Tit prediction does not BLAST align to chicken |
| DCC | Pigeon prediction (with synteny being different from human, but best BLAT hit with protein in lizard to correct DCC) does not BLAST align to chicken |
| DEDD2 | Eagle prediction with no synteny, but good alignment to DEDD2 in lizard, does not BLAST align to chicken. |
| DMPK | Tit prediction 102109823 does not BLAST align to chicken |
| DMWD | Tit prediction 102112782 does not BLAST align to chicken |
| DNAAF3 | Prediction 107307227 does not BLAST align to chicken |
| DTX3 | Tit prediction 102106148 does not BLAST align to chicken |
| EFNB3 | Tit prediction (synteny established by WRAP53, which is still missing in chicken) BLAST aligns to to EFBN1 (main + allele) |
| EHMT2 | Starling prediction (synteny established by ZBTB12) BLAST aligns to EHMT1 |
| ELAVL3 | Tit prediction 102106080 does not BLAST align to chicken |
| EMC10 | Tit prediction (synteny is different from human, but best protein blat in lizard is to EMC10 with correct synteny) does not BLAST align to chicken. |
| EMC4 | Avian predictions do not BLAST align to chicken. Previous chicken prediction 100857962 has been discontinued in Galgal5 |
| EMP3 | Prediction 106628800 from tit does not BLAST align to chicken |
| EPOR | Gallus gene reconstructed from SRA by Hron et al. 2015, does not BLAST align to chicken |
| ERF | Prediction 102110307 from tit does not align to chicken; prediction 106487876 from kiwi is incorrectly annotated, this ERF paralog is syntenic with ARHGEF1 |
| FAM3A | Pigeon prediction (synteny partial with G6PD) has best BLAST alignment to FAM3D |
| FAM57B | Tit prediction 102101566 does not BLAST align to chicken |
| FFAR3 | Duck prediction (106018249) aligns to chr31random_Scaffold14607; no synteny |
| FITM1 | Tit prediction 102107205 does not BLAST align to chicken |
| FKBPL | Prediction 102110619 from tit does not BLAST align to chicken; of note, this prediction shares very low %id with non-avian orthologs; no synteny info |
| FLT3LG | Prediction 106628898 from tit does not BLAST align to chicken |
| FOXH1 | Prediction 104300697 from downy woodpecker (and other birds) does not BLAST align to chicken |
| FOXP3 | Prediction 102110738 from tit does not BLAST align to chicken; of note, there is no synteny information for this prediction |
| GIPC1 | Turkey prediction 100546758 does not BLAST align to chicken |
| HAUS4 | Prediction 107307180 from quail does not BLAST align to chicken; of note this prediction shares very low %id with non-avian orthologs |
| HIPK4 | Manakin prediction 103762135 does not BLAST align to chicken |
| HIRIP3 | Tit prediction 102108916 does not BLAST align to chicken |
| HSPA12B | 770082 is likely a novel paralog HSPA12BL. Tit prediction does not BLAST align to chicken |
| INO80E | Tit prediction 102108731 does not BLAST align to chicken |
| IRF2BP1 | Golden Eagle Prediction (105414401) has correct partial synteny, but only BLAST aligns to IFR2BP1-like in chicken. |
| IRGC | Tit prediction (no synteny, but best alignment to lizard to model named IGRC with no synteny) does not BLAST align to chicken |
| KCNK6 | Prediction 105414438 in eagle does not BLAST align to chicken |
| LIN37 | Tit prediction does not BLAST align to chicken |
| MAP3K10 | Kiwi Prediction (106493720) with correct synteny maps to incorrect locus (MAP3K9) in chicken. |
| MBD6 | Ibis prediction does not BLAST align to chicken |
| MECP2 | Starling prediction BLAST aligns to METTL3, not MECP2, in chicken |
| METTL1 | Tit prediction 102103578 does not BLAST align to chicken |
| MRPS18B | Tit and Turkey predictions do not BLAST align to chicken. |
| NACC1 | Goose prediction 105414984 BLAST aligns to chicken NACC2. Previous chicken Galgal4 prediction (100858037) chrUn_AADN03018811:917-1783 100858037 has been removed and cannot be evaluated. |
| NDUFA3 | Tit prediction 102107821 does not BLAST align to chicken |
| NPHS1 | Prediction 106630934 in falcon does not BLAST align to chicken; of note, this prediction shares very low %id (~35%) with non-avian orthologs |
| NUMBL | Eagle prediction BLAST aligns to locus containing 421982. 421982 is incorrectly annotated, and is a likely paralog of LDLRAP1 that is present in Alligators with the same synteny. |
| PCP2 | Tit prediction 102101991 does not BLAST align to chicken |
| PDLIM2 | Tit prediction 102100383 does not BLAST align to chicken |
| PDZD4 | Predictions in tit (102102112) and starling (106863828) do not BLAST align to chicken |
| PET100 | Tit prediction 102101810 does not BLAST align to chicken |
| PFKFB1 | Goose prediction 105415371 BLAST aligns to chicken PFKB4. Previous chicken Galgal4 prediction 770873 has been removed and cannot be evaluated. |
| PKMYT1 | Prediction 106899925 in ruff does not BLAST map to chicken; of note, this ruff prediction has very low % id with non-avian orthologs; no synteny info available |
| PLCB3 | Prediction 106863536 in starling does not BLAST align to chicken |
| PLPPR2 | Prediction 108510285 from manakin and Hron et al., 2015 model reconstructed from chick SRA do not BLAST align to chicken. Of note: Hron et al., 2015 also reports that this gene is present in tit based on SRA reconstructions. |
| POLI | Goose prediction does not BLAST align to chicken. |
| PPP1R14A | Eagle prediction does not BLAST align to chicken |
| PPP1R16A | Peregrin falcon prediction 102049024 does not BLAST align to chicken |
| PRR12 | Tit prediction (no synteny, but best alignment to lizard to model named IGRC with no synteny) does not BLAST align to chicken |
| PRRC2A | Prediction 106629068 in tit does not BLAST align to chicken; of note, there is no synteny information for this prediction |
| PRRT1 | Tit prediction does not BLAST align to chicken. Turkey prediction is a misannotated unknown gene that is present in galgal4/5 |
| PRSS8 | Prediction 106628917 in tit does not BLAST align with chicken |
| PSPN | Prediction 106628809 in tit does not BLAST align to chicken |
| PTPN18 | Falcon prediction does not BLAST align to chicken |
| RAB2B | Prediction 106498859 in kiwi does not BLAST align to chicken; note: chicken prediction 100858328 is orthologous to a RAB11A-like in gator LOC102576372 |
| RAB27B | Goose prediction does not BLAST align to chicken. |
| RASIP1 | Tit prediction 102104192 does not BLAST align to chicken. Previous chicken Galgal4 prediction 431089 has been removed and cannot be evaluated. |
| RFX1 | Tit prediction 102114151 does not BLAST align to chicken |
| RPL13A | Tit prediction does not BLAST align to chicken, but see SLC17A7 |
| RPL18 | Tit and Tinamou probes do not BLAST align to chicken |
| RXRB | Goose and duck predictions do not BLAST align to chicken |
| S1PR5 | Prediction 106485711 in kiwi does not BLAST align to chicken |
| SALL2 | Prediction 107307026 in quail does not BLAST align to chicken |
| SAMD1 | Medium ground finch genomic segment (JH739919:2342646-2342722) does not BLAST align to chicken. Note: Eagle prediction 105415250 is DNM2. |
| SDR39U1 | Goose and Finch predictions do not BLAST align to chicken. |
| SERTAD1 | Ostrich (104572766) and Kea (104406943) predictions with correct synteny (PRX/ BLVRB also present) do not BLAST align to chicken. |
| SLC39A4 | Tit prediction 101919192 does not BLAST align to chicken |
| STARD6 | Duck prediction does not BLAST align to chicken |
| STX4 | Prediction 102089411 in pigeon does not BLAST align to chicken |
| SWSAP1 | Tit prediction 102100104 and chicken SRA reconstructed gene from Hron et al., 2015 do not BLAST align to chicken |
| SYT3 | Goose prediction does not BLAST align to chicken. |
| TGFBR3L | Tit prediction 102100511 does not BLAST align to chicken |
| THTPA | Prediction 102086719 in pigeon does not BLAST align to chicken; of note, this prediction has low %id with non-avian orthologs and there is no synteny info |
| TMED1 | Eagle prediction 105415254 does not BLAST to chicken |
| TMEM147 | Starling prediction BLAST aligns to unspecified loci in chicken |
| TMUB2 | Tit prediction 102103461 does not BLAST align to chicken |
| TOX4 | Tit prediction 102100210 does not BLAST align to chicken |
| TRMT1 | Starling prediction BLAST aligns to MAN2B1 |
| TSPAN31 | Several avian species have TSPAN31 (106628669; 106628669) with correct synteny. No nucleotide or protein BLAST alignments to chicken |
| TSR2 | Tit prediction 102104373 does not BLAST align to chicken |
| TSSK4 | Several species have models named TSSK4 (e.g. 104570659); synteny is all wrong, but models align to single exon of correct syntenic locus in alligator; models do not align to chicken |
| TTC5 | Prediction 102100756 in tit does not BLAST align to chicken; of note, synteny differs from non-avian organisms, but syntenic OSGEP and APEX1 are nearby on human chr19, suggesting local rearrangement |
| U2AF2 | Tit prediction does not BLAST align to chicken |
| WNK3 | Tit prediction 102104556 does not BLAST align to chicken |
| WRAP53 | Several bird species have WRAP53 with correct synteny (106863818; 105415231). Aquila prediction does not BLAST align to chicken. |
| YIPF2 | Aquila prediction 105415256 does not BLAST align to chicken. |
| ZNF574 | Tit prediction 102099855 and Turkey prediction do not BLAST align to chicken |
