# Supplementary material for: A New Chicken Genome Assembly Provides Insight into Avian Genome Structure
Source: G3 (Bethesda). 2016 Nov 14;7(1):109–17. doi: 10.1534/g3.116.035923 (PMC5217101; doi:10.1534/g3.116.035923)
Supplement: Supplementary file 27 [file 109TableS5.docx]

**Table S5.** Genes now present in Gallus_gallus-5.0 that were not predicted in Gallus_gallus-4.0. See methods for a description of search details.

| **Gene Name** | **Chr** | **Galgal5 gene prediction^** | **Orthology evidence*** |
| --- | --- | --- | --- |
| ASNA1 | chr30 | 100859771 | correct synteny |
| ATP5B | chr33 | 426673 | correct synteny |
| AVIL | chr3 | 50853 | correct synteny |
| B4GALT3 | chr25 | 107049101 and possibly 107051237 (ChrUn). Note: 418342 is likely a B4GALT3L paralog. | correct synteny partial |
| CALR | chr30 | 100859104 | correct synteny |
| CDK2 | chr33 | 100529062 | correct synteny |
| CFAP126 (aka C1orf192) | chr25 | 100859681 | correct synteny partial |
| COPZ1 | chr33 | 107055416 | correct synteny |
| ECSIT | chr30 | 100857880 | correct synteny |
| ERBB3 | chr33 | 693245 | correct synteny |
| ESYT1 | chr33 | 107055399 | correct synteny |
| EXOSC5 | chr32 | 100858931 | correct synteny partial |
| FARSA | chr30 | 100859604 | correct synteny |
| FBXL12 | chr30 | 396477 | correct synteny |
| FBXW9 | chr30 | 107057629 | correct synteny partial |
| GPR182 | chr33 | 100858910 | correct synteny |
| HNRNPUL1 | chr32/chrUn | 776596/107049202 | correct synteny |
| HOXC6 | chr33 | 100858368 | correct synteny |
| IKZF4 | chr33 | 101747801 | correct synteny |
| KCNH2 | chr2 | 100858122 | correct synteny |
| KLHL33 | chr28 already placed in galgal4 | 101750992 | incorrect synteny in all birds; reciprocal BLAST to correct locus in human/alligator |
| MIP | chr33 | 374124 | correct synteny partial |
| NOS3 | chr2 | 100858754 | correct synteny |
| PRIM1 | chr33 | 426646 | correct synteny partial |
| PRPH | chr33 | 101748212 | correct synteny |
| RASAL3 | chr30 | 101747651. Note: 426166 is incorrectly annotated as RASAL3 and is actually WIZ | correct synteny |
| RDH5 | chr33 | 395452 | correct synteny |
| RPS26 | chr33 | 100857770 | correct synteny |
| S100A10 | chr25 | 396506 | correct synteny partial |
| SCNM1 | chr25 | 100857482 | correct synteny |
| SDHC | chr25 | 100859641 | correct synteny |
| SEMA4C | chr22 | 107057370 | correct synteny |
| SMARCC2 | chr33 | 101747634 | correct synteny |
| SOX12 | chr20 | 777179 | correct synteny |
| STAP2 | chr28 | 107055321 | correct synteny |
| TARBP2 | chr33 | 107055413 | correct synteny |
| TBX21 | chr27 | 100858002 | correct synteny partial |
| TGFB1 | chr32 | 100873157 | correct synteny |
| TNPO2 | chr30 | 107057623 | correct synteny |
| TTC9C | chr5 | 768624 | correct synteny |
| YIF1B | chr32_random | 107049208 | no synteny; reciprocal BLAT to correct locus in alligator |
| ZBTB39 | chr33 | 100858734 | correct synteny |
| ADCY6 | chrUn | 107049877/107050028 | correct synteny partial |
| ADGRL1 aka LPHN1 | chrUn | 107051501/107051276 (partials) | no synteny; reciprocal BLAST to correct protein in several species |
| AKT2 | chrUn | 373973 | incorrect synteny; reciprocal BLAST to correct protein in several species |
| ANKRD39 | chrUn | 107057371. Note: 425706 is likely CNNM4 or CNNM3. | correct synteny |
| APEX1 | chrUn | 100431102 (discontinued) | correct synteny partial |
| ARF3 | chrUn | 107049507 | correct synteny |
| ATAT1 | chrUn | 107051422 | no synteny; reciprocal BLAST to correct protein in several species |
| ATG4D | chrUn | 107051455 | no synteny; reciprocal BLAT to correct locus in alligator and/or human |
| BBS1 | chrUn | 107051131 | correct synteny |
| BCAP31 | chrUn | 100857815 | incorrect synteny; reciprocal BLAT to correct locus in allligator and/or human |
| C12orf44 aka ATG101 | chrUn | 100859845 | correct synteny |
| C19orf52 | chrUn | 107051509 | correct synteny |
| CACNA1A | chrUn | 107049833 | correct synteny |
| CACNB3 | chrUn | 107050029 (misannotated) | correct synteny partial |
| CACNG7 | chrUn | 107050634 | no synteny; reciprocal BLAT to correct locus in alligator and/or human |
| CACNG8 | chrUn | 107050872 | incorrect synteny; reciprocal BLAT to correct locus in allligator and/or human |
| CAMSAP3 | chrUn | 107050537. Note: Other birds have CAMSAP-like gene with different synteny from human | no synteny; reciprocal BLAT to correct locus in alligator and/or human |
| CCDC120 | chrUn | 107049521 | correct synteny |
| CCDC130 | chrUn | 107049833 | correct synteny |
| CCDC65 | chrUn | 100857346. Note: 429591 is discontinued. | correct synteny |
| CCDC97 | chrUn | 101748852 | correct synteny |
| CCNT1 | chrUn | 100859215 | correct synteny partial |
| CHD8 | chrUn | 107050220/100857358/107050371 | no synteny; reciprocal BLAST to correct protein in several species |
| CLASRP | chrUn | 107050286 | no synteny; reciprocal BLAT to correct locus in alligator and/or human |
| CLEC17A | chrUn (segment NT_466375) | 426106 | no synteny, but partial synteny in falcon |
| CLPP | chrUn | 107051000 | no synteny; reciprocal BLAT to correct locus in alligator and/or human |
| CSAD | chrUn | 426184 | correct synteny partial |
| CSRNP2 | chrUn | 100859835 | correct synteny |
| CYTH2 | chrUn | 107050873 | incorrect synteny; reciprocal BLAT to correct locus in allligator and/or human |
| DAZAP2 | chrUn | 107051549 | no synteny; reciprocal BLAT to correct locus in alligator and/or human |
| DCTN2 | chrUn | 395587 | no synteny; reciprocal BLAT to correct locus in alligator and/or human |
| DDIT3 | chrUn | 100857832 | correct synteny partial |
| DNAJB1 | chrUn | 107049075/100858808 | correct synteny (107049075 )/no syteny (100858808) |
| DNM2 | chrUn | 107051521 | no synteny; reciprocal BLAT to correct locus in alligator and/or human |
| DPF1 | chrUn | 395352 | no synteny; reciprocal BLAT to correct locus in alligator and/or human |
| ETFB | chrUn | 107051275 | no synteny; reciprocal BLAT to correct locus in alligator and/or human |
| EXOSC4 | chrUn | 107050971 | no synteny; reciprocal BLAST to correct chromosomal location |
| FKBP11 | chrUn | 107049506 | correct synteny partial |
| FLOT1 | chrUn | 101751397 | correct synteny partial |
| FMNL3 | chrUn | 100857380 | correct synteny partial |
| FUS | chrUn | 414144 | no synteny; reciprocal BLAT to correct locus in alligator and/or human |
| FUZ | chrUn | 101748520 | correct synteny |
| GABBR1 | chrUn | 107050785 | no synteny; reciprocal BLAT to correct locus in alligator and/or human |
| GATA1 | chrUn | 107050548/396450 | correct synteny partial |
| GEMIN7 | chrUn | 107049538 | correct synteny partial (reverse orientation) |
| GNG3 | chrUn | 107051345 | no synteny; reciprocal BLAT to correct locus in alligator and/or human |
| GNL3L | chrUn | 107049485/101748854 | correct synteny partial |
| GPAA1 | chrUn | 107050693 | no synteny; reciprocal BLAT to correct locus in alligator and/or human |
| GPKOW | chrUn | 107050739 | no synteny; reciprocal BLAT to correct locus in alligator and/or human |
| GRIK5 | chrUn | 107051232. Note: 107051379 is misannotated and is lkely GRIK4. | no synteny; reciprocal BLAST to correct protein in several species |
| GTF2F1 | chrUn | 107051030/107051351 (partials) | no synteny; reciprocal BLAT to correct locus in alligator |
| HCFC1 | chrUn | 107051408 | no synteny; reciprocal BLAT to correct locus in alligator and/or human |
| HDAC6 | chrUn | 107050547 | correct synteny partial |
| HIF3A | chrUn | 107049602 | correct synteny |
| HOOK2 | chrUn | 107050016 | correct synteny |
| HSPBP1 | chrUn | 100858988 | correct synteny partial |
| ILF3 | chrUn | 100858655 | no synteny; reciprocal BLAST to correct protein in several species |
| IPO4 | chrUn | 101750157 | correct synteny |
| JOSD2 | chrUn | 107051183 (misannoated as JOS2) | no synteny; reciprocal BLAT to correct locus in alligator and/or human |
| JUNB | chrUn | 107050017 | correct synteny |
| KANSL2 | chrUn | 426401 | correct synteny partial |
| KCNA7 | chrUn | 107050569 | no synteny; reciprocal BLAT to correct locus in alligator and/or human |
| KEAP1 | chrUn | 100858752 (misannotated) | correct synteny partial |
| KHSRP | chrUn | 374140 | no synteny; reciprocal BLAT to correct locus in alligator and/or human |
| KMT5C | chrUn | 101748081 | correct synteny |
| KRI1 | chrUn | 107050624 | no synteny; reciprocal BLAT to correct locus in alligator and/or human |
| L1CAM | chrUn | 396059 | incorrect synteny; reciprocal BLAT to correct locus in allligator and/or human |
| LDLR | chrUn | 395103 | correct synteny |
| LENG8 | chrUn | 101748015 | incorrect synteny; reciprocal BLAT to correct locus in allligator and/or human |
| LETMD1 | chrUn | 107050878 (misannotated)/101748057 | correct synteny |
| LIN7B | chrUn | 107050779. Note: 421608 is likely LIN7C | no synteny; reciprocal BLAST to correct protein in several species |
| LRRC4B | chrUn | 100857901 | no synteny; reciprocal BLAST to correct chromosomal location |
| LSMD1 aka NAA38 | chrUn | 426258 /107054771 (identical sequences) | incorrect synteny; reciprocal BLAT to correct locus in allligator and/or human |
| MAP2K7 | chrUn | 107050212/107050551 (partials) | no synteny; reciprocal BLAST to correct protein in several species |
| MAP4K1 | chrUn_AADN03014578 | 107055388 and chr32random_Scaffold17238 | correct synteny |
| MARK2 | chrUn | 107049628 | correct synteny partial |
| MARS | chrUn | 425613 | correct synteny |
| MBOAT7 | chrUn | 107050678 | correct synteny partial |
| METTL21B | chrUn_AADN03026913 | 107051068 | correct synteny |
| METTL3 | chrUn | 107050676 | no synteny; reciprocal BLAST to correct protein in several species |
| MMP14 | chrUn | 107051257 and 426253 | Prediction from Hron et al., 2015 aligns to scaffold that contains MRPL52 providing evidence of correct synteny. Second hit is to model that is MMP15 (see Lovell et al., 2015, Table S2) |
| MPZ | chrUn | 100859605 | correct synteny |
| MRPL52 | chrUn | 101751307 (Unannotated) | Prediction from Hron et al., 2015 aligns to unamed model 101751307 |
| MYBPC2 | chrUn | 425457 | no synteny; reciprocal BLAT to correct locus in alligator and/or human |
| NDUFB7 | chrUn | 107050898 | no synteny; reciprocal BLAT to correct locus in alligator and/or human |
| NKPD1 | chrUn | 107050647 | no synteny; reciprocal BLAST to correct protein in several species |
| NOSIP | chrUn | 107050704 | no synteny; reciprocal BLAST to correct protein in several species |
| NR4A1 | chrUn | 100857380 | correct synteny partial |
| NRXN2 | chrUn | 101751890 (unannotated) | correct synteny partial |
| OS9 | chrUn | 107050049 | no synteny; reciprocal BLAT to correct locus in alligator and/or human |
| OTUD5 | chrUn | 100858279 | no synteny; reciprocal BLAST to correct protein in several species |
| PIH1D1 | chrUn | 107050350 | no synteny; reciprocal BLAST to correct protein in several species |
| PLD3 | chrUn and chr32 | 107049989. Also chr32random_Scaffold1336 (no model) with PRX providing systenic context. | correct synteny |
| POU2F2 | chrUn | 107049968 | no synteny; reciprocal BLAST to correct protein in several species |
| POU6F1 | chrUn | 107049477 | correct synteny |
| PPOX | chrUn | 107051238 | correct synteny partial |
| PPP1R10 | chrUn | 100859302/107051223/107051208/107050600/107051271 | no synteny; reciprocal BLAST to correct protein in several species |
| PPP1R12C | chrUn | 107049646 | correct synteny partial |
| PPP1R9B | chrun | 100859037 (misannotated). Note: 395762 is PPP1R9BL paralog. | correct synteny |
| PPP4C | chrUn | 101748916 (misannotated as extensin-like) | no synteny; reciprocal BLAST to correct protein in several species |
| PPP5C | chrUn | 107050972 | no synteny; reciprocal BLAST to correct protein in several species |
| PRKCG | chrUn | 107050869 | no synteny; reciprocal BLAST to correct protein in several species |
| PRMT5 | chrUn | 101750306 | no synteny; reciprocal BLAST to correct protein in several species |
| PRPF31 | chrUn | 107050765 | correct synteny partial |
| PRX | chrUn | 107049204 | correct synteny |
| PSMD8 | chrUn | 107049205 | incorrect synteny; reciprocal BLAT to correct locus in allligator and/or human |
| QPRT | chrUn | 107051294 | no synteny; reciprocal BLAT to correct locus in alligator and/or human |
| RAB4B | chrUn | 107050243 | correct synteny partial |
| RABGGTA | chrUn | 100858474 and chrUn_Scaffold15355 | no synteny (100858474); correct synteny partial (chrUn_Scaffold15355) |
| REC8 | chrUn | 101751846 | correct synteny |
| RELB | chrUn | 395656 | no synteny; reciprocal BLAT to correct locus in alligator and/or human |
| RENBP | chrUn | 107050161 | no synteny; reciprocal BLAT to correct locus in alligator and/or human |
| RING1 | chrUn | 100857381 (misannotated) | no synteny; reciprocal BLAST to correct protein in several species |
| RNASEH2A | chrUn | 107050018 | correct synteny |
| RNF31 | chrUn | 107051362 | no synteny; reciprocal BLAST to correct protein in several species |
| RUVBL2 | chrUn | 107051177 | no synteny; reciprocal BLAST to correct protein in several species |
| SAE1 | chrUn | 101750090 | correct synteny partial |
| SCAF1 | chrUn | 107051499 (misannotated) | no synteny; reciprocal BLAT to correct locus in alligator and/or human |
| SCN1B | chrUn | 107051305 | no synteny; reciprocal BLAST to correct protein in several species |
| SETD1A | chrUn | 107051142/107050768 (101751721 is discontinued) | no synteny; reciprocal BLAT to correct locus in alligator and/or human |
| SHANK1 | chrUn | 107051246 (unnanotated) | no synteny; reciprocal BLAST to correct protein in several species |
| SHMT2 | chrUn | 107051323 | no synteny; reciprocal BLAST to correct protein in several species |
| SIPA1L3 | chrUn | 107051253/107050761. Note: 101749913 is likely SIPA1 | no synteny; reciprocal BLAST to correct protein in several species |
| SLC11A2 | chrUn | 751817 | no synteny; reciprocal BLAT to correct locus in alligator and/or human |
| SLC17A7 | chrUn | 107050637 and 100859053 (chrUn_Scaffold8980) | correct synteny |
| SLC35A2 | chrUn | 107050985 | no synteny, but syntenic with tit; reciprocal BLAT to correct locus in alligator |
| SLC39A7 | chrUn | 107050736 | no synteny; reciprocal BLAT to correct locus in alligator and/or human |
| SLC44A2 | chrUn | 107050443/101748239/107051174 | correct synteny |
| SMC1A | chrUn | 395187 | no synteny; reciprocal BLAT to correct locus in alligator and/or human |
| SMG9 | chrUn | 107051019 | no synteny; reciprocal BLAT to correct locus in alligator and/or human |
| SNRNP70 | chrUn | 107051070 | no synteny; reciprocal BLAST to correct protein in several species |
| SPINT2 | chrUn | 107051508 | no synteny; reciprocal BLAST to correct protein in several species |
| SPRYD3 | chrUn | 100858430 | correct synteny partial |
| SPTBN4 | chrUn | 101749113 | no synteny; reciprocal BLAT to correct locus in alligator and/or human |
| SRPK3 | chrUn | 107049821 | correct synteny |
| SSR4 | chrUn | 107049822 | correct synteny |
| STAC3 | chrUn | 107050875/107050608/107055620/107050760/107050387/107050250 | no synteny; reciprocal BLAST to correct protein in several species |
| STAT6 | chrUn | 100859196 | no synteny; reciprocal BLAT to correct locus in alligator and/or human |
| STIP1 | chrUn | 101748085 | correct synteny partial |
| STRN4 | chrUn | 107050597 | no synteny; reciprocal BLAST to correct protein in several species |
| TECR | chrUn | 107050163. Note: 424491 is likely a TECRL paralog | correct synteny |
| TFCP2 | chrUn | 378900 | correct synteny |
| TFE3 | chrUn | 107049522 | correct synteny |
| TFPT | chrUn | 107050764 | correct synteny |
| TINF2 | chrUn | 107049947 | correct synteny |
| TNNT1 | chrUn | 396009 | correct synteny partial |
| TRMT112 | chrUn | 100858508 | no synteny; reciprocal BLAT to correct locus in alligator and/or human |
| TRPT1 | chrUn | 107049849 | correct synteny |
| TSFM | chrUn | 107050777 | incorrect synteny; reciprocal BLAT to correct locus in allligator and/or human |
| TSPYL2 | chrUn | 107050801 | no synteny, but syntenic with tit; reciprocal BLAT to correct locus in alligator |
| UBA1 | chrUn | 107050431 107051050 | no synteny; reciprocal BLAST to correct protein in several species |
| UBL4A | ChrUn_JH375752 | 107050156 | correct synteny |
| UBL5 | chrUn | 107057622 | correct synteny |
| USP39 | chrUn | 100859000 | no synteny; reciprocal BLAT to correct locus in alligator and/or human |
| WNT1 | chrUn | 396160 | correct synteny partial |
| ZNF385A | chrUn | 101749643 | correct synteny |
| ZNF653 | chrUn | 107057618 | correct synteny |
| ZNF668 | chrUn | 101748630 | no synteny; reciprocal BLAST to correct protein in several species |
| ZNF865 | chrUn | 107050672 | correct synteny partial |
| AGAP2 | chrUn_Scaffold25997 | No Prediction | no synteny; reciprocal BLAST to correct protein in several species |
| ARL2 | chrUn_Scaffold14474 not 769546 | No Prediction | incorrect synteny; reciprocal BLAT to correct locus in allligator and/or human |
| ASPDH | chrUn_Scaffold8802 | No Prediction | no synteny; reciprocal BLAST to correct protein in several species |
| AXL | chrUn_Scaffold8307 | No Prediction | no synteny; reciprocal BLAST to correct protein in several species |
| BLVRB | chrUn_Scaffold18087 | No Prediction. Found with BLAST of probe from Hron et al., 2015 | no synteny; reciprocal BLAST to correct protein in several species |
| C19orf53 | chrUn_Scaffold2757 | No Prediction | correct synteny partial |
| C6orf136 | chrUn_Scaffold25966 | No Prediction | no synteny; reciprocal BLAST to correct protein in several species |
| CATSPERB | chr5 | No Prediction | correct synteny |
| CDK16 | chrUn_Scaffold14982 | No Prediction | correct synteny partial |
| CYP27B1 | chrUn_Scaffold22566 | No Prediction | no synteny; not possible due to lack of prediction |
| DCAF11 | chrUn_Scaffold19972 | No Prediction | no synteny; reciprocal BLAST to correct protein in several species |
| FAM50A | chrUn_Scaffold4774 | No Prediction | correct synteny partial |
| FKBP2 | chrUn_Scaffold12164 hit with tit probe | No Prediction | correct synteny partial |
| FLRT1 | chrUn_Scaffold9859 | No Prediction | correct synteny partial |
| GPT | chrUn_Scaffold13775 | No Prediction | no synteny; reciprocal BLAST to correct protein in several species |
| GRM6 | chrUn_Scaffold25914 | No Prediction | no synteny; reciprocal BLAT to correct locus in alligator and/or human |
| GUCY2D | chrUn_Scaffold16759 | No Prediction | correct synteny partial |
| KDMB | chrUn (Segment NT_469681) | No Prediction | no synteny; reciprocal BLAST to correct protein in several species |
| KIF5A | chrUn_Scaffold3342 | No Prediction | correct synteny partial |
| LENG1 | chrUn_Scaffold25896 | No Prediction | no synteny; reciprocal BLAST to correct protein in several species |
| LMTK3 | chrUn_Scaffold13988 found with an ostrich probe blast | No Prediction | no synteny; not possible due to lack of prediction |
| LTBP4 | chrUn_Scaffold9746 | No Prediction | no synteny; reciprocal BLAST to correct protein in several species |
| NOP9 | chrUn_Scaffold8074 chrUn_Scaffold15355 | No Prediction | correct synteny partial |
| OPLAH | chrUn_Scaffold13222 | No Prediction | correct synteny partial |
| OSGEP | chrUn_Scaffold13034 | No Prediction | correct synteny partial |
| PHF8 | chrUn_Scaffold16543 | No Prediction | no synteny; reciprocal BLAST to correct protein in several species |
| PIP4K2C | chrUn_Scaffold3342 | No Prediction | no synteny; reciprocal BLAST to correct protein in several species |
| PPP1R18 | chrUn_Scaffold13807 | No Prediction | correct synteny |
| PQBP1 | chrUn_Scaffold5284 and chrUn_Scaffold5467 | No Prediction | correct synteny partial |
| PRKCSH | chrUn_Scaffold8470 | No Prediction | incorrect synteny, but same as tit; reciprocal BLAT to correct locus in alligator and/or human |
| PSENEN | chrUn_Scaffold9035 | No Prediction | no synteny; reciprocal BLAST to correct protein in several species |
| RCE1 | chrUn_Scaffold13431 | No Prediction | no synteny; reciprocal BLAST to correct protein in several species |
| SRCAP | chrUn_Scaffold23673 | No Prediction | no synteny; reciprocal BLAST to correct protein in several species |
| STX1B | chrUn_Scaffold18729 | No Prediction | no synteny; reciprocal BLAST to correct protein in several species |
| THOC6 | chrUn_Scaffold25922 | No Prediction | no synteny; reciprocal BLAST to correct protein in several species |
| TRAPPC1 | chrUn_Scaffold16759 | No Prediction | correct synteny partial |
| UXT | chrUn_Scaffold14982 | No Prediction | correct synteny partial |
| WDR45 | chrUn_Scaffold23173 | No Prediction | no synteny; reciprocal BLAST to correct protein in several species |
| XAB2 | chrUn_Scaffold16047 | No Prediction | no synteny; reciprocal BLAST to correct protein in several species |

| ^ Unannotated models were found by BLAST searches of Gallus_gallus-5.0 with synteny  verified avian and non-avian nucleotide sequences. |  |
| --- | --- |
| * Evidence based on correct synteny with orthologs in human/lizard or reciprocal  best BLAST alignments to correct nucleotide or protein. | |
