# Supplementary material for: A New Chicken Genome Assembly Provides Insight into Avian Genome Structure
Source: G3 (Bethesda). 2016 Nov 14;7(1):109–17. doi: 10.1534/g3.116.035923 (PMC5217101; doi:10.1534/g3.116.035923)
Supplement: Supplementary file 28 [file 109TableS6.docx]

**Table S6**. Genes unplaced in Gallus_gallus-4.0 that now have improved predictions and/or chromosomal placements in Gallus_gallus-5.0

| **Gene** | **Gallus_gallus-4.0 location** | **Gallus_gallus-5.0 location** | **Gallus_gallus-5.0 Prediction^** | **Orthology Evidence*** |
| --- | --- | --- | --- | --- |
| ABP1 | chr2:338032-339566 | chr2 | 100858159 | correct synteny |
| ADAMTS4 | chr25_AADN03010928_random:406-817 | chr25 | 100858999 | correct synteny |
| AKAP8L | W_random | chr30_random | 776856 | correct synteny partial |
| ANKRD52 | Un_random | chr33 | 426469 | correct synteny |
| BAZ2A | chrUn_AADN03019662:69-5,027 | chr33 | 101747779 | correct synteny |
| BLOC1S3 | chrUn_random | chr23random_Scaffold3161 | 426500 | no synteny; reciprocal BLAST to correct protein in several species |
| BORCS6 (aka C17orf59) | chr3 | chr3 | 101749651 (unannotated). Note: 421818 has been discontinued. | incorrect synteny; reciprocal BLAT to correct locus in allligator and/or human |
| C19ORF43 | chrUn_AADN03025224:726-806 | chr30 | 107057624 | correct synteny |
| C1QL4 | Un_random | chr33 | 100859113 | correct synteny partial |
| C2/CFB | chrUn_AADN03013857:158-457; chrUn_AADN03019114:589-801; chrLGE22C19W28_N03011248_random:108-1766; chrUn_AADN03025195:841-945 | chr23 | 419574 | incorrect synteny; reciprocal BLAST to correct protein in several species |
| CCDC189 (aka C16orf93) | chr17 | chr17 | 101750428 | incorrect synteny; reciprocal BLAST to correct protein in several species |
| CELA1 | Un_random | chr33 | 425200 and 107055420 | correct synteny partial (possible duplication) |
| CNN1 | chr27_AADN03011039_random:0-344; chrUn_AADN03020556:249-969 | chr30 | 396522 | correct synteny |
| COQ10A | Un_random | chr33 | 770970 | correct synteny |
| CPNE5 | chrUn_AADN03019059:71-504; chrUn_AADN03012208:442-504 | chr26 | 101749227/101748755 (partial). Note: 417690 is CPNE8 | correct synteny partial |
| CYP21A2 | chr17 | chr16 | 429828 | Synteny difficult to evaluate to define due to adjacent paralogs; may not be ortholog of human CYP21A2 |
| DNAJC22 | Un_random | chr33 | 430880 | correct synteny |
| FAM171A2 | chrUn_AADN03012606:667-882 | chr27 | 101747823 | synteny correct partial |
| GALNT6 | Un_random | chr33 | 429501 | correct synteny partial |
| GLI1 | Un_random | chr33 | 396045 | correct synteny partial |
| HOXC10 | Un_random | chr33 | 770471 | correct synteny |
| HOXC11 | Un_random | chr33 | 430698 | correct synteny |
| HOXC12 | Un_random | chr33 | 429400 | correct synteny |
| HOXC13 | Un_random | chr33 | 771955 | correct synteny |
| HOXC9 | Un_random | chr33 | 425723 | correct synteny |
| IMP4 | chrUn_AADN03017333:351-2306 | chr9 | 100857200 | incorrect synteny; reciprocal BLAT to correct locus in allligator and/or human |
| LYSMD1 | chrUn_JH376257:2554-4778 (Chicken NM_001105064.1 appears to be incorrectly annotated as NDNL2) | chr25 | 100857442 | correct synteny |
| MPND | chr28:1307135-1307276 | chr28 | 100858729 | correct synteny partial |
| MVP | chr28 | chr28 | 420049 | incorrect synteny; reciprocal BLAST to correct protein in several species |
| MYL6 | chr15:6028637-6031433; chr15:6028637-6031433 | chr33 | 100996929 | correct synteny partial |
| MYO1A | Un_random | chr33 | 396072 | correct synteny partial |
| NRSN2 | chr27:3902992-3906192 | chr20 | 771790 | correct synteny |
| OLFM2 | chrUn_JH375781:481-481 | chr30 | 107057621 | correct synteny |
| OSBPL7 | chrUn_JH375698:9480-11779 | chr27 | 100857443 | correct synteny |
| PAN2 | Un_random | chr33 | 429172 | correct synteny |
| PIN1 | chrUn_JH375781:163-163 | chr30 | 100859153 | correct synteny |
| PTGES3 | E22C19W28_E50C23 | chr33 | 100859133 | correct synteny |
| R3HDM2 | Un_random | chr33 | 426509 | correct synteny partial |
| RAB5B | chrW_JH375234_random:36978-37246 | chr33 | 100529061 | correct synteny |
| RBMS2 | 1 exon: chrUn_AADN03012516:11-226 (same as medium ground finch) | chr33 | 107055400 | correct synteny partial |
| RNF41 | Un_random | chr33 | 426468 | correct synteny |
| RYR1 | chrUn_AADN03019159:1652-3159 | chr32 | 396112 | correct synteny |
| S100A11 | chr25 | chr25 | 396075 | no synteny; reciprocal BLAT to correct locus in allligator and/or human |
| SHD | chrUn_AADN03013343:20-1569 | chr28 | 107055336 | correct synteny |
| SKIV2L | Un_random | chr26 | 421171 | correct synteny partial |
| SMUG1 | Un_random | chr33 | 431025 | correct synteny |
| SP1 | Un_random | chr33 | 395303 | correct synteny |
| SPATS2 | E22C19W28_E50C23 | chr33 | 426663 | correct synteny |
| SUOX | chrUn_AADN03013690:827-954 | chr33 | 107055404 | correct synteny |
| SUPT5H | Un_random | chr32 | 426493 | correct synteny partial |
| TBKBP1 | chr2:39181864-39182503 | chr27 | 430679 | correct synteny |
| TMEM106C | Un_random | chr33 | 426186 | correct synteny |
| VDR | Un_random | chr33 | 395988 | correct synteny |
| WDR49 | chr9 | chr9:20732872-20745259 | none; found by BLAST search with ostrich prediction 104140055 | correct synteny |
| WDR83OS | Un_random | chr30 | 777443 | correct synteny |
| AP1M2 | chrUn_AADN03012371:21-1769 | chrun | 107050442 | correct synteny |
| ASB16 | chrUn_AADN03025157:335-490 (Chicken); JH740174:9459-12596 (Medium Ground Finch) | chrun | 107049519 | correct synteny |
| ATP1A3 | chrUn_AADN03015791:644-796 | chrun | 396467 | no synteny; reciprocal BLAT to correct locus in allligator and/or human |
| ATP4A | chr25_JH375218_random:257-1953 | chrun | 107050629 (partial annotation) | no synteny; reciprocal BLAT to correct locus in allligator and/or human |
| ATXN7L3 | chrUn_AADN03025157:335-490 (Chicken); JH740174:9459-12596 (medium ground finch) | chrun | 107049520 | correct synteny |
| BCKDK | chrUn_AADN03012751:528-620; chrUn_AADN03026431:2869-3130 | chrun | 107051411 (misannotated) | no synteny; reciprocal BLAT to correct locus in allligator and/or human |
| BRD4 | Un_random | chrun | 100859056. Note: 430442 is PGLS | correct synteny |
| C19ORF47 | chrUn_AADN03024111:1019-1105; chrUn_AADN03024111:1019-1105 | chrun | 107049161 | corrrect synteny |
| C5AR1 | Un_random | chrun | 430484 | correct synteny partial |
| CACNA1F | chrUn_AADN03025195:27-981 | chrun | 429167 | no synteny; reciprocal BLAT to correct locus in allligator and/or human |
| CHD3 | chrUn_AADN03013648:45-116; chrUn_AADN03026359:2494-26669; chrUn_JH375715:5658-5866 | chrun | 107050721/107050448. Note: Previous galgal4 hitrs were to CHD8. | no synteny; reciprocal BLAT to correct locus in allligator and/or human |
| CIC^ | chrUn_AADN03021654:981-2514 | chrun | 107050718 | incorrect synteny; reciprocal BLAT to correct locus in allligator and/or human |
| CNOT3 | chrUn_AADN03025857:56-717 | chrun | 101748190 | no synteny; reciprocal BLAT to correct locus in allligator and/or human |
| COL1A1 | chrUn_JH375683:748-834; chrUn_JH375731:1132-3965 | chrun | 395532 | no synteny; reciprocal BLAT to correct locus in allligator and/or human |
| CYP4F22 | chrUn_AADN03022412:183-531; chrUn_AADN03027099:527-531 | chrun | 100859277 | correct synteny partial |
| DHX16 | chrUn_AADN03019848:1084-1293 | chrun/chrUn_Scaffold13807 | 107051018/non-predicted region | no synteny; reciprocal BLAST to correct protein in several species |
| ESPL1 | Un_random | chrun | 425536 | correct synteny partial |
| ETHE1 | Un_random | chrun | 426450 | no synteny; reciprocal BLAT to correct locus in allligator and/or human |
| FAM58A | chrUn_AADN03015519:1226-1300; chrUn_AADN03012069:1-81 | chrun | 107050366/107049631/107051120 | incorrect synteny; reciprocal BLAT to correct locus in allligator and/or human |
| FKRP | Un_random | chrun | 431512 | incorrect synteny; reciprocal BLAT to correct locus in allligator and/or human |
| GRIPAP1 | chrUn_JH375618:285-1702 | chrun | 101751935 | correct synteny |
| IDH3G | chrLGE22C19W28_E50C23:501345-503938 | chrun | 101751023. Note: 100859355 not in galgal5. | correct synteny |
| ITIH6 | chrUn_AADN03024263:765-921 | chrun | 430871 | correct synteny partial |
| KAT8 | chrUn_AADN03024263:765-920 | chrun | 107050398 | no synteny; reciprocal BLAT to correct locus in allligator and/or human |
| LIG1 | E64 | LGE64 | 430516 | incorrect synteny; reciprocal BLAT to correct locus in allligator and/or human |
| MCOLN1 | Un_random | chrun | 426164 | correct synteny partial |
| MFSD5 | Un_random | chrun | 777286 | correct synteny partial if conidering a short hit to RARG |
| NEDD8 | chrUn_AADN03026185:23-81; chrUn_AADN03024318:77-81 | chrun | 100858776/101747652 | correct synteny partial |
| OTUB1 | chrUn_AADN03014390:2380-3176 | chrun | 777320 | correct synteny |
| PGLYRP2 | chrUn_AADN03018508:114-185 | chrun | 693263 | correct synteny partial |
| PORCN | chrUn_AADN03019239:15-1169; chrUn_AADN03019325:266-834 | chrun | 751620/107050792 | no synteny; reciprocal BLAT to correct locus in allligator and/or human |
| PPP1R37 | chrUn_AADN03011330:382-483; chrUn_AADN03022638:723-872; chrUn_AADN03026805:1522-1620 | chrun | 107049538 | correct synteny partial |
| PSMB5 | chr2:36409886-36411853 | chrun | 396003 | no synteny; reciprocal BLAT to correct locus in allligator and/or human |
| RTN3 | chrUn_AADN03019159:1652-3159; chrUn_AADN03021284:1120-1210; chr3:36518946-36519211; chrUn_JH375817:1033-1200; chr3:36503669-36503819; chrUn_AADN03024501:967-1086 | chrun | 431037 | correct synteny partial |
| SGCA | chrUn_JH375682:636-701 | chrun | 100859263 | correct synteny |
| SHKBP1 | chrUn_AADN03015434:3-593 | chrun | 101747244 | no synteny; reciprocal BLAST to correct protein in several species |
| SYMPK | chrUn_AADN03021042:1085-1201 | chrun | 107050902 | no synteny; reciprocal BLAT to correct locus in allligator and/or human |
| TAZ | chr27_JH375221_random:1306-1871 | chrun | 107049523 | incorrect synteny; reciprocal BLAT to correct locus in allligator and/or human |
| TCF4 | chrUn_AADN03013408:6304-8404 | chrZ | 768612 | incorrect synteny; reciprocal BLAT to correct locus in allligator and/or human |
| TPPP2 | Z | chrZ | 427270 | incorrect synteny; reciprocal BLAT to correct locus in allligator and/or human |
| TYK2 | Un_random | chrun | 430113 | no synteny; reciprocal BLAT to correct locus in allligator and/or human |
| VARS | chrUn_AADN03015895:639-707 | chrun | 107050659. Note: 107049913 is VARS2 | no synteny; reciprocal BLAT to correct locus in allligator and/or human |
| ZBTB12 | chrUn_AADN03024216:2705-2869 | chrun | 107050860 | no synteny; reciprocal BLAT to correct locus in allligator and/or human |
| ZNF646 | chrUn_AADN03024083:308-532 | chrun | 101749845 | no synteny or incorrect synteny, a myosin related gene is predicted next to it but it does not blast to related myosin proteins |
| ACIN1 | chrUn_AADN03011814:1163-1411 | chrUn_Scaffold26015. Note: chrUn_AADN03011814:1163-1411 not in galgal5 | no prediction | no synteny; reciprocal BLAST to correct protein in several species |
| COL11A2 | chrUn_AADN03012305:1423-1530 | chrUn_Scaffold25892 | no prediction | no synteny; reciprocal BLAST to correct protein in several species |
| CPSF1 | chrUn_AADN03017637:744-3833 | chrUn_Scaffold12700/chrUn_Scaffold18080/chrUn_Scaffold11502/chrUn_Scaffold5043. Note: chrUn_AADN03017637:744-3833 was to actually CPSF1. | no prediction | no synteny; reciprocal BLAST to correct protein in several species |
| IQSEC2 | chrUn_JH376257:7155-8434 (no model) | chrUn_Scaffold25972 | no prediction | no synteny; reciprocal BLAST to correct protein in several species |
| LSM2 | chrUn_AADN03015605:1100-1168 | chrUn_Scaffold21626 | no prediction | no synteny; reciprocal BLAST to correct protein in several species |
| MED25 | chrUn_AADN03025011:46-745 | chrUn_Scaffold15178/chrUn_Scaffold10101 (partials) | no prediction | no synteny; reciprocal BLAST to correct protein in several species |
| NFE2 | chrUn_JH375657:293-378 | chr33 | no prediction (chr33:1166292-1166377) | correct synteny |
| OXA1L | Un_random | chrUn_Scaffold13443 | no prediction. Note: 770211 and 425893 have been discontinued. | no synteny; reciprocal BLAST to correct protein in several species |
| POLD1 | chrUn_AADN03023936:760-1113 | chrUn_Scaffold22687 | no prediction | no synteny; reciprocal BLAST to correct protein in several species |
| RBM23 | chrUn_AADN03015439:200-271 | chrUn_Scaffold25940 | no prediction | no synteny; reciprocal BLAST to correct protein in several species |
| SNRPA | chr20:10318343-10318579 (in proximity to TBC1D20, but separated by gap) | chrUn_Scaffold5494 | no prediction | correct suynteny partial with RAB4B |
| SUPT16H | chrUn_JH375572:3442-4462 | chrUn_Scaffold10624 | no prediction | no synteny; reciprocal BLAST to correct protein in several species |
