# Supplementary material for: A New Chicken Genome Assembly Provides Insight into Avian Genome Structure
Source: G3 (Bethesda). 2016 Nov 14;7(1):109–17. doi: 10.1534/g3.116.035923 (PMC5217101; doi:10.1534/g3.116.035923)
Supplement: Supplementary file 29 [file 109TableS7.docx]

**Table S7**. A summary of newly identified genetic linkage groups. Estimated cM distance and total markers counts are presented by linkage group. See methods for details of linkage group generation.

| **EL Linkage Group** | **cM** | **Total markers** |
| --- | --- | --- |
| E101 | 79 | 300 |
| E102 | 71 | 340 |
| E103 | 68 | 66 |
| E104 | 9 | 74 |
| E105 | 66 | 50 |
| E106 | 3 | 36 |
| E107 | 55 | 86 |
| E108 | 69 | 134 |
| E109 | 52 | 175 |
| E110 | 13 | 127 |
| E111 | 54 | 96 |
| E112 | 51 | 232 |
| E113 | 57 | 69 |
| E114 | 47 | 105 |
| E115 | 70 | 207 |
| E116 | 2 | 92 |
| E117 | 5 | 79 |
| E118 | 103 | 549 |
| E119 | 61 | 116 |
| E120 | 71 | 158 |
| E121 | 10 | 66 |
| E122 | 1 | 232 |
| E123 | 5 | 21 |
| E124 | 6 | 3 |
| E125 | 0 | 13 |
| E126 | 13 | 3 |
| E127 | 0 | 3 |
| E128 | 0 | 3 |
| E129 | 2 | 2 |
